# Neuroinflammation-mediated YKL-40 correlates with tau pathology and predicts longitudinal cognitive impairment and brain atrophy in Alzheimer’s disease, with hypertensive dependency

**DOI:** 10.3389/fnagi.2025.1630022

**Published:** 2025-08-06

**Authors:** Ya-Yu Wang, Man Zhang, Shu-Jian Chen, Wei Miao, Zhi-Xin Wang, Ya-Jun Zhou, Si-Qi Yu, Zhong-Wu Sun, Xia Zhou, Xian-Feng Yu, Xiao-Qun Zhu

**Affiliations:** Department of Neurology, The First Affiliated Hospital of Anhui Medical University, Hefei, China

**Keywords:** Alzheimer’s disease, astrocyte reactivity, YKL-40, neuroinflammation, hypertension

## Abstract

**Background:**

Neuroinflammation and hypertension are involved in Alzheimer’s disease (AD). However, their independent and additive impacts on astrocytes and AD-related pathologies have not been fully explored. Hence, this study investigated whether the associations between astrocyte reactivity, measured by cerebrospinal fluid (CSF), Chitinase 3-like protein 1 (CHI3L1/YKL-40), and AD-related pathologies were mediated by neuroinflammation and whether these associations were modified by hypertension. We also investigated the influence of hypertension on the relationship between baseline levels of CSF YKL-40 and longitudinal changes in cognitive function and brain structures.

**Methods:**

This study analyzed 288 participants from the AD Neuroimaging Initiative (ADNI) database. Multivariate linear regression, interaction, and subgroup analyses were conducted to explore the interrelationship between CSF YKL-40, AD biomarkers, neuroinflammation, cognitive function, and brain structures. Causal mediation analyses with 10,000 bootstrapped iterations were performed, using CSF YKL-40 as the independent variable and AD-related pathologies as the dependent variables, to explore mediation effects of neuroinflammation. Linear mixed-effects models were employed to study the associations between CSF YKL-40 and longitudinal changes in cognitive function and brain structures.

**Results:**

Higher baseline CSF YKL-40 levels were correlated with higher p-tau, t-tau, and neuroinflammatory biomarkers (ICAM1, VCAM1, sTNFR1, and sTNFR2), but with lower entorhinal cortex volume. Interaction showed that hypertension had a moderating influence on the associations between CSF YKL-40 and p-tau and t-tau. The significant associations of CSF YKL-40 with p-tau and t-tau were partially mediated by neuroinflammatory biomarkers (ICAM1, VCAM1, sTNFR1, and sTNFR2) in the whole sample (proportions: 13.0%∼78.8%). Similarly, the partial mediation effects of VCAM1, sTNFR1, and sTNFR2 on the aforementioned associations also existed in hypertensive subgroup (proportions: 17.9%∼50.3%). Additionally, higher baseline levels of CSF YKL-40 predicted faster decline in cognitive performance and brain atrophy (volumes of whole brain, hippocampus, entorhinal cortex, and middle temporal lobe) in the whole sample. Notably, subgroup analyses showed that the associations between higher CSF YKL-40 and faster brain atrophy were pronounced in hypertensive individuals.

**Conclusion:**

These findings suggest that neuroinflammation may mediate the relationship between astrocyte reactivity, measured by CSF YKL-40, and AD-related pathologies, with significant hypertensive dependency. Furthermore, elevated baseline CSF YKL-40 levels accelerated cognitive decline and brain atrophy, particularly in hypertensive individuals.

## Introduction

Alzheimer’s disease is pathologically characterized by the extracellular accumulation of amyloid beta (Aβ) plaques and intracellular tau neurofibrillary tangles. Neuroinflammation has been considered as the third core pathological feature of AD (Kinney et al., 2018; McGeer and Rogers, 1992). Neuroinflammation, triggered by microglial and astrocytic activation, occurs decades before the clinical manifestation of AD and is strongly linked to the misfolded and aggregated proteins (Steardo et al., 2015). Evidence from clinical studies demonstrated that inflammatory biomarkers were elevated in AD patients (Shen et al., 2019), and utilizing anti-inflammatory drugs might reduce the risk of AD (Wang et al., 2015). Astrocytes, as a key component of the neurovascular unit (Iadecola, 2017), are crucial for maintaining the integrity of the blood-brain barrier (BBB) (Luissint et al., 2012). Notably, astrocytes are important regulators of central nervous system inflammatory response and actively contribute to neuroinflammatory cascades in AD pathogenesis (Ferrari-Souza et al., 2022). Elucidating the astrocyte-neuroinflammation interplay may therefore provide potential clinical value for diagnosis and treatments for AD.

Chitinase 3-like protein 1 (CHI3L1/YKL-40) is almost exclusively expressed by astrocytes throughout neurodevelopment in human brain tissue (Connolly et al., 2023), and strongly colocalizes with the astrocyte marker Glial fibrillary acidic protein (GFAP) (Llorens et al., 2017; Querol-Vilaseca et al., 2017). It is being increasingly considered as a biomarker of reactive astrocytes in AD (Carter et al., 2019; Pelkmans et al., 2024). Previous research has shown that high levels of CSF YKL-40 are significantly associated with the accumulation of AD pathologies and increase the risk of AD dementia (Ferrari-Souza et al., 2022; Janelidze et al., 2018; Pelkmans et al., 2024; Vergallo et al., 2020; Warmenhoven et al., 2024). However, there are studies reporting somewhat contradictory findings that higher CSF YKL-40 predicts slower cognitive decline in AD dementia (Kester et al., 2015). Together, the contribution of astrocytic activity, as reflected by the elevated levels of CSF YKL-40, to the pathogenesis of AD and its value in monitoring the progression of AD may be complex and require further elucidation.

Additionally, numerous studies have pointed out that vascular risk factors, especially hypertension, were strongly associated with AD (Kivipelto et al., 2001; McGrath et al., 2017). In vitro and in vivo studies indicate that hypertension exacerbates amyloid and tau pathology, activates neuroinflammation, and accelerates cognitive decline (Bajwa and Klegeris, 2022; Denver et al., 2019). In a mouse APP/PS1 model of AD, decreases in Chi3l1/YKL-40 promote astrocytic Aβ phagocytosis, and mitigate the formation of misfolded proteins (Lananna et al., 2020). It is noteworthy that YKL-40 levels are elevated in patients with hypertension (Xu et al., 2016), and other chronic inflammatory conditions, such as diabetes, atherosclerosis (Rathcke and Vestergaard, 2006). Hypertension has the potential to cause damage to the cerebral microcirculation, decrease cerebral blood flow, and subsequently lead to ischemia, initiating a neuroinflammatory cascade (Snyder et al., 2017) that involves the activation of astrocytes and microglia, which release YKL-40 (Bonneh-Barkay et al., 2010) and sTREM2 (Brendel et al., 2017) as part of the tissue repair process. Taken together, the complex interrelationships between hypertension, neuroinflammation, and YKL-40, and their implications in the progression of AD, remain to be thoroughly explored.

The present study utilized the cross-sectional and longitudinal data with the aims to (1) ascertain the relationships of CSF levels of YKL-40 with neuroinflammation, AD core biomarkers (Aβ_42_ and p-tau [phosphorylated tau]), neurodegeneration (t-tau [total tau], MRI brain structures) and cognitive function; (2) explore whether AD modifiable risk factors (vascular risk factors, particularly hypertension) or non-modifiable risk factors (age, sex, apolipoprotein *E4* [*APOE*ε*4*] status) moderate these associations; (3) determine whether neuroinflammation serves as a mediating factor in the correlation between CSF levels of YKL-40 and AD-related pathologies; and (4) evaluate the predictive value of CSF level of YKL-40 for longitudinal changes in cognitive function and brain structures from the AD Neuroimaging Initiative (ADNI) database.

## Materials and methods

### Participants

The data utilized in this study were derived from the ADNI database (Weiner et al., 2013). The detailed inclusion and exclusion criteria in ADNI are publicly available at https://adni.loni.usc.edu/. A total of 288 participants with defined basic clinical features, CSF parameters, imaging analysis, and cognitive evaluation were enrolled, with an average follow-up time of 4.28 years (standard deviation [SD] = 3.75 years). Specifically, the participants were classified into 84 cognitively normal persons (CN, Mini-Mental State Examination [MMSE] > 24, Clinical Dementia Rating Sum of Boxes [CDR] = 0), 127 persons with mild cognitive impairment (MCI; MMSE > 24, CDR = 0.5) and 77 patients with AD dementia according to predefined criteria (Aisen et al., 2010). Individuals were defined as hypertensive if they reported a history of hypertension or using antihypertensive medication. Prior to enrollment, written informed consent was obtained from each participant or their legally authorized representative.

### Measurements of CSF biomarkers

CSF YKL-40 was quantified at the University of Washington using the MicroVue YKL-40 Enzyme-Linked Immunosorbent Assay (ELISA) (Quidel Corp.) (Craig-Schapiro et al., 2010). Concentrations of CSF Aβ_42_, p-tau, and t-tau were determined using multiple xMAP technology platforms (Luminex Corporation) and INNOBIA AlzBio3 kit (Ghent, Belgium) at the University of Pennsylvania (Shaw et al., 2009). Neuroinflammatory biomarkers in CSF, comprising of four anti-inflammatory molecules (soluble tumor necrosis factor receptor 1 [sTNFR1], sTNFR2, transforming growth factor-beta 1 [TGF-β1], interleukin-10 [IL-10]) and four pro-inflammatory molecules (intercellular cell adhesion molecule-1 [ICAM1], vascular cell adhesion molecule-1 [VCAM1], IL-6, and IL-7) were quantified using commercially available ELISA kits (Millipore Sigma, Burlington, MA) (Craig-Schapiro et al., 2011). According to the A/T scheme, participants were categorized as A + (CSF Aβ_42_ < 976.6 pg/mL) or T + (CSF p-tau > 21.8 pg/mL) based on established biomarker cutoffs for subsequent analyses (Hansson et al., 2018). The measurements of all CSF biomarkers were duplicated, and the mean values were calculated. Coefficients of variation for CSF analytes, both within and between lots, were less than 15%. The original data and detailed assay protocols are available in the ADNI database.

### Cognitive assessment

Cognitive function was evaluated through a range of scales. The MMSE and the AD Assessment Scale-Cognitive Subscale 13 (ADAS-13) were used to assess global cognitive function. Functional Assessment Questionnaire (FAQ) were used to evaluate the life quality. Domain-specific cognitive functions were assessed using neuropsychological test batteries, with composite scores reflecting episodic memory (MEM), language (LAN), executive function (EXF) and visuospatial function (VP) (Crane et al., 2012; Gibbons et al., 2012). Detailed information can be found in Supplementary Material 1. All evaluations were performed at baseline and at follow-up visits.

### Structural magnetic resonance imaging (MRI) measures

The protocols for MRI acquisition have been detailed in prior publications (Jack et al., 2008). Structural MRIs were acquired using the Siemens Verio 1.5 T or 3 T MRI Scanner. Cortical reconstruction and volumetric segmentation were automated using FreeSurfer software based on T1-weighted images to estimate the volumes of regional brain regions. We focus on the volumes of the whole brain, hippocampus, entorhinal cortex, and middle temporal lobe as the primary AD signature regions of interest (ROI). All MRI measures were averaged for the left and right hemispheres.

### Statistical analyses

Baseline characteristics were compared between different hypertensive status (hypertension versus normotension) using Mann-Whitney U tests for continuous and χ^2^-tests for categorical variables. Data of the CSF biomarkers exhibited a non-normal distribution and therefore log_10_-transformation was conducted for statistical analysis.

The first aim of this study was to assess the direct correlations between CSF levels of YKL-40 (independent variable) and CSF levels of AD biomarkers, neuroinflammatory biomarkers, brain structural MRI data and cognitive scores (dependent variables) at baseline. To achieve this, we conducted multivariate linear regression (MLR) analyses. Sensitivity analyses were performed by constructing various MLR models with adjustment of more potential confounding factors, including the level of CSF Aβ_42_, cognitive status and vascular risk factors (history of hypertension, diabetes, hyperlipidemia, stroke and smoking). Stratified analyses among different diagnostic (CN, MCI, and AD) and biological (A- versus A+, T- versus T+) and combined (A-CN, A-MCI, A + CN, A + MCI, etc.) subgroups were also conducted as the exploratory analyses. Moreover, interaction terms between baseline CSF levels of YKL-40 and Aβ status, sex, *APOE*ε*4* status, age, or vascular risk factors (hypertension and hyperlipidemia) were added to MLR models to test their influences on the aforementioned associations.

Next, mediation analyses, proposed by Baron and Kenny (1986), were used to assess whether CSF levels of neuroinflammatory biomarkers could not, or could partially or entirely mediate any significant association between CSF YKL-40 levels and AD-related pathologies. For each mediation model, we used the CSF level of YKL-40 as an independent variable and AD-related pathologies as the dependent variables. The Sobel test (Imai et al., 2010) was employed to estimate the direct and indirect effects, as well as the proportion mediated, with the significance of these effects established through 10,000 bootstrap replications.

Finally, linear mixed effects (LME) models were conducted to identify whether the baseline CSF levels of YKL-40 could predict longitudinal changes in cognitive function and MRI brain structures. To facilitate the analysis, CSF YKL-40 was categorized as a dichotomous variable (low versus high) determined by the median concentration with a threshold of 390 ng/mL. These LME models were created using cognitive functions and MRI brain structures as dependent variables. Time since baseline (years), CSF YKL-40 (continuous variable), and their interaction were included as fixed effects. Sex, age, education, *APOE*ε*4* carrier status, intracranial volume ([ICV], when appropriate), and their interactions with time were used as covariates with subject-specific intercepts and slopes.

All regression analyses were adjusted for covariates, including sex, age, education, and *APOE*ε*4* status. Additionally, ICV was also included as a covariate in the analyses of the structural MRI data. A two-sided *p*-value < 0.05 was considered statistically significant. False discovery rate (FDR) was used for multiple corrections where specifically noted (Benjamini and Hochberg, 1995). R version 4.4.1 (packages including “lm,” “ggplot2,” “car,” “mediate,” and “nlme”) and IBM SPSS Statistics version 27.0.1 were used for statistical analyses and figure preparation.

## Results

### Baseline participants characteristics

The demographic features, CSF biomarker levels, cognitive scores, and MRI brain structures data stratified by hypertensive status are presented in Table 1. A total of 288 participants were included in this study, of whom 131 (46.2%) had hypertension. The whole sample had a male proportion of 41.0%, and an *APOE* ε*4* carrier proportion of 50.0%. The mean (±SD) values of the age, years of education, and CSF level of YKL-40 (ng/ml) were 74.97 (±7.36), 15.56 (±2.97), and 406.57 (±133.31), respectively. There were higher CSF levels of sTNFR1 and sTNFR2 in the hypertensive subgroup (HTN+) than in the normotensive subgroup (HTN-) (*p* < 0.05). However, we found no difference in demographic features, other biomarkers or cognitive function between the two subgroups at baseline.

**TABLE 1:** Demographic and clinical characteristics of participants in the study.

Variables	Total (*n* = 288)	Hypertension (*n* = 131)	Normotension (*n* = 157)	*P*-value
Age, mean (SD), y	74.97 (7.36)	75.92 (7.05)	74.18 (7.52)	0.069
Male, n (%)	118 (40.97)	54 (41.22)	64 (40.76)	0.937
Education, mean (SD), y	15.56 (2.97)	15.63 (3.05)	15.49 (2.90)	0.619
*APOE ε4* carriers (%)	144 (50.00)	63 (48.09)	81 (51.59)	0.554
YKL-40, mean (SD), ng/ml	406.57 (133.31)	424.24 (136.45)	388.18 (124.68)	0.162
**CSF AD biomarkers, mean (SD), pg/ml**				
Aβ_42_	961.22 (574.23)	987.45 (564.03)	939.34 (581.70)	0.371
p-tau	29.27 (13.86)	30.00 (15.31)	28.67 (12.48)	0.812
T-tau	299.54 (119.19)	306.03 (130.67)	294.12 (108.40)	0.759
**CSF neuroinflammatory biomarkers, mean (SD), pg/ml**				
sTNFR1	874.82 (230.71)	900.52 (231.46)	853.37 (227.87)	**0.042**
sTNFR2	1059.48 (387.00)	1109.88 (493.05)	1017.42 (260.80)	**0.047**
TGF-β1	106.58 (39.72)	106.51 (38.10)	106.64 (41.03)	0.949
IL-10	5.75 (2.68)	5.78 (2.96)	5.73 (2.43)	0.384
ICAM1	374.79 (191.05)	378.76 (193.73)	371.49 (188.72)	0.893
VCAM1	43047.53 (23033.19)	43816.10 (22429.97)	42406.32 (23505.47)	0.440
IL-6	5.14 (4.79)	5.55 (5.17)	4.79 (4.42)	0.450
IL-7	1.29 (1.76)	1.20 (1.34)	1.37 (2.14)	0.318
**MRI measures (mm^3^)**				
Hippocampus	6488.55 (1169.38)	6444.97 (1140.61)	6524.87 (1191.60)	0.602
Entorhinal cortex	3337.46 (840.30)	3307.00 (858.15)	3362.85 (824.28)	0.488
Mid temporal	18683.23 (3293.88)	18340.28 (3116.37)	18969.03 (3408.54)	0.146
Whole brain	983889.73 (105778.04)	979612.18 (95668.20)	987341.26 (113161.98)	0.644
ICV	1557361.91 (171809.40)	1556777.63 (16058.20)	1557849.43 (180644.01)	0.967
**Cognitive scores**				
MMSE	26.63 (2.60)	26.68 (2.70)	26.58 (2.52)	0.583
MEM	–0.05 (0.69)	–0.04 (0.69)	–0.06 (0.69)	0.839
LAN	0.27 (0.59)	0.26 (0.63)	0.28 (0.55)	0.615
EF	0.16 (0.65)	0.10 (0.64)	0.21 (0.64)	0.172
VP	–0.05 (0.42)	–0.10 (0.47)	0.003 (0.37)	0.168
FAQ	5.18 (6.73)	5.59 (7.08)	4.85 (6.40)	0.521
ADAS13	18.53 (9.38)	19.17 (9.00)	18.01 (9.65)	0.208

Categorical variables are depicted as counts and percentages, while continuous variables are expressed as mean (SD). Significant at the level of p < 0.05 were shown in bold. SD, standard deviation; *APOE ε4*, apolipoprotein *E4*; YKL-40, chitinase-3-like protein 1; CSF, cerebrospinal fluid; Aβ, amyloid-β; p-tau, phosphorylated tau; t-tau, total tau; sTNFR, soluble tumor necrosis factor receptor; TGF, transforming growth factor; IL, interleukin; ICAM1, intercellular cell adhesion molecule-1; VCAM1, vascular cell adhesion molecule-1; MRI, Magnetic resonance imaging; ICV, Intracranial volume; MMSE, Mini-Mental State Examination; MEM, memory function; LAN, language; EF, executive function; VP, visuospatial function; FAQ, Functional Assessment Questionnaire; ADAS13, Alzheimer’s disease Assessment Scale 13.

### Interrelationship of baseline CSF levels of YKL-40 with various biomarkers, cognitive function, and brain structures

**FIGURE 1:**
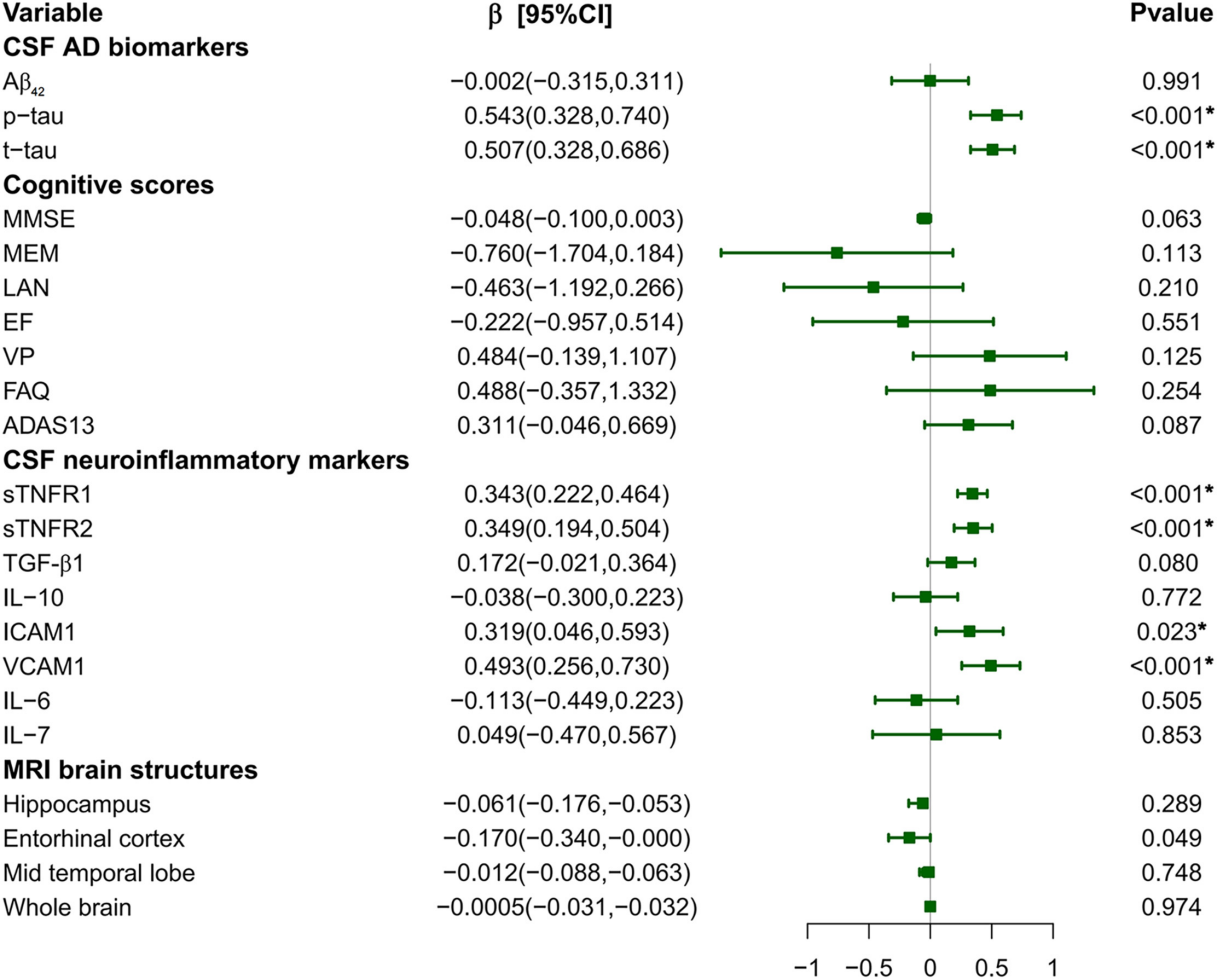
Associations of CSF YKL-40 with AD biomarkers, neuroinflammatory markers, cognition, and MRI brain structures. *P*-values were obtained by multiple linear regressions models adjusted for age, sex, years of education, *APOE ε4* status, and additional adjustment for ICV in the analyses of the structural MRI data. “*” indicated a significant *p*-value after false discovery rate correction. CSF, cerebrospinal fluid; CI, confidence interval; Aβ, amyloid-β; p-tau, phosphorylated tau; t-tau, total tau; MMSE, Mini-Mental State Examination; MEM, memory function; LAN, language; EF, executive function; VP, visuospatial function; FAQ, Functional Assessment Questionnaire; ADAS13, Alzheimer’s disease Assessment Scale 13; sTNFR, soluble tumor necrosis factor receptor; TGF, transforming growth factor; IL, interleukin; ICAM1, intercellular cell adhesion molecule-1; VCAM1, vascular cell adhesion molecule-1; MRI, magnetic resonance imaging.

As shown in Figure 1 and Supplementary Table 1 in Supplementary Material 1, higher baseline levels of CSF YKL-40 were associated with higher levels of p-tau (β = 0.534, *p* < 0.001) and t-tau (β = 0.507, *p* < 0.001). Besides, we found that baseline levels of CSF YKL-40 were positively associated with sTNFR1 [β = 0.343, *p* < 0.001], sTNFR2 [β = 0.349, *p* < 0.001], ICAM1 [β = 0.319, *p* = 0.023], and VCAM1 [β = 0.493, *p* < 0.001]. After FDR correction, the *p*-values presented for the above results are all less than 0.05. As for MRI brain structures, the negative association between baseline levels of CSF YKL-40 and entorhinal cortex volume was at the threshold of significance (β = −0.128, *p* = 0.049), but did not remain significant following FDR correction. However, no significant associations between baseline levels of CSF YKL-40 and cognitive function were found. Consistently, the significant associations between CSF YKL-40 with p-tau, t-tau, and the four neuroinflammatory biomarkers (sTNFR1, sTNFR2, ICAM1, and VCAM1) were barely changed in sensitivity analyses using different MLR models (Supplementary Table 1 in Supplementary Material 1). Besides, exploratory stratified analyses showed that these significant associations between CSF YKL-40 and sTNFR1, sTNFR2, and VCAM1 were more pronounced in the T + subgroup than in the T- subgroup. Similar results were obtained in the A + MCI subgroup (Supplementary Tables 1–6 in Supplementary Material 2).

**FIGURE 2:**
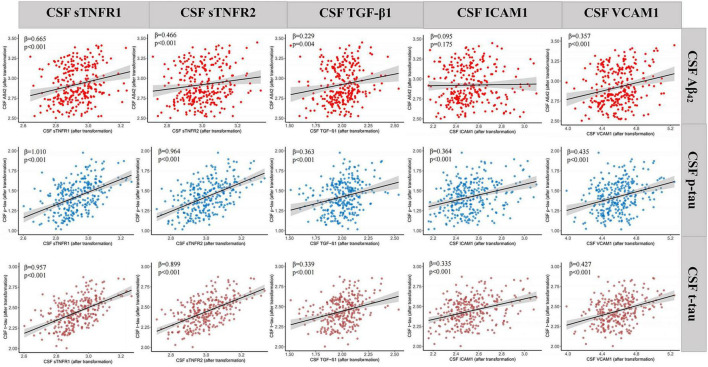
Associations between CSF neuroinflammatory biomarkers and AD biomarkers. The *x* axis represents CSF neuroinflammatory biomarkers (sTNFR1, sTNFR2, TGF-β1, ICAM1 and VCAM1) and the *y* axis represents CSF AD biomarkers (Aβ_42_, p-tau and t-tau). *P*-values were obtained by multiple linear regressions models adjusted for age, sex, years of education and *APOE ε4* status. CSF, cerebrospinal fluid; Aβ, amyloid-β; p-tau, phosphorylated tau; t-tau, total tau; sTNFR, soluble tumor necrosis factor receptor; TGF, transforming growth factor; ICAM1, intercellular cell adhesion molecule-1; VCAM1, vascular cell adhesion molecule-1.

Multivariate linear regression models were also utilized to explore the associations of CSF neuroinflammatory biomarkers (independent variables) with AD biomarkers, MRI brain structures, and cognitive function (dependent variables) (Supplementary Table 2 in Supplementary Material 1). The results indicated that CSF neuroinflammatory biomarkers, including sTNFR1, sTNFR2, TGF-β1, ICAM1 and VCAM1, had positive correlations with CSF AD biomarkers (Aβ_42_, p-tau, and t-tau, Figure 2), even after FDR correction (Supplementary Table 2 in Supplementary Material 1). However, the negative correlations of CSF IL-7 level with MMSE (β = −0.888, *p* = 0.018), MEM (β = −0.229, *p* = 0.018) and ADAS13 (β = 0.072, *p* = 0.045), and the negative association between CSF level of ICAM1 and hippocampus (β = −0.051, *p* = 0.046) did not survive FDR correction. Additionally, no significant correlations were observed for IL-6 and IL-10 with CSF AD biomarkers.

### Modifying effect of AD risk factors

**FIGURE 3:**
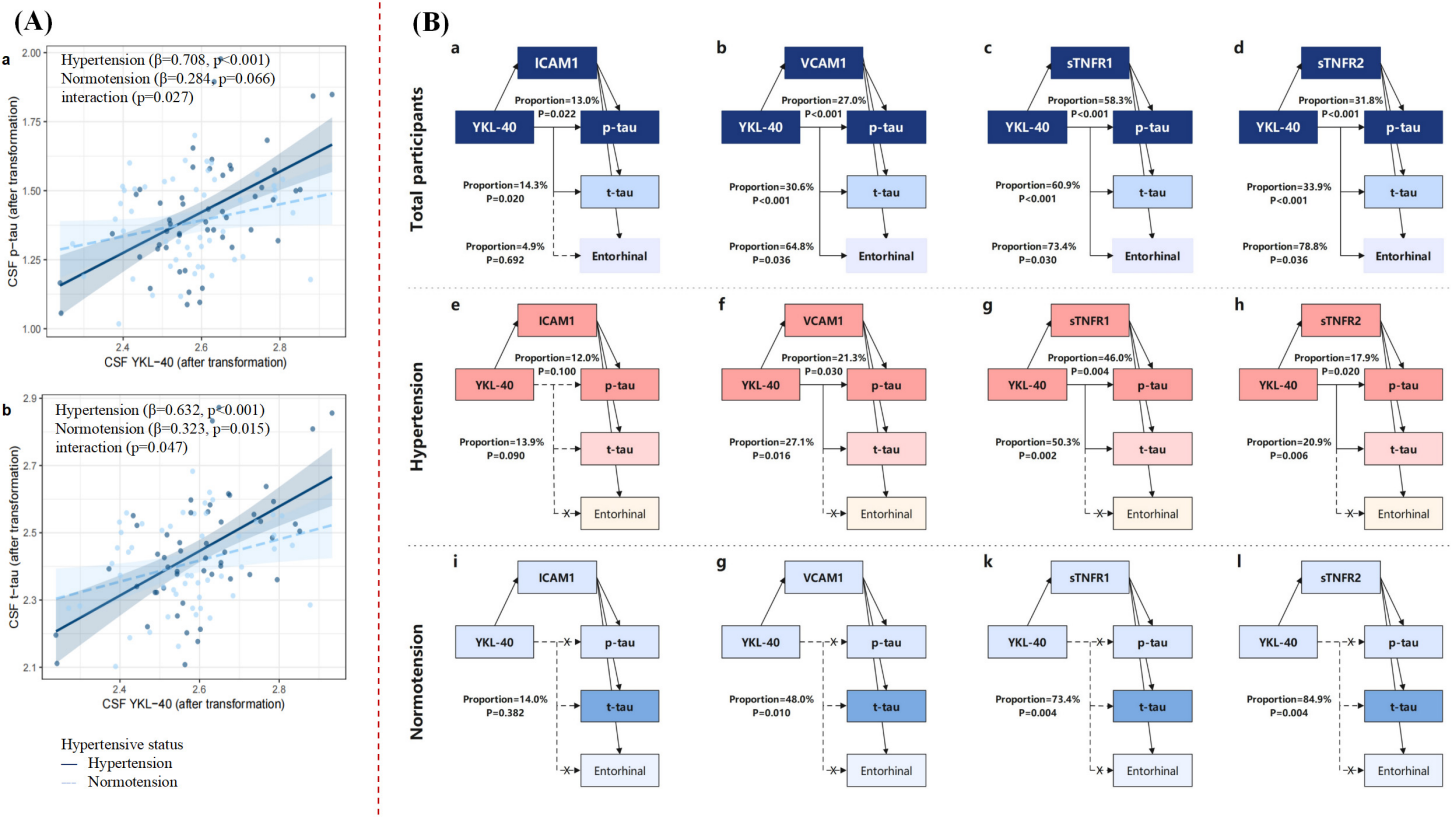
**(A)** Interaction effects of CSF YKL-40 and hypertensive status on CSF p-tau and t-tau. **(B)** Mediation effects of CSF neuroinflammatory biomarkers on the associations between CSF YKL-40 and p-tau, t-tau and entorhinal cortex volume. **(A)** Shows the significant interaction effects of CSF YKL-40 × hypertensive status on CSF p-tau and t-tau. In the stratified analyses, the significant association between CSF YKL-40 and p-tau was only observed in the hypertensive subgroup, not in the normotensive subgroup [**(A)**.a]. Similarly, the significant association between CSF YKL-40 and t-tau was found both in the hypertensive and the normotensive subgroups [**(A)**.b]. Interaction models were adjusted by age, sex, years of education and *APOE ε4* status. **(B)** Shows mediation effects in the whole sample [**(B)**.a–d], hypertensive [**(B)**.e–h] and normotensive [**(B)**.i–l] groups, respectively. The “×” signified the absence of a significant correlation with CSF YKL-40, and as such, no additional mediation analysis was performed. The dotted line indicated that the direct effect was not significant (*P* ≥ 0.05). Mediation analyses with 10,000 bootstrapped iterations were used to examine the mediation effects of CSF neuroinflammatory biomarkers on p-tau, t-tau and entorhinal cortex volume. Each path of the model was adjusted for age, sex, years of education, *APOE ε4* status, and additional adjustment for ICV in the analyses of entorhinal cortex volume. CSF, cerebrospinal fluid; p-tau, phosphorylated tau; t-tau, total tau; ICAM1, intercellular cell adhesion molecule-1; VCAM1, vascular cell adhesion molecule-1; sTNFR, soluble tumor necrosis factor receptor.

Next, we investigated whether the observed significant associations were modified by vascular risk factors, Aβ status, *APOE*ε*4* status, sex, or age. There were significant interaction effects between CSF levels of YKL-40 and hypertension on p-tau (β = 0.444, *p* = 0.027) and t-tau (β = 0.347, *p* = 0.047) (Figure 3A and Supplementary Table 3 in Supplementary Material 1). In subsequent stratified analyses, we found that higher CSF levels of YKL-40 were associated with higher levels of p-tau in the HTN + subgroup (β = 0.708, *p* < 0.001) rather than in the HTN- subgroup (β = 0.284, *p* = 0.066) (Figure 3A and Supplementary Table 4 in Supplementary Material 1). Nevertheless, a significant association with CSF t-tau was observed in both the HTN + (β = 0.632, *p* < 0.001) and the HTN- subgroups (β = 0.323, *p* = 0.015) (Figure 3A and Supplementary Table 4 in Supplementary Material 1). Notably, the significant associations between the CSF levels of YKL-40 and p-tau, t-tau, or neuroinflammatory biomarkers were not moderated by factors such as hyperlipidemia, sex, age, *APOE*ε*4* status, or Aβ status (Supplementary Tables 4, 5 in Supplementary Material 1).

### Causal mediation analyses

The primary analyses revealed the following significant associations: (1) CSF YKL-40 is positively correlated with p-tau, t-tau, and entorhinal cortex volume; (2) CSF YKL-40 exhibits significant positive associations with these neuroinflammatory biomarkers (sTNFR1, sTNFR2, ICAM1, and VCAM1); (3) these neuroinflammatory biomarkers are significantly correlated with p-tau, t-tau, and entorhinal cortex volume. Given the significant pairwise correlations among these variables, we conducted exploratory mediation analyses among the participants to assess whether these correlations between CSF YKL-40 level and p-tau, t-tau, and entorhinal cortex volume were mediated by neuroinflammatory biomarkers. As shown in Figure 3B and Supplementary Table 6 in Supplementary Material 1, the mediation analyses indicated that the associations of CSF levels of YKL-40 with p-tau and t-tau were partially mediated by ICAM1 (p-tau: proportion = 13.0%, *p* = 0.022; t-tau: proportion = 14.3%, *p* = 0.020), VCAM1 (p-tau: proportion = 27.0%, *p* < 0.001; t-tau: proportion = 30.6%, *p* < 0.001), sTNFR1 (p-tau: proportion = 58.3%, *p* < 0.001; t-tau: proportion = 60.9%, *p* < 0.001), and sTNFR2 (p-tau: proportion = 31.8%, *p* < 0.001; t-tau: proportion = 33.9%, *p* < 0.001). Additionally, the association between CSF YKL-40 levels and entorhinal cortex volume was partially mediated by VCAM1 (proportion = 64.8%, *p* = 0.036), sTNFR1 (proportion = 73.4%, *p* = 0.030), and sTNFR2 (proportion = 78.8%, *p* = 0.036) before controlling for Aβ_42_, whereas the partial mediation effects disappeared after including Aβ_42_ as a covariate in mediation analyses (Supplementary Figure 1 in Supplementary Material 1).

The interaction effect indicated that hypertension may be a potential modulator for the association between CSF YKL-40 levels and tau pathologies. Therefore, we further explored whether hypertension influenced the aforementioned mediation analyses by categorizing the sample into two subgroups (HTN- versus HTN+). The partially mediation of VCAM1, sTNFR1, and sTNFR2 on the associations between CSF YKL-40 levels and p-tau and t-tau were only reproduced in the HTN + subgroup (Figure 3B and Supplementary Table 7 in Supplementary Material 1). The mediation remained significant even after additional adjustment for Aβ_42_ (Supplementary Figure 1 in Supplementary Material 1). In contrast, CSF YKL-40 level was directly related to t-tau without mediation effects in the HTN- group (Figure 3B and Supplementary Table 8 in Supplementary Material 1). Subsequent exploratory analyses conducted among different diagnostic and biological subgroups and combined with hypertensive status showed that the mediation of sTNFR1 and sTNFR2 were particularly robust in MCI, A+, T+, A + MCI, and T + HTN + subgroups (Supplementary Tables 7–12 in Supplementary Material 2).

### Associations of baseline CSF YKL-40 levels with longitudinal changes in cognitive function and MRI brain structures

**FIGURE 4:**
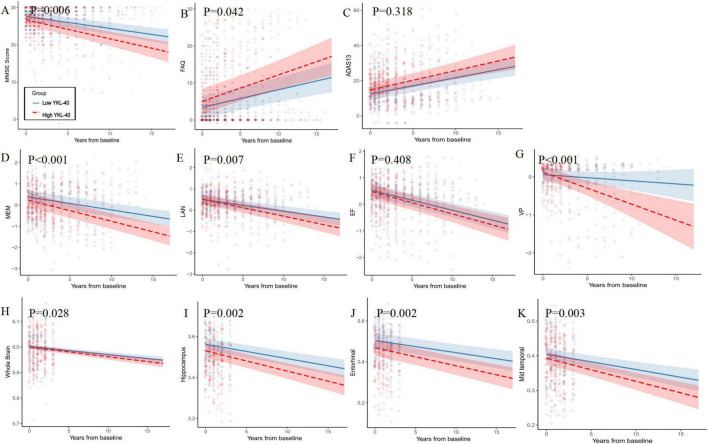
Baseline CSF YKL-40 and longitudinal changes in cognitive function and MRI brain structures in the whole sample. The mixed-effect models were fit using continuous CSF YKL-40 values, adjusting for age, sex, years of education, *APOE ε4* status and ICV (when appropriate). For better illustration, the plots displayed the trajectories for individuals with high and low CSF YKL-40 (≥ 390 ng/ml VS. < 390 ng/ml) for longitudinal cognitive function as well as for MRI brain structures. We found protective roles of low baseline levels of CSF YKL-40 in preventing decline of cognitive functions, including MMSE **(A)**, FAQ **(B)**, MEM **(D)**, LAN **(E)**, VP **(G)**, as well as MRI brain structures, including volumes of whole brain **(H)**, hippocampus **(I)**, Entorhinal cortex **(J)** and mid temporal lobe **(K)**, but not ADAS13 **(C)** or EF **(F)**. CSF, cerebrospinal fluid; MMSE, Mini-Mental State Examination; FAQ, Functional Assessment Questionnaire; ADAS13, Alzheimer’s disease Assessment Scale 13; MEM, memory function; LAN, language; EF, executive function; VP, visuospatial function.

Limited by CSF AD biomarkers, LME models were only used to explore the associations between baseline levels of CSF YKL-40 and longitudinal changes in cognitive function and MRI brain structures. In the whole sample, higher baseline levels of CSF YKL-40 were associated with faster rates of decrease in MMSE (β = −0.185, *p* = 0.006), MEM (β = −0.039, *p* < 0.001), LAN (β = −0.025, *p* = 0.007), VP (β = −0.068, *p* = 0.002), and increase in FAQ (β = 0.240, *p* = 0.042). As expected, higher baseline levels of CSF YKL-40 were also linked to more rapid atrophy in the volumes of whole brain (β = −0.001, *p* = 0.028), hippocampus (β = −0.003, *p* = 0.002), entorhinal cortex (β = −0.0002, *p* = 0.002) and middle temporal lobe (β = −0.002, *p* = 0.003) (Figure 4 and Supplementary Table 9 in Supplementary Material 1).

Intriguingly, some longitudinal associations were more pronounced in the HTN + subgroup (Supplementary Figure 2 and Supplementary Table 10 in Supplementary Material 1). More precisely, the associations between baseline levels of YKL-40 and longitudinal changes of FAQ (β = 0.631, *p* < 0.001), MEM (β = −0.305, *p* < 0.001), and AD signature ROI volume (whole brain: β = −0.001, *p* = 0.001; hippocampus: β = −0.004, *p* < 0.001; entorhinal cortex: β = −0.005, *p* < 0.001, and middle temporal lobe: β = −0.004, *p* < 0.001, respectively) were significant only in the HTN + subgroup (Supplementary Figure 2 and Supplementary Table 10 in Supplementary Material 1). The subgroup analyses yielded generally similar results, indicating that those significant correlations with MMSE, LAN, or VP did not appear to be affected by the hypertensive status (Supplementary Figure 3 and Supplementary Table 11 in Supplementary Material 1). Additionally, according to the clinical diagnosis and A status, we found that higher baseline levels of CSF YKL-40 were associated with faster atrophy in the volumes of whole brain, hippocampus, and mid temporal lobe in the A + CN but not in the A-CN subgroup. Combining hypertension with T status, faster atrophy in the volumes of whole brain, hippocampus, and mid temporal lobe were replicated in the T + HTN + subgroup rather than in the T- HTN-, T-HTN+, or T + HTN- subgroups (Supplementary Tables 13–18 in Supplementary Material 2).

## Discussion

**FIGURE 5:**
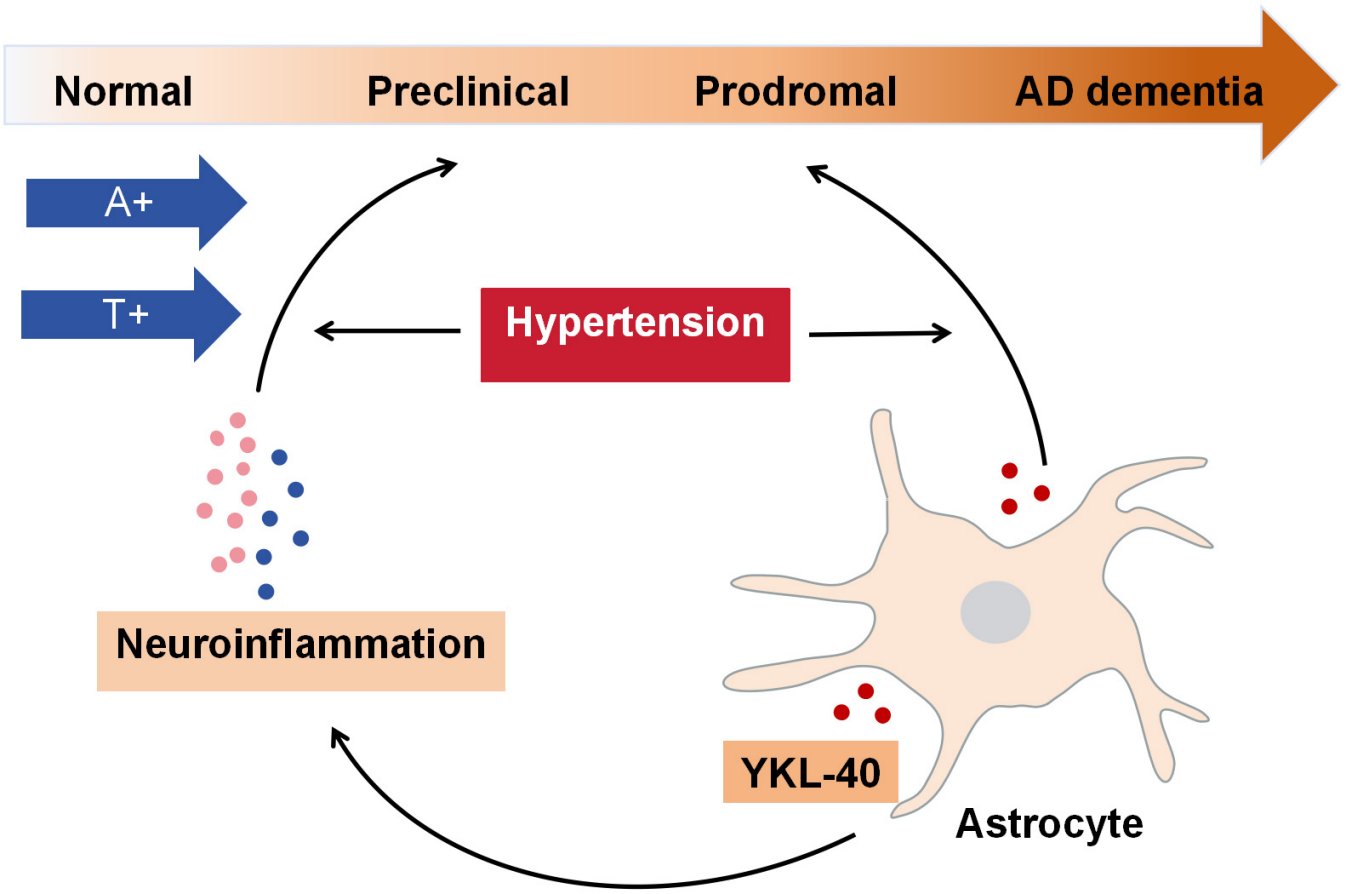
Interlinking schematic among CSF YKL-40, neuroinflammation, hypertension and AD-related pathologies. The findings in the current study suggest that hypertension and neuroinflammation interact, contributing to the associations between astrocyte, as reflected by CSF YKL-40, and AD-related pathologies, especially in the preclinical and prodromal stages of AD.

This study was the first to systematically explore the interrelationships of CSF YKL-40 with AD biomarkers, neuroinflammation, cognitive function and brain structures, and elucidate the additive effects of neuroinflammation and hypertension on the associations between astrocyte reactivity and AD-related pathologies. Overall, these results suggest that specific neuroinflammatory biomarkers might mediate the associations between CSF YKL-40 and p-tau and t-tau with hypertensive dependency, particularly in the preclinical and prodromal stages of AD (Figure 5). Specifically, the key findings are that: (1) higher baseline levels of CSF YKL-40 are positively related to p-tau and t-tau; (2) hypertensive status modifies the significant associations between CSF YKL-40 and p-tau and t-tau; (3) the mediation of VCAM1, sTNFR1 and sTNFR2 on abovementioned significant associations persist in the whole sample and individuals with hypertension; (4) higher baseline levels of CSF YKL-40 interact with hypertension to promote a faster decline in cognitive function and atrophy in hippocampal volume, entorhinal volume, middle temporal lobe volume, and whole-brain volume. Our results indicate that higher levels of CSF YKL-40 may promote AD progression, and constitute a reasonable pathway to connect the effects of hypertension and neuroinflammation with astrocyte reactivity in contributing to AD pathogenesis.

CSF Chitinase 3-like protein 1 (CHI3L1/YKL-40) is identified as a significant indicator of astrocyte reactivity (Carter et al., 2019). An increasing amount of evidence indicates that the positive correlations between CSF YKL-40 levels and tau pathologies, but not Aβ (Ferrari-Souza et al., 2022; Janelidze et al., 2018; Pelkmans et al., 2024). Pelkmans et al. (2024) reported that YKL-40 was released into the CSF slightly later in the AD cascade, specifically mediating the link between the downstream p-tau induced by Aβ and the neuronal damage induced by tau protein, whereas it did not mediate the association between soluble and insoluble Aβ aggregates. GFAP, another promising biomarker of astrocytic activation, was considered to be predominantly a response to Aβ pathology (Pelkmans et al., 2024). Our results were consistent with these findings that CSF YKL-40 was significantly associated with p-tau and t-tau. Similarly, the positive associations between CSF YKL-40 and p-tau and t-tau were particularly robust in the A + MCI and the T + subgroups in our exploratory analyses, which favored the hypothesis that CSF YKL-40 might contribute to tau accumulation secondary to Aβ plaques in the downstream AD cascade. In contrast, the EMIF-AD Multimodal Biomarker Discovery study revealed an association between YKL-40 and Aβ only in the non-demented population (Bos et al., 2019). A postmortem study found that YKL-40 was independent of tau in other neurodegenerative diseases (Querol-Vilaseca et al., 2017), which indicated that the astrocyte activity as measured by the CSF levels of YKL-40 might not be exclusively influenced by amyloid-tau dichotomy (Pelkmans et al., 2024; Salvadó et al., 2023). Besides, the marginally negative correlation between baseline CSF YKL-40 and the entorhinal cortex volume did not survive FDR correction in our results, warranting replication and validation in a larger sample. However, an animal experiment revealed that calcium-binding protein centrin-2-positive astrocytes were distributed in clusters within the entorhinal cortex and co-localized with YKL-40, providing a possible explanation for our results (Degl’Innocenti et al., 2024). Nevertheless, it should be noted that the choice of brain regions in our mediation analyses was to some extent guided by statistical considerations, which may lead to insufficient exploration of the mechanisms in crucial biological regions such as the hippocampus.

This study further proves that astrocytic reactivity with increased neuroinflammation play a prominent role in AD pathologies (Sofroniew and Vinters, 2010). We observe a robust correlation between CSF YKL-40 and ICAM1, VCAM1, sTNFR1, and sTNFR2. Simultaneously, these neuroinflammatory biomarkers partially mediate the effects of YKL-40 on tau pathology. These results reinforce the value of neuroinflammatory biomarkers to establish a more precise AD diagnostic framework in the clinic and provide a new perspective on understanding how astrocyte affects tau pathology. Of importance, the mediation analysis in this study is based on observational data and these CSF biomarkers (YKL-40, sTNFR1, sTNFR2, ICAM1, and VCAM1) may reflect overlapping aspects of a shared neuroinflammatory process, with the results reflecting correlation rather than causation. Further additional *in vitro* and *in vivo* studies are needed to verify the sequential and mechanistic associations between these molecules. Nevertheless, our findings suggest that interventions targeting astrocyte activation and neuroinflammation may benefit the prevention of tau pathology.

To the best of our knowledge, this is the first observational study to explore the interaction of hypertension and neuroinflammation on the associations between astrocyte reactivity and AD-related pathologies. We observed that hypertension reinforced the positive correlation between CSF YKL-40 and p-tau and t-tau in our interaction and subgroup analyses. Previous studies have shown that YKL-40 levels were elevated in hypertensive patients (Xu et al., 2016). Although our results revealed no difference in baseline levels of YKL-40 between the hypertensive and normotensive subgroups, further analyses indicated that the partial mediating effects of neuroinflammation (VCAM1, sTNFR1, and sTNFR2) on the associations between CSF YKL-40 and p-tau and t-tau were replicated in the HTN + subgroup rather than in the HTN- subgroup. An experiment investigating the additional or synergistic effects between AD and hypertension in rat models revealed that hypertension increased neuroinflammation (triggered by microglia and astrocytes) and tau pathology with minimal effect on Aβ pathology, which supported our results (Denver et al., 2019). Thus, these data seem to confirm this assumption that hypertension and astrocyte-driven neuroinflammation may interact to exacerbate AD pathologies (Bajwa and Klegeris, 2022; Nelson et al., 2014). However, the modification effects of other AD risk factors, such as age, sex, or *APOE*ε*4* status, are not observed in this study. A potential explanation for the lack of interaction may be the relatively limited sample size. Further studies are warranted to validate our findings.

Existing clinical evidence has suggested that an elevated levels of soluble cell adhesion molecules (CAMs), hallmarks of vascular endothelial dysfunction (Lee and Benveniste, 1999), are correlated with numerous vascular risk factors (Hwang et al., 1997), dementia (Drake et al., 2021), and immune-mediated diseases (Kong et al., 2018). Nevertheless, the underlying mechanism of its involvement in the pathogenesis of AD is not fully understood. Earlier studies on which CAMs were most relevant to cognition have yielded inconsistent results (Drake et al., 2021; Nielsen et al., 2007), with our present research suggesting a preference for VCAM1. Another cohort study from ADNI observed a synergistic effect of CAMs and AD pathology on the clinical severity of AD (Drake et al., 2021). Yousef et al. (2019) found that glial activation in the hippocampus of aging mice was associated with focal upregulation of VCAM1 on the luminal side adjacent to the BBB. Additionally, a study from postmortem brain tissue from AD patients found that perivascular astrocytes in the white matter exhibit high CHI3L1 expression, further suggesting the involvement of YKL-40 in BBB disruption (Moreno-Rodriguez et al., 2020). A well-established fact is that endothelial dysfunction and chronic vascular inflammation are critical pathophysiological changes in hypertension. In the present study, we observed that CAMs mediated the associations between YKL-40 and p-tau and t-tau, and the mediation effects of VCAM1 were particularly robust in hypertensive patients. Altogether, these findings support the hypothesis that hypertension may influence the neuroinflammatory pathological cycle of astrocyte activation, subsequently inducing endothelial dysfunction and further aggravating tau pathology in AD.

This work identified sTNFRs as another potential mediating factor in the association between CSF YKL-40 and tau pathology. TNF-α, a biomarker of proinflammatory cytokine and neurodegenerative diseases (McCoy and Tansey, 2008), is secreted by activated astrocytes and microglia. TNFR could be stimulated upon binding to soluble TNF and cleaved to yield sTNFR. The measurement of sTNFRs provides a more sensitive determination of TNF-signaling activity than the direct measurement of TNF-α (Kreuzer et al., 1996). In line with our results, a prior study had demonstrated positive associations between sTNFRs and Aβ, as well as tau pathology in CSF (Buchhave et al., 2010). Furthermore, we and other previous studies found that sTNFRs were elevated in hypertensive patients compared with normotensive patients (Verma et al., 2019). These findings were supported by AD rat models showing that administration of telmisartan (an AT1 receptor blocker) can decrease TNF-α mRNA expression in the hippocampus and ameliorate spatial memory (Shindo et al., 2012). By coincidence, our results showed that the mediation effects of sTNFRs were notably stronger pronounced in the hypertensive group. The exact mechanism by which hypertension influences the mediation effects of sTNFRs was not entirely elucidated. Considering sTNFRs as an important signaling mechanism leading to inflammation and endothelial dysfunction, we speculate that microvascular injury and BBB disruption may be the key underlying mechanisms. Additionally, Shen et al. (2019) found that the levels of plasma TNF-α were increased and vascular endothelial growth factor A were decreased in MCI patients compared to cognitively healthy controls, while plasma sTNFR-1 were inversely correlated with MoCA scores in MCI patients. These findings supported neuroinflammatory and vascular hypothesis for dementia. However, no significant correlation between CSF sTNFRs and cognitive scores was found in the present study. The reason for the inconsistent results may be the heterogeneity of the study population, sample size, differences in the measurement of the biomarker or other confounding factors.

We also systematically reviewed the previous literature and found that previous studies frequently, but not consistently reported the relation of IL-6, IL-7, or IL-10 to AD biomarkers, neuroinflammation, cognitive performance, and brain structures (Cisbani et al., 2020; Hazen et al., 2020; Janelidze et al., 2018; Marsland et al., 2015). In line with our results, a prior study using ADNI database had also demonstrated that there was no significant associations of CSF IL-6, IL-7, or IL-10 with CSF AD biomarkers (Xu et al., 2021). But we observed suggestive inverse associations between CSF IL-7 and MMSE, MEM and ADAS13. The biological mechanism between these correlations is currently unknown, although it can be speculated that various physiologic, medical, environmental, or lifestyle factors could impact on inflammation and AD pathologies. The potential biological mechanism remains to be clarified in the future.

Furthermore, a better understanding of the predictive role of YKL-40 in AD progression will help to develop reliable prognostic and outcome indicators in clinical practice. We therefore evaluated the utility of baseline CSF YKL-40 levels in predicting longitudinal changes of cognitive function and brain atrophy. Consistent with previous studies, our longitudinal investigations show that higher CSF YKL-40 levels are associated with fast decline in cognitive performance (Warmenhoven et al., 2024). Other studies have also reported that higher levels of CSF YKL-40 increased risk of developing AD dementia in non-demented elderly (Janelidze et al., 2018). A Chinese community-based aging cohort demonstrated that higher cortical tau tangle load and lower temporal cortical thinning were more pronounced in the presence of higher levels of astrocyte activation, as measured by plasma GFAP (Guo et al., 2024). Likewise, our study observes significant associations between higher baseline CSF levels of YKL-40 and accelerated rates of atrophy in AD signature ROI volumes (whole brain, hippocampus, entorhinal cortex, and middle temporal lobe), which are vulnerable regions associated with dementia. Aligning with previous research that hypertension was related to global gray matter atrophy (Glodzik et al., 2012), our study found that hypertension had additional deleterious effects on the typical atrophy pattern linking to CSF YKL-40. These findings underscored that CSF YKL-40 and hypertension could contribute to cognitive decline and brain atrophy in an additive way. Interestingly, our longitudinal sensitivity analyses showed that higher baseline levels of CSF YKL-40 were associated with faster atrophy in the volumes of whole brain, hippocampus, and mid temporal lobe in the A + CN rather than the A-CN subgroup. As expected, combining blood pressure profile with T status, faster atrophy in the volumes of whole brain, hippocampus, and mid temporal lobe was replicated in the T + HTN + subgroup but not in other subgroups. This is theoretically plausible given that Aβ burden in the early phases of AD is strongly associated with brain atrophy, and as the disease progresses, downstream pathological events resulting from Aβ accumulation combined with hypertension may accelerate the sustained brain atrophy (Chételat et al., 2010). Our findings highlighted the clinical application of CSF YKL-40 in combination with hypertension for monitoring and predicting the progression of AD.

The study included participants with CN, MCI and AD, had long-term follow-up within repeated tests covering multiple domains of cognitive function and brain structures, and utilized multiple analysis models, which might strengthen the plausibility and robustness of our findings. However, there were some possible limitations in this study. First, the assessment of CSF YKL-40 concentration at a single time point limited our ability to ascertain the impact of changes in YKL-40 levels on the primary AD-related pathologies, highlighting a significant area for future research endeavors. Secondly, this study only employed a simple binary classification for hypertension. A more detailed differentiation of specific blood pressure parameters, including systolic, diastolic blood pressure, pulse pressure, as well as duration, could provide a better understanding of the additive impacts of hypertension. Thirdly, the scarcity of other biomarkers for astrocyte activation (i.e., GFAP) in the ADNI database constrained our capacity to perform a more comprehensive analysis. Last, despite the exploration of potential pathways through mediation analysis, the biological overlap among these inflammatory biomarkers could impact the directional inferences made.

## Conclusion

In summary, we have observed that ICAM1, VCAM1, sTNFR1, and sTNFR2 mediate the associations between CSF YKL-40 and p-tau and t-tau. Baseline CSF YKL-40 levels adversely affected longitudinal changes in cognitive function and brain structures in the AD spectrum. Additionally, our findings highlight that hypertension has an additive effect on the mediation effects, as well as the longitudinal correlations. Taken together, this study provides convincing evidence for the development of therapeutic strategies targeting astrocyte reactivity and neuroinflammation while emphasizing the importance of controlling blood pressure to prevent AD progression.

## Data Availability

The original contributions presented in this study are included in this article/Supplementary material, further inquiries can be directed to the corresponding authors.
