# Supplementary material for: Neuroinflammation-mediated YKL-40 correlates with tau pathology and predicts longitudinal cognitive impairment and brain atrophy in Alzheimer’s disease, with hypertensive dependency
Source: Front Aging Neurosci. 2025 Aug 6;17:1630022. doi: 10.3389/fnagi.2025.1630022 (PMC12365644; doi:10.3389/fnagi.2025.1630022)
Supplement: Supplementary file 1 [file Data_Sheet_1.docx]

# Supplementary Material 1

**Content**

| **Additional files** |  |
| --- | --- |
| **Method** |  |
| Domain-specific cognitive assessment. |  |
| **TABLE** |  |
| **Table S1** Relationship of CSF YKL-40 with AD biomarkers, neuroinflammatory biomarkers, cognition and MRI brain structures. |  |
| **Table S2** Relationship of CSF neuroinflammatory biomarkers with AD biomarkers, MRI brain structures and cognition. |  |
| **Table S3** The interaction between CSF YKL-40 and vascular risk factors (Hypertension and hyperlipidemia). |  |
| **Table S4** Relationship of CSF YKL-40 with AD-related pathologies and neuroinflammatory biomarkers stratified by hypertensive status. |  |
| **Table S5** The interaction between CSF YKL-40 and variables (Aβ_42_, sex, *APOE ɛ4* status and age). |  |
| **Table S6** Mediation effects of CSF neuroinflammatory biomarkers in total participants. |  |
| **Table S7** Mediation effects of CSF neuroinflammatory biomarkers in hypertensive group. |  |
| **Table S8** Mediation effects of CSF neuroinflammatory biomarkers in normotensive group. |  |
| **Table S9** Baseline CSF YKL-40 and longitudinal changes in cognition and MRI brain structures in total participants. |  |
| **Table S10** Baseline CSF YKL-40 and longitudinal changes in cognition and MRI brain structures in hypertensive group. |  |
| **Table S11** Baseline CSF YKL-40 and longitudinal changes in cognition and MRI brain structures in normotensive group. |  |
| **FIGURE** |  |
| **Figure S1** Mediating effects of CSF neuroinflammation on the associations between YKL-40 and p-tau, t-tau and entorhinal cortex volume. |  |
| **Figure S2** Baseline CSF YKL-40 and longitudinal changes in cognition and MRI brain structures in hypertensive group. |  |
| **Figure S3** Baseline CSF YKL-40 and longitudinal changes in cognition and MRI brain structures in normotensive group. |  |

**Method**

MEM was obtained by recording some items. These items included RVLT (Trial 1, Trial 2, Trial 3, Trial 4, Trial 5, Interference, Immediate recall, 30 minutes delay and Recognition), ADAS-Cog (Trial 1, Trial2, Trial 3, Recall, Recognition present and Recognition absent), Logical Memory (Immediate and Delay) and MMSE (Ball recall, Flag recall and Tree recall).

EXF measures encompassed Category Fluency-animals, Category Fluency-vegetables, Trails A and B, Digit span backwards, WAIS-R Digit Symbol Substitution, and 5 Clock Drawing items (circle, symbol, numbers, hands, time).

LAN measures encompass relevant items of Neuropsychological Battery (Category Fluency-Animals, Category Fluency-Vegetables and Boston Naming), ADAS-Cognitive Behavior (Following Commands, Object Naming and Ideational Practice), MMSE (Naming an Object–Watch, Naming an Object–Pencil, Repeating a Sentence, Reading a Sentence, Writing a Sentence, Following a Series of Instructions) and MoCA (Letter F Fluency, Animal Naming-Lion, Camel, Rhino and Sentence Repetition).

VP was consisted by Neuropsychological Battery (Clock copy–Circle, Clock copy–Symmetry, Clock copy–Numbers, Clock copy–Hands, Clock copy–Time), ADAS-Cognitive Behavior (Constructional praxis) and MMSE (Copy design).

**TABLE**

**Table S1** Relationship of CSF YKL-40 with AD biomarkers, neuroinflammatory biomarkers, cognition and MRI brain structures.

|  | Model 1 | |  | Model 2 | |  | Model 3 | |  | Model 4 | |
| --- | --- | --- | --- | --- | --- | --- | --- | --- | --- | --- | --- |
|  | β (95% CI) | *P* value |  | β (95% CI) | *P* value |  | β (95% CI) | *P* value |  | β (95% CI) | *P* value |
| CSF AD core biomarkers | | | | | | | | |  |  |  |
| Aβ_42_ | -0.002（-0.315,0.311） | 0.991 |  | - | - |  | - | - |  | - | - |
| p-tau | 0.534（0.328,0.740） | **1.47e-6*** |  | 0.534(0.327,0.741) | **1.64e-6*** |  | 0.495(0.291,0.700) | **5.71e-6*** |  | 0.467(0.253,0.681) | **3.79e-5*** |
| T-tau | 0.507(0.328,0.686) | **1.84e-7*** |  | 0.507(0.328,0.686) | **1.95e-7*** |  | 0.474(0.297,0.651) | **7.06e-7*** |  | 0.452(0.266,0.637) | **5.56e-6*** |
| CSF neuroinflammatory biomarkers | | | | | | | | |  |  |  |
| sTNFR1 | 0.343(0.222,0.464) | **1.74e-7*** |  | 0.343(0.231,0.455) | **2.34e-8*** |  | 0.334(0.221,0.447) | **6.46e-8*** |  | 0.337(0.220,0.454) | **1.44e-7*** |
| sTNFR2 | 0.349(0.194,0.504) | **2.04e-5*** |  | 0.349(0.199,0.499) | **1.19e-5*** |  | 0.350(0.198,0.503) | **1.53e-5*** |  | 0.333(0.173,0.493) | **0.037** |
| TGF-β1 | 0.172(-0.021,0.364) | 0.080 |  | 0.172(-0.016,0.359) | 0.072 |  | 0.165(0.231,0.455) | 0.089 |  | 0.198(0.004,0.393) | **0.046** |
| IL-10 | -0.038(-0.300,0.223) | 0.772 |  | -0.038(-0.301,0.246) | 0.773 |  | -0.063(-0.328,0.203) | 0.639 |  | -0.076(-0.342,0.189) | 0.568 |
| ICAM1 | 0.319(0.046,0.593) | **0.023*** |  | 0.320(0.052,0.587) | **0.020*** |  | 0.305(0.034,0.577) | **0.028** |  | 0.314(0.033,0.595) | **0.029** |
| VCAM1 | 0.493(0.256,0.730) | **7.98e-5*** |  | 0.493(0.268,0.718) | **3.33e-5*** |  | 0.476(0.249,0.704) | **7.10e-5*** |  | 0.492(0.254,0.730) | **0.041** |
| IL-6 | -0.113(-0.449,0.223) | 0.505 |  | -0.113(-0.451,0.224) | 0.508 |  | -0.113(-0.456,0.231) | 0.517 |  | -0.198(-0.547,0.151) | 0.262 |
| IL-7 | 0.049(-0.470,0.567) | 0.853 |  | 0.048(-0.468,0.565) | 0.853 |  | -0.027(-0.543,0.489) | 0.918 |  | -0.058(-0.598,0.482) | 0.832 |
| MRI brain structures | | | | | | | | |  |  |  |
| Hippocampus | -0.067(-0.234,0.094) | 0.289 |  | -0.061(-0.172,0.050) | 0.276 |  | -0.022(-0.113,0.069) | 0.630 |  | -0.024(-0.118,0.070) | 0.613 |
| Entorhinal cortex | -0.128(-0.253,-0.002) | **0.049** |  | -0.170(-0.327,-0.013) | **0.034** |  | -0.140(-0.291,0.011) | 0.069 |  | -0.153(-0.308,0.002) | 0.053 |
| Mid temporal lobe | -0.035(-0.365,0.295) | 0.748 |  | -0.012(-0.088,0.063) | 0.750 |  | -0.002(-0.071,0.075) | 0.960 |  | -0.009(-0.084,0.065) | 0.805 |
| Whole brain | -0.0005(-0.031,-0.032) | 0.849 |  | -0.0005(-0.031,-0.032) | 0.975 |  | -0.008(-0.022,0.038) | 0.594 |  | 0.006(-0.025,0.038) | 0.694 |
| Cognitive scores | | | | | | | | |  |  |  |
| MMSE | -2.784(-5.888,0.319) | 0.078 |  | -2.781(-5.791,0.229) | 0.070 |  | -1.151(-3.296,0.994) | 0.289 |  | 0.023(-0.059,0.012) | 0.196 |
| MEM | -0.760(-1.704,0.184) | 0.113 |  | -0.759(-1.660,0.142) | 0.098 |  | -0.272(-0.917,0.372) | 0.403 |  | -0.281(-0.939,0.378) | 0.400 |
| LAN | -0.463(-1.192,0.266) | 0.210 |  | -0.462(-1.146,0.222) | 0.183 |  | -0.210(-0.819,0.399) | 0.495 |  | -0.275(-0.900,0.349) | 0.384 |
| EF | -0.222(-0.957,0.514) | 0.551 |  | -0.221(-0.949,0.507) | 0.548 |  | -0.052(-0.593,0.069) | 0.874 |  | -0.060(-0.606,0.726) | 0.859 |
| VP | 0.484(-0.139,1.107) | 0.125 |  | 0.483(-0.147,1.112) | 0.130 |  | 0.573(-0.047,1.193) | 0.069 |  | 0.574(-0.055,1.203) | 0.073 |
| FAQ | 0.488(-0.357,1.332) | 0.254 |  | 0.487(-0.347,1.321) | 0.250 |  | 1.240(-5.716,8.195) | 0.724 |  | 0.359(-6.875,7.593) | 0.922 |
| ADAS13 | 0.311(-0.046,0.669) | 0.087 |  | 0.311(-0.025,0.647) | 0.069 |  | 0.146(-0.115,0.407) | 0.269 |  | 0.165(-0.097,0.427) | 0.215 |

Model 1: adjusted by age, sex, years of education, and *APOE ɛ4* status;

Model 2: adjusted by age, sex, years of education, *APOE ɛ4* status and Aβ_42_;

Model 3: adjusted by age, sex, years of education, *APOE ɛ4* status, Aβ_42_ and diagnostic status;

Model 4: adjusted by age, sex, years of education, *APOE ɛ4* status, Aβ_42_ , diagnostic status, hypertension, DM2, lipid, stroke and smoking.

All the models were additionally adjusted intracranial volume (ICV) in the analyses of the structural MRI data.

Significance at the level of *P* < 0.05 were shown in bold.

* Significant after false discovery rate correction.

Abbreviations: CSF, cerebrospinal fluid; CI, confidence interval; Aβ, amyloid-β; p-tau, phosphorylated tau; t-tau, total tau; sTNFR, soluble tumor necrosis factor receptor; TGF, transforming growth factor; IL, interleukin; ICAM1, intercellular cell adhesion molecule-1; VCAM1, vascular cell adhesion molecule-1; MRI, Magnetic resonance imaging; MMSE, Mini-Mental State Examination; MEM, memory function; LAN, language; EF, executive function; VP, visuospatial function; FAQ, Functional Assessment Questionnaire; ADAS13, Alzheimer’s disease Assessment Scale 13.

**Table S2** Relationship of CSF neuroinflammatory biomarkers with AD biomarkers, MRI brain structures and cognition.

|  | sTNFR1 | |  | sTNFR2 | |  | TGF-β1 | |  | IL-10 | |  | ICAM1 | |  | VCAM1 | |  | IL-6 | |  | IL-7 | |
| --- | --- | --- | --- | --- | --- | --- | --- | --- | --- | --- | --- | --- | --- | --- | --- | --- | --- | --- | --- | --- | --- | --- | --- |
|  | β | *P* value |  | β | *P* value |  | β | *P* value |  | β | *P* value |  | β | *P* value |  | β | *P* value |  | β | *P* value |  | β | *P* value |
| CSF AD biomarkers | | | | | | | | | | | | | | | | | | | | | | | |
| Aβ_42_ | 0.661 | **2.66e-8*** |  | 0.466 | **5.51e-5*** |  | 0.229 | **0.004*** |  | 0.121 | 0.077 |  | 0.095 | 0.175 |  | 0.357 | **1.97e-7*** |  | 0.010 | 0.850 |  | -0.045 | 0.160 |
| p-tau | 1.010 | **<0.001*** |  | 0.964 | **<0.001*** |  | 0.363 | **1.30e-7*** |  | 0.002 | 0.975 |  | 0.364 | **1.50e-9*** |  | 0.435 | **2.20e-13*** |  | 0.039 | 0.386 |  | 0.034 | 0.220 |
| t-tau | 0.957 | **<0.001*** |  | 0.899 | **<0.001*** |  | 0.339 | **1.11e-8*** |  | -0.009 | 0.870 |  | 0.335 | **1.07e-10*** |  | 0.427 | **<0.001*** |  | 0.015 | 0.689 |  | 0.027 | 0.262 |
| MRI brain structures | | | | | | | | | | | | | | | | | | | | | | | |
| Hippocampus | -8.074e-4 | 0.986 |  | -0.039 | 0.407 |  | 0.025 | 0.427 |  | -0.009 | 0.736 |  | -0.051 | **0.046** |  | -0.024 | 0.350 |  | 8.971e-3 | 0.627 |  | -0.010 | 0.391 |
| Entorhinal cortex | 0.083 | 0.236 |  | 0.044 | 0.533 |  | 0.007 | 0.889 |  | -8.464e-3 | 0.826 |  | -0.073 | 0.063 |  | 0.043 | 0.270 |  | 7.998e-3 | 0.775 |  | -0.023 | 0.198 |
| Mid temporal lobe | 0.036 | 0.378 |  | 0.024 | 0.564 |  | -0.032 | 0.251 |  | 6.846e-4 | 0.976 |  | -9.142e-3 | 0.687 |  | -4.755e-3 | 0.833 |  | 5.760e-3 | 0.722 |  | -4.352e-3 | 0.672 |
| Whole brain | 0.016 | 0.295 |  | 0.011 | 0.441 |  | 0.014 | 0.147 |  | 9.815e-3 | 0.255 |  | -6.639e-3 | 0.455 |  | -3.494e-3 | 0.689 |  | 8.396e-3 | 0.182 |  | 6.876e-4 | 0.862 |
| Cognitive scores | | | | | | | | | | | | | | | | | | | | | | | |
| MMSE | 0.465 | 0.751 |  | -0.126 | 0.928 |  | -0.103 | 0.914 |  | -0.409 | 0.617 |  | -0.022 | 0.979 |  | -0.759 | 0.365 |  | 0.194 | 0.745 |  | -0.888 | **0.018** |
| MEM | 0.205 | 0.587 |  | -0.103 | 0.774 |  | -0.105 | 0.669 |  | -0.260 | 0.217 |  | -0.319 | 0.139 |  | -0.099 | 0.646 |  | -0.040 | 0.798 |  | -0.229 | **0.018** |
| LAN | 0.464 | 0.164 |  | 0.323 | 0.309 |  | 0.166 | 0.444 |  | 0.055 | 0.766 |  | 0.067 | 0.725 |  | 0.003 | 0.990 |  | 0.153 | 0.261 |  | -0.141 | 0.102 |
| EF | -0.300 | 0.406 |  | -0.387 | 0.261 |  | -0.151 | 0.517 |  | -0.154 | 0.446 |  | 0.034 | 0.870 |  | -0.329 | 0.111 |  | -0.052 | 0.727 |  | -0.125 | 0.180 |
| VP | 0.025 | 0.934 |  | -0.012 | 0.968 |  | 0.069 | 0.715 |  | -0.075 | 0.646 |  | 0.185 | 0.277 |  | -0.077 | 0.655 |  | 0.072 | 0.551 |  | -0.076 | 0.335 |
| FAQ | -3.447 | 0.377 |  | -0.188 | 0.960 |  | -3.874 | 0.124 |  | 1.757 | 0.419 |  | 1.214 | 0.586 |  | 0.909 | 0.684 |  | 1.803 | 0.257 |  | 1.170 | 0.244 |
| ADAS13 | -0.056 | 0.692 |  | 0.021 | 0.877 |  | 0.070 | 0.438 |  | 0.057 | 0.468 |  | 0.142 | 0.076 |  | 0.026 | 0.742 |  | -0.017 | 0.767 |  | 0.072 | **0.045** |

*P* values were obtained by multiple linear regressions models adjusted for age, sex, years of education, and apolipoprotein *E* (*APOE*) *ɛ4* status. Additionally, all the models were adjusted intracranial volume in the analyses of the structural MRI data.

Significant at the level of *P* < 0.05 were shown in bold.

* Significant after false discovery rate correction.

Abbreviations: CSF, cerebrospinal fluid; Aβ, amyloid-β; p-tau, phosphorylated tau; t-tau, total tau; sTNFR, soluble tumor necrosis factor receptor; TGF, transforming growth factor; IL, interleukin; ICAM1, intercellular cell adhesion molecule-1; VCAM1, vascular cell adhesion molecule-1; MRI, Magnetic resonance imaging; MMSE, Mini-Mental State Examination; MEM, memory function; LAN, language; EF, executive function; VP, visuospatial function; ADAS13, Alzheimer’s disease Assessment Scale 13.

**Table S3** The interaction between CSF YKL-40 and vascular risk factors (Hypertension and hyperlipidemia).

|  | CSF YKL-40 × Hypertension | |  | CSF YKL-40 × hyperlipidemia | |
| --- | --- | --- | --- | --- | --- |
|  | β (95% CI) | *P* value |  | β (95% CI) | *P* value |
| CSF AD biomarkers | | | | | |
| Aβ_42_ | -0.489(-1.094,0.116) | 0.112 |  | -0.570(-1.201,0.061) | 0.076 |
| p-tau | 0.444(0.051,0.837) | **0.027** |  | 0.397(-0.023,0.818) | 0.064 |
| T-tau | 0.347(0.005,0.689) | **0.047** |  | 0.306(-0.060,0.671) | 0.100 |
| CSF neuroinflammatory biomarkers | | | | | |
| sTNFR1 | 0.051(-0.186,0.287) | 0.672 |  | 0.051(-0.199,0.301) | 0.687 |
| sTNFR2 | 0.091(-0.210,0.391) | 0.552 |  | 0.020(-0.299,0.339) | 0.901 |
| TGF-β1 | 0.279(-0.094,0.652) | 0.141 |  | -0.135(-0.532,0.262) | 0.502 |
| IL-10 | -0.004(-0.517,0.508) | 0.987 |  | -0.096(-0.640,0.447) | 0.726 |
| ICAM1 | 0.055(-0.481,0.591) | 0.839 |  | 0.171(-0.396,0.738) | 0.551 |
| VCAM1 | -0.118(-0.583,0.347) | 0.616 |  | -0.226(-0.717,0.265) | 0.363 |
| IL-6 | -0.204(-0.449,0.857) | 0.536 |  | -0.041(-0.738,0.655) | 0.907 |
| IL-7 | 0.816(-0.188,1.819) | 0.110 |  | 0.403(-0.669,1.475) | 0.457 |
| MRI brain structures | | | | | |
| Hippocampus | -0.031(-0.185,0.124) | 0.694 |  | -0.101(-0.331,0.129) | 0.383 |
| Entorhinal | -0.136(-0.366,0.094) | 0.242 |  | -0.021(-0.365,0.322) | 0.902 |
| Mid temporal | -0.012(-0.115,0.090) | 0.812 |  | -7.994e-4(-0.151,0.153) | 0.992 |
| Whole brain | -0.012(-0.059,0.032) | 0.586 |  | -0.011(-0.072,0.052) | 0.725 |
| Cognitive scores | | | | | |
| MMSE | -0.063(-0.162,0.037) | 0.214 |  | 0.001(-0.105,0.107) | 0.981 |
| MEM | -2.089(-3.888,0.289) | **0.023** |  | -1.450(-3.389,0.489) | 0.141 |
| LAN | -0.517(-1.944,0.909) | 0.474 |  | -0.634(-2.142,0.874) | 0.406 |
| EF | -0.259(-1.690,1.172) | 0.720 |  | -0.440(-1.966,1.086) | 0.569 |
| VP | -0.491(-1.735,0.753) | 0.432 |  | -1.523(-2.856,0.189) | **0.026** |
| FAQ | 1.791(-14.717,18.300) | 0.830 |  | -7.013(-24.496,10.470) | 0.428 |
| ADAS13 | 0.811(0.130,1.491) | **0.020** |  | 0.618(-0.113,1.349) | 0.097 |

Models with interaction terms were adjusted by age, sex, years of education, apolipoprotein *E* (*APOE*) *ɛ4* status and intracranial volume (when appropriate). Significance at the level of *P* < 0.05 were shown in bold.

Abbreviations: CSF, cerebrospinal fluid; CI, confidence interval; Aβ, amyloid-β; p-tau, phosphorylated tau; t-tau, total tau; sTNFR, soluble tumor necrosis factor receptor; TGF, transforming growth factor; IL, interleukin; ICAM1, intercellular cell adhesion molecule-1; VCAM1, vascular cell adhesion molecule-1; MRI, Magnetic resonance imaging; MMSE, Mini-Mental State Examination; MEM, memory function; LAN, language; EF, executive function; VP, visuospatial function; FAQ, Functional Assessment Questionnaire; ADAS13, Alzheimer’s disease Assessment Scale 13.

**Table S4** Relationship of CSF YKL-40 with AD-related pathologies and neuroinflammatory biomarkers stratified by hypertension status.

|  | Hypertension | |  | Normotension | |
| --- | --- | --- | --- | --- | --- |
|  | β (95% CI) | *P* value |  | β (95% CI) | *P* value |
| CSF AD biomarkers | | | | | |
| Aβ_42_ | -0.235(-0.739,0.270) | 0.355 |  | 0.228(-0.174,0.629) | 0.259 |
| p-tau | 0.708(0.418,0.999) | **1.14e-5*** |  | 0.284(-0.020,0.588) | 0.066 |
| T-tau | 0.632(0.372,0.893) | **1.27e-5*** |  | 0.323(0.066,0.580) | **0.015*** |
| CSF neuroinflammatory biomarkers | | | | | |
| sTNFR1 | 0.353(0.167,0.540) | **3.98e-4*** |  | 0.299(0.101,0.432) | **0.001*** |
| sTNFR2 | 0.386(0.108,0.664) | **0.008*** |  | 0.288(0.129,0.446) | **6.69e-4*** |
| TGF-β1 | 0.289(0.032,0.546) | **0.028** |  | 0.019(-0.292,0.330) | 0.904 |
| IL-10 | 0.044(-0.339,0.427) | 0.817 |  | -0.085(-0.447,0.277) | 0.640 |
| ICAM1 | 0.307(-0.104,0.718) | 0.139 |  | 0.350(-0.029,0.730) | 0.070 |
| VCAM1 | 0.471(0.111,0.832) | **0.012*** |  | 0.513(0.166,0.859) | **0.005*** |
| IL-6 | 0.130(-0.347,0.608) | 0.586 |  | -0.417(-0.863,0.029) | 0.066 |
| IL-7 | 0.549(-0.205,1.303) | 0.149 |  | -0.460(-1.210,0.290) | 0.223 |
| MRI brain structures | | | | | |
| Hippocampus | -0.099(-0.270,0.072) | 0.247 |  | -0.042(-0.213,0.130) | 0.626 |
| Entorhinal | -0.235(-0.496,0.026) | 0.077 |  | -0.137(-0.383,0.109) | 0.268 |
| Mid temporal | -7.257e-3(-0.130,0.107) | 0.899 |  | -6.466e-3(-0.115,0.102) | 0.905 |
| Whole brain | 0.022(-0.028,0.071) | 0.378 |  | -0.029(-0.072,0.014) | 0.188 |
| Cognitive scores | | | | | |
| MMSE | -0.076(-0.144,-0.097) | **0.028** |  | -0.027(-0.110,0.057) | 0.522 |
| MEM | -1.085(-3.110,-0.499) | **0.008** |  | -0.103(-1.327,1.534) | 0.885 |
| LAN | -0.675(-1.788,0.439) | 0.229 |  | -0.320(-1.373,0.734) | 0.544 |
| EF | -0.307(-1.303,0.690) | 0.539 |  | -0.130(-1.291,1.030) | 0.822 |
| VP | 0.420(-0.496,1.336) | 0.356 |  | 1.148(0.043,2.254) | **0.042** |
| FAQ | 5.806(-7.517,19.130) | 0.385 |  | 4.216(-7.023,5.456) | 0.454 |
| ADAS13 | 0.657(0.155,1.160) | **0.012*** |  | 0.021(-0.506,0.548) | 0.937 |

P values were obtained by multiple linear regressions models adjusted for age, sex, years of education, *APOE ɛ4* status and intracranial volume (when appropriate).

Significance at the level of *P* < 0.05 were shown in bold.

* Significant after false discovery rate correction.

Abbreviations: *APOE ε4*, apolipoprotein *E4*; CSF, cerebrospinal fluid; CI, confidence interval; Aβ, amyloid-β; p-tau, phosphorylated tau; t-tau, total tau; sTNFR, soluble tumor necrosis factor receptor; TGF, transforming growth factor; IL, interleukin; ICAM1, intercellular cell adhesion molecule-1; VCAM1, vascular cell adhesion molecule-1; MRI, Magnetic resonance imaging; MMSE, Mini-Mental State Examination; MEM, memory function; LAN, language; EF, executive function; VP, visuospatial function; FAQ, Functional Assessment Questionnaire; ADAS13, Alzheimer’s disease Assessment Scale 13.

**Table S5** The interaction between CSF YKL-40 and variables (Aβ_42_, sex, *APOE ɛ4* status and age).

|  | CSF YKL-40 × Aβ_42_ | |  | CSF YKL-40 × sex | |  | CSF YKL-40 × *APOE ɛ4* status | |  | CSF YKL-40 × age | |
| --- | --- | --- | --- | --- | --- | --- | --- | --- | --- | --- | --- |
|  | β (95% CI) | *P* value |  | β (95% CI) | *P* value |  | β (95% CI) | *P* value |  | β (95% CI) | *P* value |
| CSF AD biomarkers | | | | | | | | |  |  |  |
| Aβ_42_ | - | - |  | 0.025(-0.593,0.644) | 0.935 |  | -0.280(-0.907,0.347) | 0.378 |  | -0.001(-0.047,0.044) | 0.955 |
| p-tau | -0.135（-0.542,0.272） | 0.512 |  | 0.155(-0.254,0.563) | 0.453 |  | -0.014(-0.429,0.401) | 0.948 |  | 0.023(-0.007,0.053) | 0.129 |
| T-tau | -0.115(-0.473,0.244) | 0.528 |  | 0.154(-0.199,0.507) | 0.387 |  | -0.023(-0.383,0.337) | 0.899 |  | 0.017(-0.009,0.043) | 0.202 |
| CSF neuroinflammatory biomarkers | | | | | | | | |  |  |  |
| sTNFR1 | -0.005(-0.243,0.233) | 0.966 |  | 0.068(-0.171,0.306) | 0.575 |  | -0.160(-0.401,0.081) | 0.575 |  | 0.007(-0.011,0.024) | 0.448 |
| sTNFR2 | 0.028(-0.276,0.333) | 0.854 |  | -0.027(-0.332,0.279) | 0.862 |  | -0.043(-0.353,0.268) | 0.786 |  | -0.001(-0.024,0.021) | 0.908 |
| TGF-β1 | -0.081(-0.461,0.300) | 0.675 |  | 0.177(-0.201,0.556) | 0.355 |  | -0.238(-0.622,0.147) | 0.223 |  | 0.001(-0.027,0.029) | 0.934 |
| IL-10 | 0.215(-0.310,0.741) | 0.418 |  | -0.227(-0.743,0.288) | 0.383 |  | -0.117(-0.643,0.409) | 0.660 |  | 0.005(-0.034,0.043) | 0.814 |
| ICAM1 | -0.395(-0.935,0.144) | 0.149 |  | -0.245(-0.784,0.293) | 0.368 |  | -0.338(-0.883,0.210) | 0.224 |  | 0.002(-0.034,0.042) | 0.919 |
| VCAM1 | -0.090(-0.557,0.377) | 0.702 |  | -0.003(-0.469,0.469) | 0.999 |  | -0.117(-0.594,0.360) | 0.627 |  | 0.025(-0.009,0.059) | 0.146 |
| IL-6 | 0.513(-0.155,1.182) | 0.131 |  | 0.025(-0.639,0.688) | 0.941 |  | -0.096(-0.771,0.580) | 0.779 |  | -0.005(-0.054,0.044) | 0.837 |
| IL-7 | 0.208(-0.797,1.214) | 0.682 |  | 0.728(-0.286,1.742) | 0.157 |  | 0.796(-0.234,1.826) | 0.128 |  | -0.022(-0.097,0.054) | 0.568 |
| MRI brain structures | | | | | | | | |  |  |  |
| Hippocampus | 0.107(-0.112,0.326) | 0.334 |  | -0.093(-0.316,0.130) | 0.409 |  | -0.040(-0.272,0.192) | 0.734 |  | 2.232e-6(-0.018,0.017) | 0.999 |
| Entorhinal | 0.172(-0.134,0.478) | 0.268 |  | -0.152(-0.484,0.179) | 0.364 |  | -0.001(-0.343,0.346) | 0.993 |  | 0.018(-7.800e-3,0.045) | 0.166 |
| Mid temporal | -0.013(-0.161,0.134) | 0.858 |  | -0.085(-0.232,0.061) | 0.251 |  | -0.073(-0.225,0.079) | 0.344 |  | -0.003(-0.009,0.015) | 0.593 |
| Whole brain | -0.015(-0.050,-0.079) | 0.652 |  | -0.042(-0.105,0.021) | 0.192 |  | -0.018(-0.080,0.044) | 0.565 |  | 1.939e-4(-0.005,0.005) | 0.935 |
| Cognitive scores | | | | | | | | |  |  |  |
| MMSE | -0.317(-6.245,5.610) | 0.916 |  | -0.052(-0.152,0.049) | 0.308 |  | -0.054(-0.156,0.048) | 0.296 |  | -0.007(-0.014,0.001) | 0.073 |
| MEM | -0.094(-1.898,1.710) | 0.918 |  | -0.947(-2.802,0.908) | 0.313 |  | -0.706(-2.598,1.187) | 0.461 |  | -0.062(-0.199,0.075) | 0.374 |
| LAN | -0.696(-2.071,0.679) | 0.318 |  | -0.512(-1.949,0.925) | 0.481 |  | -1.186(-2.632,0.260) | 0.107 |  | -0.054(-0.160,0.052) | 0.311 |
| EF | -0.489(-1.935,0.957) | 0.504 |  | -1.812(-3.218,-0.405) | **0.012** |  | -1.765(-3.200,-0.330) | **0.017** |  | -0.001(-0.109,0.106) | 0.979 |
| VP | -0.143(-1.704,1.418) | 0.855 |  | -0.281(-1.507,0.946) | 0.649 |  | 0.058(-1.136,1.251) | 0.923 |  | -0.017(-0.101,0.067) | 0.690 |
| FAQ | 5.242(-11.541,22.025) | 0.537 |  | 7.882(-8.726,24.489) | 0.349 |  | 9.314(-7.561,26.189) | 0.276 |  | 2.210(1.065,3.355) | **0.002** |
| ADAS13 | -0.133(-0.812,0.545) | 0.697 |  | 0.552(-0.145,1.249) | 0.119 |  | 0.048(-0.670,0.766) | 0.895 |  | 0.015(-0.037,0.067) | 0.559 |

For the model with interaction terms “CSF YKL-40 × Aβ_42_”, adjusted by age, sex, years of education, and *APOE ɛ4* status.

For the model with interaction terms “CSF YKL-40 × sex”, adjusted by age, years of education, and *APOE ɛ4* status.

For the model with interaction terms “CSF YKL-40 × *APOE ɛ4* status”, adjusted by age, sex and years of education.

For the model with interaction terms “CSF YKL-40 × age”, adjusted by sex, years of education, and *APOE ɛ4* status.

Additionally, all the models were adjusted intracranial volume in the analyses of the structural MRI data.

Significance at the level of *P* < 0.05 were shown in bold.

Abbreviations: CSF, cerebrospinal fluid; *APOE ε4*, apolipoprotein *E4*; CI, confidence interval; Aβ, amyloid-β; p-tau, phosphorylated tau; t-tau, total tau; sTNFR, soluble tumor necrosis factor receptor; TGF, transforming growth factor; IL, interleukin; ICAM1, intercellular cell adhesion molecule-1; VCAM1, vascular cell adhesion molecule-1; MRI, Magnetic resonance imaging; MMSE, Mini-Mental State Examination; MEM, memory function; LAN, language; EF, executive function; VP, visuospatial function; FAQ, Functional Assessment Questionnaire; ADAS13, Alzheimer’s disease Assessment Scale 13.

**Table S6** Mediation effects of CSF neuroinflammatory biomarkers in total participants.

|  |  |  | CSF neuroinflammatory biomarkers | | | | | | | |
| --- | --- | --- | --- | --- | --- | --- | --- | --- | --- | --- |
|  |  |  |  | ICAM1 |  | VCAM1 |  | sTNFR1 |  | sTNFR2 |
| p-tau | Direct effect | Coefficient (95%CI) |  | 0.464(0.233,0.660) |  | 0.390(0.180,0.610) |  | 0.222(0.013,0.430) |  | 0.364(0.003,0.600) |
|  |  | *P* value |  | <2e-16 |  | <2e-16 |  | 0.046 |  | 0.048 |
|  | Indirect effect | Coefficient (95%CI) |  | 0.070(0.008,0.150) |  | 0.144(0.050,0.250) |  | 0.311(0.169,0.470) |  | 0.170(0.037,0.460) |
|  |  | *P* value |  | 0.022 |  | <2e-16 |  | <2e-16 |  | <2e-16 |
|  | Total effect | Coefficient (95%CI) |  | 0.534(0.295,0.730) |  | 0.534(0.307,0.730) |  | 0.534(0.311,0.740) |  | 0.534(0.295,0.740) |
|  |  | *P* value |  | <2e-16 |  | <2e-16 |  | <2e-16 |  | <2e-16 |
|  | Proportion Mediated | Coefficient (95%CI) |  | 0.130(0.015,0.300) |  | 0.270(0.103,0.510) |  | 0.583(0.351,0.960) |  | 0.318(0.070,1.00) |
|  |  | *P* value |  | **0.022** |  | **<2e-16** |  | **<2e-16** |  | **<2e-16** |
| t-tau | Direct effect | Coefficient (95%CI) |  | 0.435(0.246,0.600) |  | 0.352(0.155,0.530) |  | 0.202(0.035,0.360) |  | 0.335(0.016,0.540) |
|  |  | *P* value |  | <2e-16 |  | <2e-16 |  | 0.024 |  | 0.038 |
|  | Indirect effect | Coefficient (95%CI) |  | 0.072(0.010,0.150) |  | 0.155(0.070,0.260) |  | 0.305(0.179,0.450) |  | 0.172(0.047,0.480) |
|  |  | *P* value |  | 0.020 |  | <2e-16 |  | <2e-16 |  | <2e-16 |
|  | Total effect | Coefficient (95%CI) |  | 0.507(0.307,0.690) |  | 0.507(0.299,0.680) |  | 0.507(0.313,0.690) |  | 0.507(0.298,0.670) |
|  |  | *P* value |  | <2e-16 |  | <2e-16 |  | <2e-16 |  | <2e-16 |
|  | Proportion Mediated | Coefficient (95%CI) |  | 0.143(0.023,0.300) |  | 0.306(0.137,0.570) |  | 0.601(0.379,0.920) |  | 0.339(0.104,0.960) |
|  |  | *P* value |  | **0.020** |  | **<2e-16** |  | **<2e-16** |  | **<2e-16** |
| Entorhinal cortex | Direct effect | Coefficient (95%CI) |  | -0.162(-0.346,0.010) |  | -0.281(-0.462,-0.110) |  | -0.295(-0.480,-0.130) |  | -0.305(-0.492,-0.120) |
|  |  | *P* value |  | 0.058 |  | 0.002 |  | <2e-16 |  | 0.002 |
|  | Indirect effect | Coefficient (95%CI) |  | -0.008(-0.055,0.030) |  | 0.110(0.028,0.210) |  | 0.125(0.047,0.220) |  | 0.134(0.030,0.250) |
|  |  | *P* value |  | 0.682 |  | 0.012 |  | <2e-16 |  | 0.012 |
|  | Total effect | Coefficient (95%CI) |  | -0.170(-0.337,-0.020) |  | -0.170(-0.331,-0.020) |  | -0.170(-0.342,-0.020) |  | -0.170(-0.331,-0.020) |
|  |  | *P* value |  | 0.030 |  | 0.024 |  | 0.030 |  | 0.024 |
|  | Proportion Mediated | Coefficient (95%CI) |  | 0.049(-0.253,0.640) |  | -0.648(-3.823,-0.100) |  | -0.734(-3.749,-0.180) |  | -0.788(-3.737,-0.090) |
|  |  | *P* value |  | 0.692 |  | **0.036** |  | **0.030** |  | **0.036** |

The mediation analyses were adjusted to age, sex, years of education, apolipoprotein *E* (*APOE*) *ɛ4* status, and additional adjustment for ICV in the analyses entorhinal cortex volume.

Significance at the level of *P* < 0.05 were shown in bold.

Abbreviations: CSF, cerebrospinal fluid; CI, confidence interval; p-tau, phosphorylated tau; t-tau, total tau; sTNFR, soluble tumor necrosis factor receptor; ICAM1, intercellular cell adhesion molecule-1; VCAM1, vascular cell adhesion molecule-1.

**Table S7** Mediation effects of CSF neuroinflammatory biomarkers in hypertensive group.

|  |  |  | CSF neuroinflammatory biomarkers | | | | | | | |
| --- | --- | --- | --- | --- | --- | --- | --- | --- | --- | --- |
|  |  |  |  | ICAM1 |  | VCAM1 |  | sTNFR1 |  | sTNFR2 |
| p-tau | Direct effect | Coefficient (95%CI) |  | 0.623(0.362,0.850) |  | 0.557(0.265,0.820) |  | 0.383(0.135,0.630) |  | 0.581(0.091,0.840) |
|  |  | *P* value |  | <2e-16 |  | <2e-16 |  | 0.006 |  | 0.010 |
|  | Indirect effect | Coefficient (95%CI) |  | 0.085(-0.016,0.200) |  | 0.151(0.014,0.340) |  | 0.326(0.109,0.530) |  | 0.127(0.007,0.580) |
|  |  | *P* value |  | 0.100 |  | 0.003 |  | 0.004 |  | 0.020 |
|  | Total effect | Coefficient (95%CI) |  | 0.708(0.405,0.960) |  | 0.708(0.410,0.950) |  | 0.708(0.384,0.940) |  | 0.708(0.423,0.930) |
|  |  | *P* value |  | <2e-16 |  | <2e-16 |  | <2e-16 |  | <2e-16 |
|  | Proportion Mediated | Coefficient (95%CI) |  | 0.120(-0.033,0.290) |  | 0.213(0.024,0.480) |  | 0.460(0.182,0.750) |  | 0.179(0.011,0.860) |
|  |  | *P* value |  | 0.100 |  | **0.030** |  | **0.004** |  | **0.020** |
| t-tau | Direct effect | Coefficient (95%CI) |  | 0.545(0.303,0.760) |  | 0.461(0.193,0.680) |  | 0.314(0.080,0.530) |  | 0.500(0.033,0.720) |
|  |  | *P* value |  | <2e-16 |  | 0.004 |  | 0.012 |  | 0.028 |
|  | Indirect effect | Coefficient (95%CI) |  | 0.088(-0.021,0.200) |  | 0.171(0.032,0.340) |  | 0.318(0.114,0.520) |  | 0.132(0.018,0.570) |
|  |  | *P* value |  | 0.090 |  | 0.016 |  | 0.002 |  | 0.006 |
|  | Total effect | Coefficient (95%CI) |  | 0.632(0.363,0.840) |  | 0.632(0.365,0.840) |  | 0.632(0.358,0.840) |  | 0.632(0.357,0.830) |
|  |  | *P* value |  | <2e-16 |  | <2e-16 |  | <2e-16 |  | <2e-16 |
|  | Proportion Mediated | Coefficient (95%CI) |  | 0.139(-0.048,0.330) |  | 0.271(0.056,0.560) |  | 0.503(0.219,0.850) |  | 0.209(0.032,0.930) |
|  |  | *P* value |  | 0.090 |  | **0.016** |  | **0.002** |  | **0.006** |

The mediation analyses were adjusted to age, sex, years of education and apolipoprotein *E* (*APOE*) *ɛ4* status.

Significance at the level of *P* < 0.05 were shown in bold.

Abbreviations: CSF, cerebrospinal fluid; CI, confidence interval; p-tau, phosphorylated tau; t-tau, total tau; sTNFR, soluble tumor necrosis factor receptor; ICAM1, intercellular cell adhesion molecule-1; VCAM1, vascular cell adhesion molecule-1.

**Table S8** Mediation effects of CSF neuroinflammatory biomarkers in normotensive group.

|  |  |  | CSF neuroinflammatory biomarkers | | | | | | | |
| --- | --- | --- | --- | --- | --- | --- | --- | --- | --- | --- |
|  |  |  |  | ICAM1 |  | VCAM1 |  | sTNFR1 |  | sTNFR2 |
| p-tau | Direct effect | Coefficient (95%CI) |  | 0.245(-0.056,0.640) |  | 0.127(-0.166,0.450) |  | 0.051(-0.290,0.340) |  | 0.010(-0.275,0.330) |
|  |  | *P* value |  | 0.116 |  | 0.396 |  | 0.850 |  | 0.970 |
|  | Indirect effect | Coefficient (95%CI) |  | 0.038(-0.071,0.190) |  | 0.157(0.021,0.280) |  | 0.233(0.066,0.510) |  | 0.274(0.081,0.540) |
|  |  | *P* value |  | 0.486 |  | 0.032 |  | 0.002 |  | 0.004 |
|  | Total effect | Coefficient (95%CI) |  | 0.284(-0.015,0.660) |  | 0.284(-0.023,0.600) |  | 0.284(-0.002,0.580) |  | 0.284(-0.020,0.630) |
|  |  | *P* value |  | 0.068 |  | 0.086 |  | 0.054 |  | 0.068 |
|  | Proportion Mediated | Coefficient (95%CI) |  | 0.135(-0.539,0.940) |  | 0.213(0.024,0.480) |  | 0.820(-0.462,5.130) |  | 0.966(-1.392,5.100) |
|  |  | *P* value |  | 0.530 |  | 0.118 |  | 0.052 |  | 0.072 |
| t-tau | Direct effect | Coefficient (95%CI) |  | 0.278(0.048,0.570) |  | 0.168(-0.048,0.450) |  | 0.086(-0.152,0.320) |  | 0.049(-0.172,0.290) |
|  |  | *P* value |  | 0.018 |  | 0.132 |  | 0.504 |  | 0.680 |
|  | Indirect effect | Coefficient (95%CI) |  | 0.045(-0.040,0.170) |  | 0.155(0.038,0.290) |  | 0.237(0.076,0.460) |  | 0.275(0.009,0.540) |
|  |  | *P* value |  | 0.380 |  | 0.008 |  | <2e-16 |  | <2e-16 |
|  | Total effect | Coefficient (95%CI) |  | 0.323(0.080,0.580) |  | 0.323(0.091,0.610) |  | 0.323(0.107,0.580) |  | 0.323(0.086,0.590) |
|  |  | *P* value |  | 0.006 |  | 0.006 |  | 0.004 |  | 0.004 |
|  | Proportion Mediated | Coefficient (95%CI) |  | 0.140(-0.150,0.590) |  | 0.480(0.097,1.500) |  | 0.734(0.2691.980) |  | 0.849(0.361,2.180) |
|  |  | *P* value |  | 0.382 |  | **0.010** |  | **0.004** |  | **0.004** |

The mediation analyses were adjusted to age, sex, years of education and apolipoprotein *E* (*APOE*) *ɛ4* status.

Significance at the level of *P* < 0.05 were shown in bold.

Abbreviations: CSF, cerebrospinal fluid; CI, confidence interval; p-tau, phosphorylated tau; t-tau, total tau; sTNFR, soluble tumor necrosis factor receptor; ICAM1, intercellular cell adhesion molecule-1; VCAM1, vascular cell adhesion molecule-1.

**Table S9** Baseline CSF YKL-40 and longitudinal changes in cognition and MRI brain structures in total participants.

|  |  | MMSE | | | | |  | FAQ | | | | |  | ADAS13 | | | | |  | \ | | | | |
| --- | --- | --- | --- | --- | --- | --- | --- | --- | --- | --- | --- | --- | --- | --- | --- | --- | --- | --- | --- | --- | --- | --- | --- | --- |
| Predictors |  | Coefficients |  | SE |  | *P* value |  | Coefficients |  | SE |  | *P* value |  | Coefficients |  | SE |  | *P* value |  | Coefficients |  | SE |  | *P* value |
| Age × time |  | -0.005 |  | 0.005 |  | 0.358 |  | -0.024 |  | 0.009 |  | 0.005 |  | -0.015 |  | 0.014 |  | 0.272 |  | \ |  | \ |  | \ |
| Female × time |  | 0.259 |  | 0.067 |  | 1.110e-4 |  | -0.137 |  | 0.118 |  | 0.241 |  | -0.499 |  | 0.178 |  | 0.005 |  | \ |  | \ |  | \ |
| Education × time |  | -0.019 |  | 0.010 |  | 0.056 |  | 0.014 |  | 0.017 |  | 0.433 |  | 0.022 |  | 0.026 |  | 0.397 |  | \ |  | \ |  | \ |
| *APOE ɛ4* × time |  | -0.665 |  | 0.067 |  | <2e-16 |  | 1.598 |  | 0.117 |  | <2e-16 |  | 1.517 |  | 0.174 |  | <2e-16 |  | \ |  | \ |  | \ |
| Group high × time |  | -0.185 |  | 0.067 |  | **0.006** |  | 0.240 |  | 0.117 |  | **0.042** |  | 0.177 |  | 0.177 |  | 0.318 |  |  |  |  |  |  |
|  |  | MEM | | | | |  | LAN | | | | |  | EF | | | | |  | VP | | | | |
| Predictors |  | Coefficients |  | SE |  | *P* value |  | Coefficients |  | SE |  | *P* value |  | Coefficients |  | SE |  | *P* value |  | Coefficients |  | SE |  | *P* value |
| Age × time |  | -8.420e-4 |  | 6.464e-4 |  | 0.193 |  | 1.166e-3 |  | 7.264e-4 |  | 0.109 |  | 1.182e-3 |  | 7.993e-4 |  | 0.140 |  | 2.985e-3 |  | 1.390e-3 |  | 0.026 |
| Female × time |  | 0.037 |  | 8.567e-3 |  | 1.460e-5 |  | 0.044 |  | 9.627e-3 |  | 5.910e-6 |  | 0.059 |  | 0.011 |  | 3.240e-8 |  | 0.014 |  | 0.017 |  | 0.404 |
| Education × time |  | -1.819e-3 |  | 1.289e-3 |  | 0.159 |  | -0.004 |  | 1.449e-3 |  | 0.016 |  | -5.729e-3 |  | 1.588e-3 |  | 3.310e-4 |  | -3.404e-3 |  | 2.379e-3 |  | 0.153 |
| *APOE ɛ4* × time |  | -8.330e-2 |  | 8.278e-3 |  | <2e-16 |  | -0.066 |  | 9.301e-3 |  | 2.36e-12 |  | -8.151e-2 |  | 0.010 |  | 6.78e-15 |  | -0.049 |  | 0.016 |  | 2.395e-3 |
| Group high × time |  | -3.887e-2 |  | 8.364e-3 |  | **4.210e-6** |  | -0.025 |  | 9.403e-3 |  | **0.007** |  | -8.646e-3 |  | 0.010 |  | 0.408 |  | -0.068 |  | 0.018 |  | **1.570e-4** |
|  |  | Hippocampus | | | | |  | Entorhinal cortex | | | | |  | Mid temporal | | | | |  | Whole brain | | | | |
| Predictors |  | Coefficients |  | SE |  | *P* value |  | Coefficients |  | SE |  | *P* value |  | Coefficients |  | SE |  | *P* value |  | Coefficients |  | SE |  | *P* value |
| Age × time |  | -6.785e-5 |  | 7.566e-5 |  | 0.370 |  | 8.448e-5 |  | 7.543e-5 |  | 0.263 |  | 1.737e-4 |  | 6.146e-5 |  | 0.005 |  | 8.531e-5 |  | 2.625e-5 |  | 0.002 |
| Female × time |  | 1.224e-3 |  | 1.158e-3 |  | 0.291 |  | 3.396e-3 |  | 1.115e-3 |  | 0.003 |  | 1.884e-3 |  | 4.409e-4 |  | 0.046 |  | 7.950e-4 |  | 4.018e-4 |  | 0.049 |
| Education × time |  | 1.110e-4 |  | 1.800e-4 |  | 0.538 |  | -2.030e-4 |  | 1.794e-4 |  | 0.257 |  | -1.109e-4 |  | 1.462e-4 |  | 0.448 |  | 1.910e-5 |  | 6.244e-5 |  | 0.760 |
| *APOE ɛ4* × time |  | -4.954e-3 |  | 9.629e-4 |  | 4.380e-7 |  | -7.179e-3 |  | 9.601e-4 |  | 5.64e-13 |  | -4.860e-3 |  | -7.82e-4 |  | 1.420e-9 |  | -1.448e-3 |  | 3.341e-4 |  | 1.890e-5 |
| ICV × time |  | 5.294e-10 |  | 4.542e-9 |  | 0.907 |  | 4.898e-9 |  | 4.528e-9 |  | 0.280 |  | 4.351e-9 |  | 3.689e-9 |  | 0.239 |  | 1.044e-10 |  | 1.576e-9 |  | 0.947 |
| Group high × time |  | -2.880e-3 |  | **9.056e-4** |  | 0.002 |  | -2.865e-3 |  | 9.030e-4 |  | **0.002** |  | -2.196e-3 |  | 7.357e-4 |  | **0.003** |  | -6.928e-4 |  | 3.142e-4 |  | **0.028** |

The primary effects of the predictive factors (i.e., age, sex, years of education, *APOE ɛ4* status, years since baseline, and ICV when appropriate) were incorporated into all linear mixed-effects models, while coefficients are not shown for the sake of brevity.

Abbreviations: MMSE, Mini-Mental State Examination; FAQ, Functional Assessment Questionnaire; ADAS13, Alzheimer’s disease Assessment Scale 13; MEM, memory function; LAN, language; EF, executive function; VP, visuospatial function; *APOE ε4*, apolipoprotein *E4*; ICV, Intracranial volume; SE, Standard Error; Group high, CSF YKL-40 ≥ 390ng/ml (determined by the median concentration).

**Table S10** Baseline CSF YKL-40 and longitudinal changes in cognition and MRI brain structures in hypertensive group.

|  |  | MMSE | | | | |  | FAQ | | | | |  | ADAS13 | | | | |  | \ | | | | |
| --- | --- | --- | --- | --- | --- | --- | --- | --- | --- | --- | --- | --- | --- | --- | --- | --- | --- | --- | --- | --- | --- | --- | --- | --- |
| Predictors |  | Coefficients |  | SE |  | *P* value |  | Coefficients |  | SE |  | *P* value |  | Coefficients |  | SE |  | *P* value |  | Coefficients |  | SE |  | *P* value |
| Age × time |  | -0.026 |  | 0.006 |  | 1.22e-5 |  | 0.063 |  | 0.009 |  | 1.05e-10 |  | 0.022 |  | 0.016 |  | 0.175 |  | \ |  | \ |  | \ |
| Female × time |  | 0.402 |  | 0.099 |  | 5.79e-4 |  | -0.483 |  | 0.160 |  | 2.81e-3 |  | -1.274 |  | 0.271 |  | 3.49e-6 |  | \ |  | \ |  | \ |
| Education × time |  | -0.014 |  | 0.016 |  | 0.366 |  | -0.034 |  | 0.025 |  | 0.186 |  | 0.085 |  | 0.041 |  | 0.042 |  | \ |  | \ |  | \ |
| *APOE ɛ4* × time |  | -0.563 |  | 0.099 |  | 2.60e-8 |  | 1.359 |  | 0.159 |  | 3.19e-16 |  | 1.761 |  | 0.247 |  | 5.17e-12 |  | \ |  | \ |  | \ |
| Group high × time |  | -0.305 |  | 0.088 |  | **5.83e-4** |  | 0.631 |  | 0.143 |  | **1.28e-5** |  | 0.521 |  | 0.224 |  | **0.021** |  |  |  |  |  |  |
|  |  | MEM | | | | |  | LAN | | | | |  | EF | | | | |  | VP | | | | |
| Predictors |  | Coefficients |  | SE |  | *P* value |  | Coefficients |  | SE |  | *P* value |  | Coefficients |  | SE |  | *P* value |  | Coefficients |  | SE |  | *P* value |
| Age × time |  | -3.88e-3 |  | 8.266e-4 |  | 3.40e-6 |  | -1.44e-3 |  | 9.21e-4 |  | 0.120 |  | 8.27e-4 |  | 9.84e-4 |  | 0.401 |  | 2.96e-3 |  | 1.85e-3 |  | 0.112 |
| Female × time |  | 0.095 |  | 0.014 |  | 2.70e-11 |  | 0.058 |  | 0.015 |  | 1.66e-4 |  | 0.083 |  | 0.017 |  | 8.90e-7 |  | 7.64e-3 |  | 3.13e-3 |  | 0.807 |
| Education × time |  | -1.05e-3 |  | 2.003e-3 |  | 0.600 |  | -6.16e-3 |  | 2.243e-3 |  | 6.31e-3 |  | -0.010 |  | 2.40e-3 |  | 2.49e-5 |  | -5.23e-3 |  | 4.05e-3 |  | 0.198 |
| *APOE ɛ4* × time |  | -0.099 |  | 0.013 |  | 7.39e-14 |  | -0.076 |  | 0.014 |  | 1.94e-7 |  | -0.092 |  | 0.015 |  | 5.22e-9 |  | -0.060 |  | 0.025 |  | 0.019 |
| Group high × time |  | -0.062 |  | 0.011 |  | **1.04e-17** |  | -0.027 |  | 0.013 |  | **0.033** |  | -0.021 |  | 0.014 |  | 0.120 |  | -0.048 |  | 0.024 |  | **0.047** |
|  |  | Hippocampus | | | | |  | Entorhinal cortex | | | | |  | Mid temporal lobe | | | | |  | Whole brain | | | | |
| Predictors |  | Coefficients |  | SE |  | *P* value |  | Coefficients |  | SE |  | *P* value |  | Coefficients |  | SE |  | *P* value |  | Coefficients |  | SE |  | *P* value |
| Age × time |  | -4.15e-4 |  | 9.18e-5 |  | 1.12e-5 |  | -3.48e-5 |  | 9.18e-5 |  | 0.705 |  | 1.30e-4 |  | 7.78e-5 |  | 0.095 |  | 3.95e-5 |  | 3.11e-5 |  | 0.205 |
| Female × time |  | 3.77e-3 |  | 1.60e-3 |  | 0.019 |  | 4.96e-3 |  | 1.60e-3 |  | 0.002 |  | 1.45e-3 |  | 1.36e-3 |  | 0.286 |  | 9.93e-4 |  | 5.42e-4 |  | 0.068 |
| Education × time |  | 7.19e-5 |  | 2.25e-4 |  | 0.750 |  | -4.12e-4 |  | 2.25e-4 |  | 0.069 |  | -4.26e-4 |  | 1.91e-4 |  | 0.027 |  | -9.86e-5 |  | 7.61e-5 |  | 0.197 |
| *APOE ɛ4* × time |  | -5.34e-3 |  | 1.26e-3 |  | 3.77e-5 |  | -7.29e-3 |  | 1.26e-3 |  | 3.42e-8 |  | -3.35e-3 |  | 1.07e-3 |  | 2.05e-3 |  | -8.40e-4 |  | 4.28e-4 |  | 0.051 |
| ICV × time |  | -1.28e-8 |  | 6.15e-9 |  | 0.039 |  | 1.60e-9 |  | 6.15e-9 |  | 0.795 |  | 4.33e-9 |  | 5.21e-9 |  | 0.407 |  | --7.31e-10 |  | 2.08e-9 |  | 0.726 |
| Group high × time |  | -4.21e-3 |  | 1.19e-3 |  | **5.31e-4** |  | -4.97e-3 |  | 1.20e-3 |  | **4.86e-5** |  | -3.83e-3 |  | 1.01e-3 |  | **2.14e-4** |  | -1.32e-3 |  | 4.04e-4 |  | **1.30e-3** |

The primary effects of the predictive factors (i.e., age, sex, years of education, *APOE ɛ4* status, years since baseline, and ICV when appropriate) were incorporated into all linear mixed-effects models, while coefficients are not shown for the sake of brevity.

Abbreviations: MMSE, Mini-Mental State Examination; FAQ, Functional Assessment Questionnaire; ADAS13, Alzheimer’s disease Assessment Scale 13; MEM, memory function; LAN, language; EF, executive function; VP, visuospatial function; *APOE ε4*, apolipoprotein *E4*; ICV, Intracranial volume; SE, Standard Error; Group High, CSF YKL-40 ≥ 390ng/ml (determined by the median concentration).

**Table S11** Baseline CSF YKL-40 and longitudinal changes in cognition and MRI brain structures in normotensive group.

|  |  | MMSE | | | | |  | FAQ | | | | |  | ADAS13 | | | | |  | \ | | | | |
| --- | --- | --- | --- | --- | --- | --- | --- | --- | --- | --- | --- | --- | --- | --- | --- | --- | --- | --- | --- | --- | --- | --- | --- | --- |
| Predictors |  | Coefficients |  | SE |  | *P* value |  | Coefficients |  | SE |  | *P* value |  | Coefficients |  | SE |  | *P* value |  | Coefficients |  | SE |  | *P* value |
| Age × time |  | 0.044 |  | 0.010 |  | 8.25e-6 |  | -0.044 |  | 0.017 |  | 8.85e-4 |  | -0.063 |  | 0.027 |  | 0.019 |  | \ |  | \ |  | \ |
| Female × time |  | 0.181 |  | 0.098 |  | 0.064 |  | 0.039 |  | 0.168 |  | 0.817 |  | -0.152 |  | 0.267 |  | 0.570 |  | \ |  | \ |  | \ |
| Education × time |  | -0.013 |  | 0.014 |  | 0.337 |  | 0.069 |  | 0.024 |  | 0.004 |  | 0.013 |  | 0.039 |  | 0.738 |  | \ |  | \ |  | \ |
| *APOE ɛ4* × time |  | -0.567 |  | 0.104 |  | 9.25e-8 |  | 1.445 |  | 0.180 |  | 1.13e-14 |  | 1.389 |  | 0.292 |  | 2.81e-6 |  | \ |  | \ |  | \ |
| Group high × time |  | -0.209 |  | 0.101 |  | **0.039** |  | -0.028 |  | 0.174 |  | 0.873 |  | -5.65e-3 |  | 0.290 |  | 0.984 |  |  |  |  |  |  |
|  |  | MEM | | | | |  | LAN | | | | |  | EF | | | | |  | VP | | | | |
| Predictors |  | Coefficients |  | SE |  | *P* value |  | Coefficients |  | SE |  | *P* value |  | Coefficients |  | SE |  | *P* value |  | Coefficients |  | SE |  | *P* value |
| Age × time |  | 1.67e-3 |  | 1.01e-3 |  | 0.098 |  | 7.02e-3 |  | 1.27e-3 |  | 5.95e-8 |  | 5.61e-3 |  | 0.001 |  | 1.55e-4 |  | 5.30e-3 |  | 2.30e-3 |  | 0.022 |
| Female × time |  | 2.74e-3 |  | 0.011 |  | 0.799 |  | 0.052 |  | 0.014 |  | 1.45e-4 |  | 0.060 |  | 0.015 |  | 1.13e-4 |  | 0.025 |  | 0.023 |  | 0.270 |
| Education × time |  | -4.99e-3 |  | 1.644e-3 |  | 2.59e-3 |  | -4.87e-4 |  | 2.06e-3 |  | 0.814 |  | -2.65e-3 |  | 0.002 |  | 0.259 |  | -2.47e-3 |  | 3.28e-3 |  | 0.452 |
| *APOE ɛ4* × time |  | -0.085 |  | 0.012 |  | 1.77e-12 |  | -0.035 |  | 0.015 |  | 0.017 |  | -0.066 |  | 0.016 |  | 6.94e-5 |  | -0.026 |  | 0.026 |  | 0.311 |
| Group high × time |  | -0.022 |  | 0.011 |  | 0.052 |  | -0.039 |  | 0.014 |  | **0.005** |  | -0.011 |  | 0.016 |  | 0.503 |  | -0.109 |  | 0.029 |  | **2.65e-4** |
|  |  | Hippocampus | | | | |  | Entorhinal cortex | | | | |  | Mid temporal lobe | | | | |  | Whole brain | | | | |
| Predictors |  | Coefficients |  | SE |  | *P* value |  | Coefficients |  | SE |  | *P* value |  | Coefficients |  | SE |  | *P* value |  | Coefficients |  | SE |  | *P* value |
| Age × time |  | 3.27e-4 |  | 1.21e-4 |  | 7.65e-3 |  | 2.42e-4 |  | 1.26e-4 |  | 0.090 |  | 2.39e-4 |  | 9.89e-5 |  | 0.016 |  | 1.42e-4 |  | 4.45e-5 |  | 0.002 |
| Female × time |  | 4.06e-4 |  | 1.61e-3 |  | 0.801 |  | 2.29e-3 |  | 1.68e-3 |  | 0.174 |  | 2.59e-3 |  | 1.32e-3 |  | 0.050 |  | 8.51e-4 |  | 5.92e-4 |  | 0.152 |
| Education × time |  | 2.07e-4 |  | 2.99e-4 |  | 0.489 |  | 1.24e-4 |  | 3.11e-4 |  | 0.691 |  | 1.77e-4 |  | 2.44e-4 |  | 0.469 |  | 1.38e-4 |  | 1.10e-4 |  | 0.210 |
| *APOE ɛ4* × time |  | -3.43e-3 |  | 1.43e-3 |  | 0.017 |  | -5.86e-3 |  | 1.49e-3 |  | 1.18e-4 |  | -5.52e-3 |  | 1.67e-3 |  | 4.56e-6 |  | -1.64e-3 |  | 5.26e-4 |  | 0.002 |
| ICV × time |  | 6.58e-9 |  | 6.57e-9 |  | 0.318 |  | 4.33e-9 |  | 6.84e-9 |  | 0.527 |  | 3.44e-9 |  | 5.36e-9 |  | 0.521 |  | --2.27e-10 |  | 2.41e-9 |  | 0.925 |
| Group high × time |  | -2.611e-3 |  | 1.37e-3 |  | 0.059 |  | -1.20e-3 |  | 1.43e-3 |  | 0.403 |  | -1.69e-3 |  | 1.12e-3 |  | 0.134 |  | -6.49e-4 |  | 5.04e-4 |  | 0.200 |

The primary effects of the predictive factors (i.e., age, sex, years of education, *APOE ɛ4* status, years since baseline, and ICV when appropriate) were incorporated into all linear mixed-effects models, while coefficients are not shown for the sake of brevity.

Abbreviations: MMSE, Mini-Mental State Examination; FAQ, Functional Assessment Questionnaire; ADAS13, Alzheimer’s disease Assessment Scale 13; MEM, memory function; LAN, language; EF, executive function; VP, visuospatial function; *APOE ε4*, apolipoprotein *E4*; ICV, Intracranial volume; SE, Standard Error; Group High, CSF YKL-40 ≥ 390ng/ml (determined by the median concentration).

**FIGURE**

**Figure S1** Mediation effects of CSF neuroinflammation on the associations between YKL-40 and p-tau, t-tau and entorhinal cortex volume.


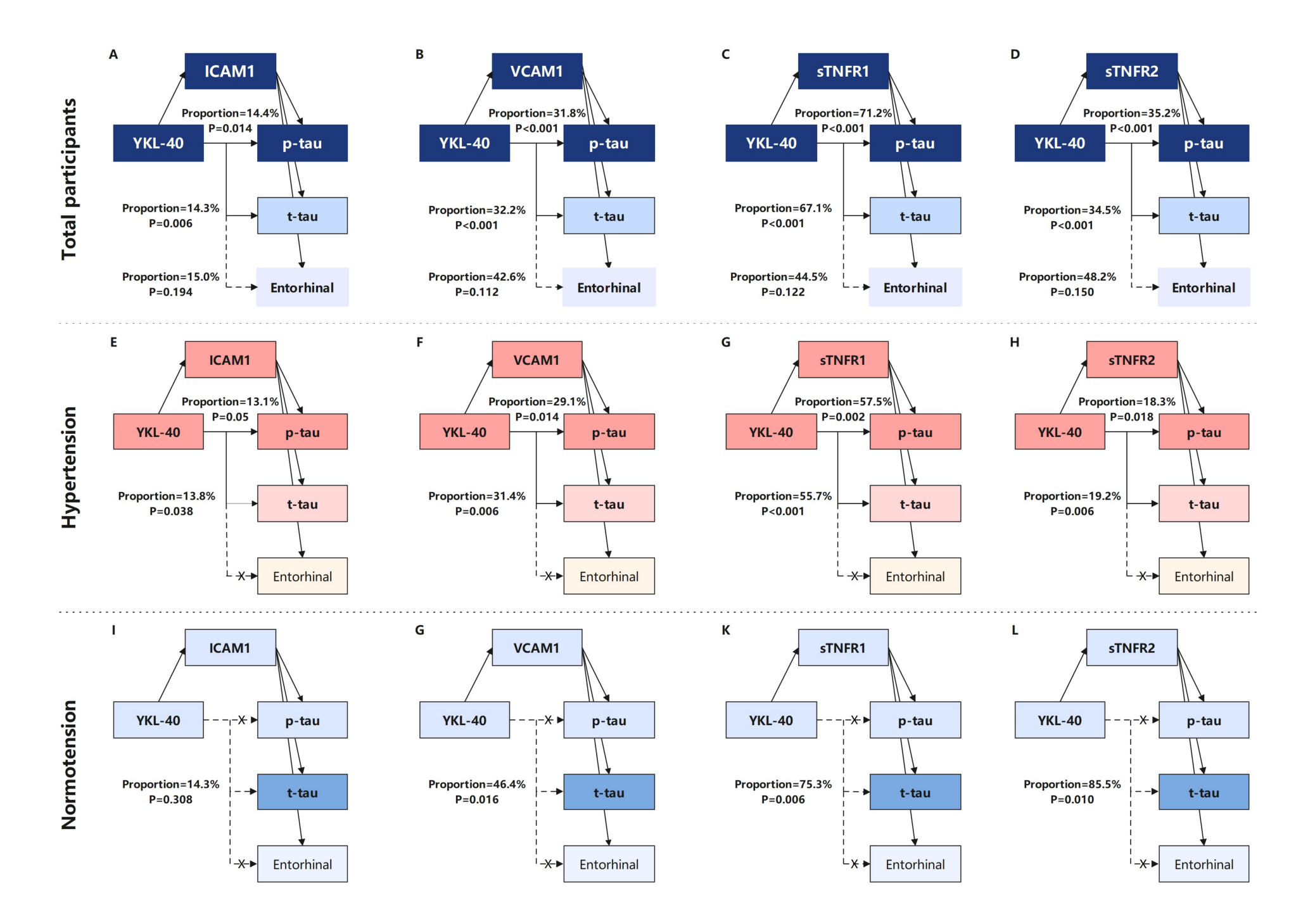


The "x" signified the absence of a significant correlation with CSF YKL-40, and as such, no additional mediation analysis was performed. The dotted line indicated that the direct effect is not significant (*P* ≥ 0.05). Mediation effects were shown in the whole sample (**A** to **D**), hypertensive (**E** to **H**) and normotensive (**I** to **L**) groups, respectively. The proportions shown in the figure indicate the proportion of mediating factors in the total effect of CSF YKL-40 on AD-related pathologies. Mediation analyses with 10,000 bootstrapped iterations were used to examine the mediation effects of CSF neuroinflammatory biomarkers on p-tau, t-tau, and entorhinal cortex volume. Each path of the model was adjusted for age, sex, years of education, *APOE ɛ4* status, Aβ_42_ and ICV (when appropriate). Abbreviations: CSF, cerebrospinal fluid; p-tau, phosphorylated tau; t-tau, total tau; ICAM1, intercellular cell adhesion molecule-1; VCAM1, vascular cell adhesion molecule-1; sTNFR, soluble tumor necrosis factor receptor.

**Figure S2** Baseline CSF YKL-40 and longitudinal changes in cognition and MRI brain structures in hypertensive group.


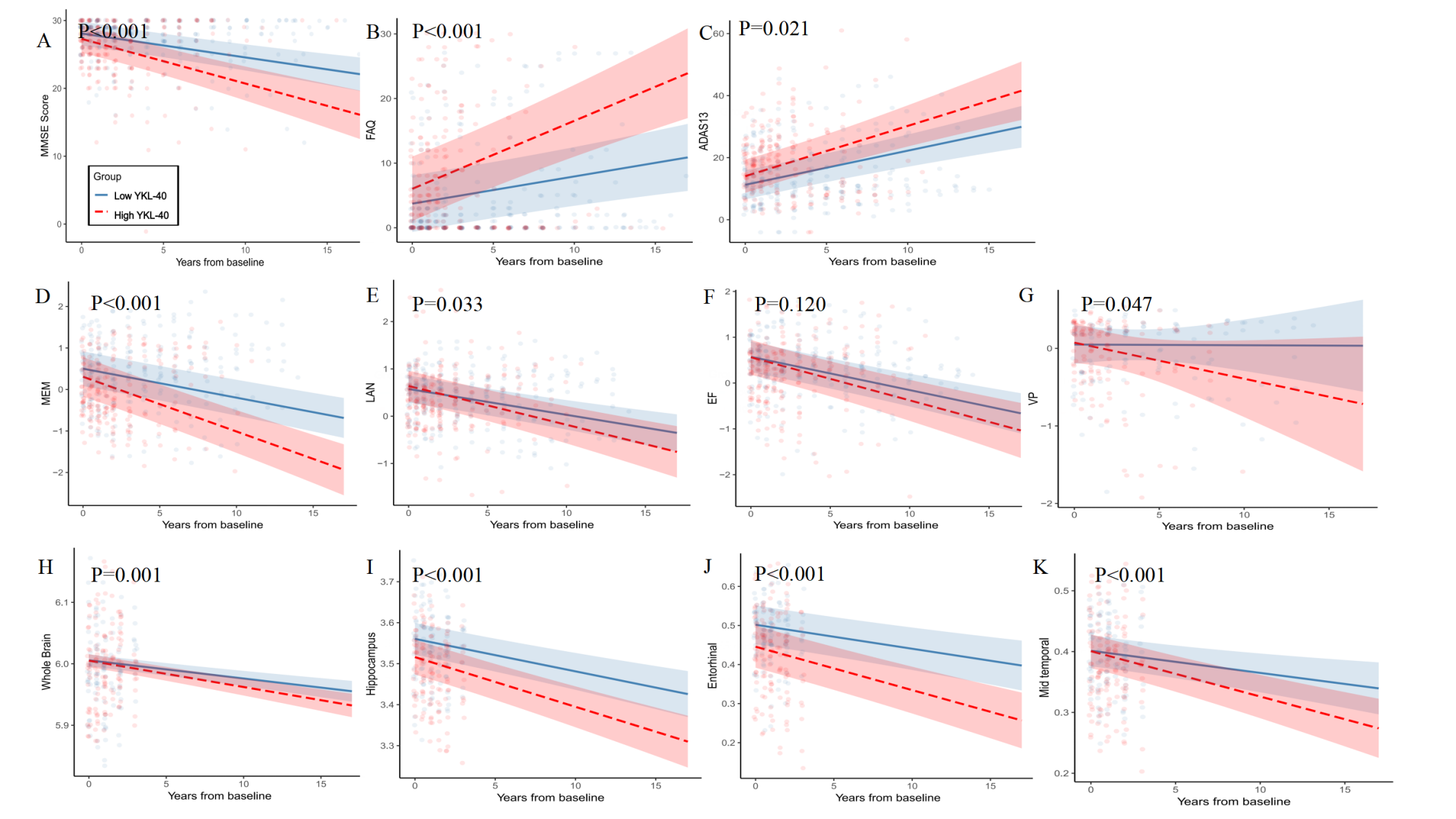


The mixed-effect models were fit using continuous CSF YKL-40 values, adjusting for age, sex, years of education, *APOE ɛ4* status and ICV (when appropriate). For better illustration, the plots displayed the trajectories for individuals with high and low CSF YKL-40 (≥390ng/ml VS <390ng/ml) for longitudinal cognition as well as for MRI brain structures. We found protective roles of low levels of CSF YKL-40 in preventing decline of cognitive functions, including MMSE (**A**), FAQ (**B**), ADAS13 (**C**), MEM (**D**), LAN (**E**), VP (**G**), as well as MRI brain structures, including whole brain volume (**H**), hippocampus volume (**I**), Entorhinal cortex volume (**J**) and mid temporal lobe (**K**) in hypertensive group. Abbreviations: CSF, cerebrospinal fluid; MMSE, Mini-Mental State Examination; FAQ, Functional Assessment Questionnaire; ADAS13, Alzheimer’s disease Assessment Scale 13; MEM, memory function; LAN, language; EF, executive function; VP, visuospatial function.

**Figure S3** Baseline CSF YKL-40 and longitudinal changes in cognition and MRI brain structures in normotensive group.


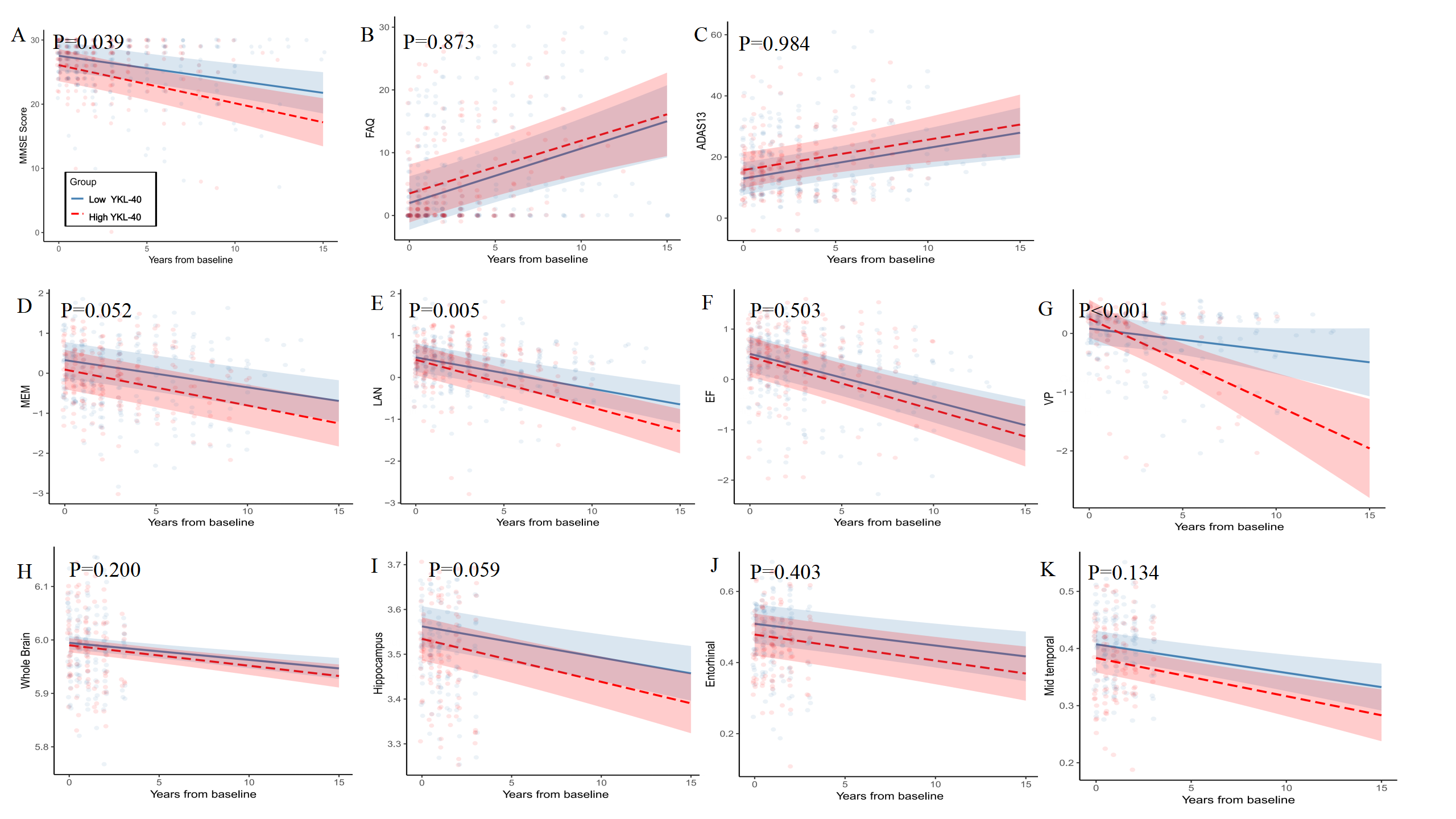

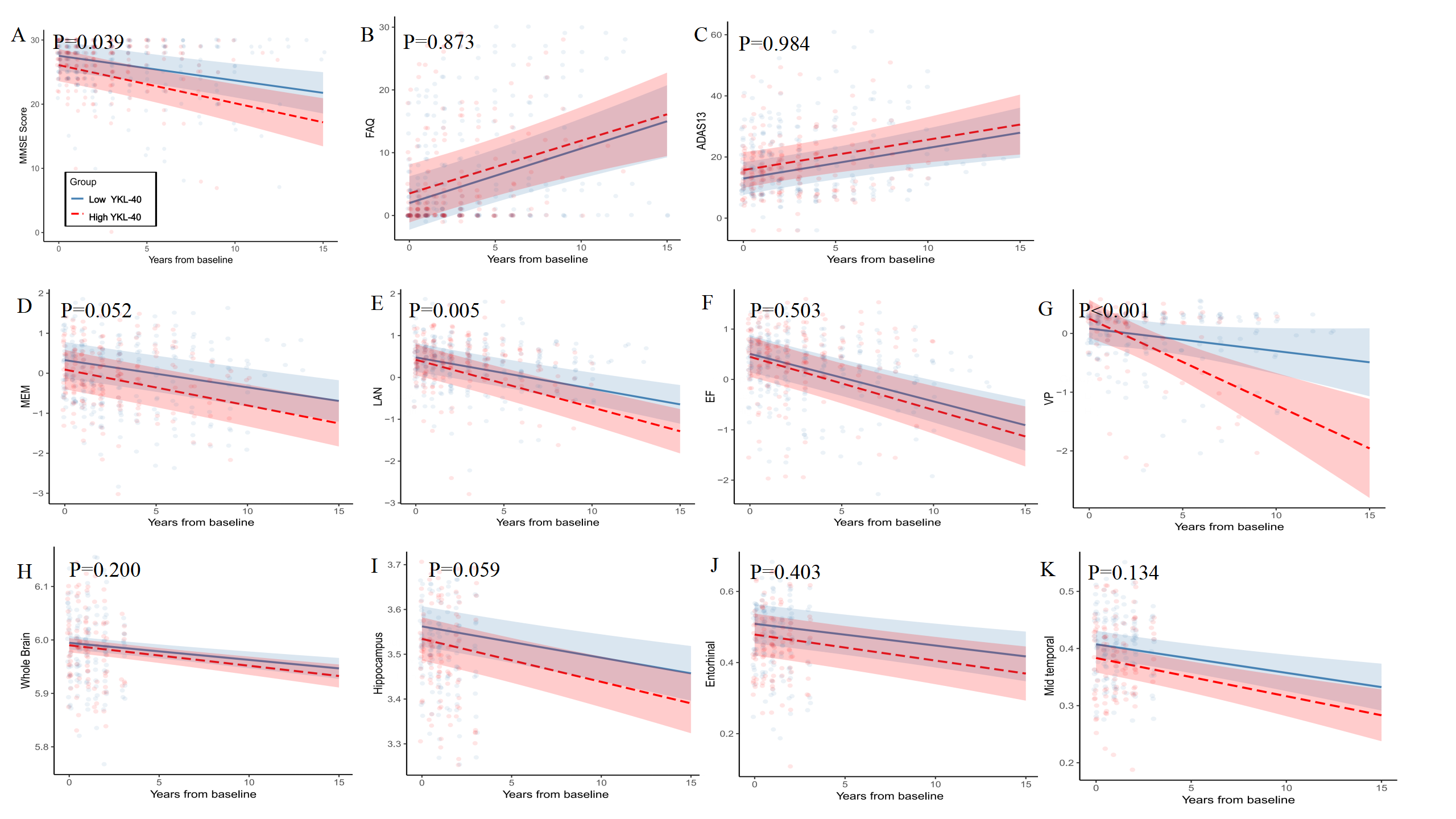


The mixed-effect models were fit using continuous CSF YKL-40 values, adjusting for age, sex, years of education, *APOE ɛ4* status and ICV (when appropriate). For better illustration, the plots displayed the trajectories for individuals with high and low CSF YKL-40 (≥390ng/ml VS <390ng/ml) for longitudinal cognition as well as for MRI brain structures. We found protective roles of low levels of CSF YKL-40 in preventing decline of cognitive functions, including MMSE (**A**), LAN (**E**), and VP (**G**) in normotensive group. Abbreviations: CSF, cerebrospinal fluid; MMSE, Mini-Mental State Examination; FAQ, Functional Assessment Questionnaire; ADAS13, Alzheimer’s disease Assessment Scale 13; MEM, memory function; LAN, language; EF, executive function; VP, visuospatial function.
