# Supplementary material for: Neuroinflammation-mediated YKL-40 correlates with tau pathology and predicts longitudinal cognitive impairment and brain atrophy in Alzheimer’s disease, with hypertensive dependency
Source: Front Aging Neurosci. 2025 Aug 6;17:1630022. doi: 10.3389/fnagi.2025.1630022 (PMC12365644; doi:10.3389/fnagi.2025.1630022)
Supplement: Supplementary file 2 [file Data_Sheet_2.docx]

# Supplementary Material 2

**Content**

| **Additional files** |  |
| --- | --- |
| **Table S1** Relationship of CSF YKL-40 with AD-related pathologies and neuroinflammatory biomarkers stratified by clinical diagnosis. |  |
| **Table S2** Relationship of CSF YKL-40 with AD-related pathologies and neuroinflammatory biomarkers stratified by T status. |  |
| **Table S3** Relationship of CSF YKL-40 with AD-related pathologies and neuroinflammatory biomarkers stratified by T status and hypertensive status. |  |
| **Table S4** Relationship of CSF YKL-40 with AD-related pathologies and neuroinflammatory biomarkers stratified by A status. |  |
| **Table S5** Relationship of CSF YKL-40 with AD-related pathologies and neuroinflammatory biomarkers stratified by A status and hypertensive status. |  |
| **Table S6** Relationship of CSF YKL-40 with AD-related pathologies and neuroinflammatory biomarkers stratified by A status and clinical diagnosis. |  |
| **Table S7** Mediation effects of CSF neuroinflammatory biomarkers stratified by clinical diagnosis. |  |
| **Table S8** Mediation effects of CSF neuroinflammatory biomarkers stratified by T status. |  |
| **Table S9** Mediation effects of CSF neuroinflammatory biomarkers stratified by T status and hypertensive status. |  |
| **Table S10** Mediation effects of CSF neuroinflammatory biomarkers stratified by A status. |  |
| **Table S11** Mediation effects of CSF neuroinflammatory biomarkers stratified by A status and hypertensive status. |  |
| **Table S12** Mediation effects of CSF neuroinflammatory biomarkers stratified by A status and clinical diagnosis. |  |
| **Table S13** Baseline CSF YKL-40 and longitudinal changes in cognition and MRI brain structures stratified by clinical diagnosis. |  |
| **Table S14** Baseline CSF YKL-40 and longitudinal changes in cognition and MRI brain structures stratified by T status. |  |
| **Table S15** Baseline CSF YKL-40 and longitudinal changes in cognition and MRI brain structures stratified by T status and hypertensive status. |  |
| **Table S16** Baseline CSF YKL-40 and longitudinal changes in cognition and MRI brain structures stratified by A status. |  |
| **Table S17** Baseline CSF YKL-40 and longitudinal changes in cognition and MRI brain structures stratified by A status and hypertensive status. |  |
| **Table S18** Baseline CSF YKL-40 and longitudinal changes in cognition and MRI brain structures stratified by A status and clinical diagnosis. |  |

**Table S1** Relationship of CSF YKL-40 with AD-related pathologies and neuroinflammatory biomarkers stratified by clinical diagnosis.

|  | CN  (N=84) | |  | MCI  (N=127) | |  | AD  (N=77) | |
| --- | --- | --- | --- | --- | --- | --- | --- | --- |
|  | β (95% CI) | *P* value |  | β (95% CI) | *P* value |  | β (95% CI) | *P* value |
| CSF AD biomarkers | | | | | |  |  |  |
| Aβ_42_ | -0.009(-0.532,0.514) | 0.973 |  | 0.085(-0.381,0.051) | 0.716 |  | 0.446(-0.414,1.306) | 0.266 |
| p-tau | 0.290(-0.030,0.610) | 0.075 |  | 0.524(0.231,0.816) | **7.99e-4*** |  | 0.740(-0.134,1.615) | 0.087 |
| t-tau | 0.271(-0.011,0.552) | 0.059 |  | 0.517(0.262,0.771) | **1.85e-4*** |  | 0.731(-0.018,1.480) | 0.055 |
| CSF neuroinflammatory biomarkers | | | | | |  |  |  |
| sTNFR1 | 0.254(0.061,0.448) | **0.012** |  | 0.390(0.197,0.583) | **1.90e-4*** |  | 0.342(-0.197,0.880) | 0.182 |
| sTNFR2 | 0.180(-0.133,0.494) | 0.251 |  | 0.431(0.259,0.602) | **8.27e-6*** |  | 0.420(-0.095,0.936) | 0.098 |
| TGF-β1 | -0.045(-0.342,0.251) | 0.758 |  | 0.234(-0.075,0.543) | 0.134 |  | 0.428(-0.542,1.398) | 0.339 |
| IL-10 | -0.257(-0.751,0.237) | 0.298 |  | 0.064(-0.313,0.442) | 0.733 |  | 0.072(-0.857,1.001) | 0.863 |
| ICAM1 | 0.303(-0.126,0.732) | 0.160 |  | 0.359(-0.110,0.828) | 0.130 |  | 0.046(-0.532,0.625) | 0.858 |
| VCAM1 | 0.539(0.172,0.906) | **0.005*** |  | 0.454(0.073,0.836) | **0.021** |  | 0.213(-0.732,1.159) | 0.617 |
| IL-6 | -0.317(-0.925,0.290) | 0.296 |  | -0.068(-0.593,0.458) | 0.797 |  | 0.217(-0.842,1.276) | 0.649 |
| IL-7 | -0.844(-1.811,0.123) | 0.085 |  | 0.509(-0.226,1.244) | 0.170 |  | 0.355(-1.298,2.007) | 0.634 |
| MRI brain structures | | | | | |  |  |  |
| Hippocampus | -0.008(-0.121,0.105) | 0.886 |  | -7.636e-3(-0.183,0.168) | 0.930 |  | -0.185(-0.392,0.022) | 0.068 |
| Entorhinal cortex | -0.172(-0.388,0.044) | 0.115 |  | -0.106(-0.414,0.203) | 0.491 |  | -0.232(-1.456,0.992) | 0.626 |
| Mid temporal lobe | 0.026(-0.091,0.144) | 0.649 |  | -0.022(-0.141,0.098) | 0.716 |  | -0.112(-0.494,0.269) | 0.460 |
| Whole brain | 0.023(-0.025,0.071) | 0.333 |  | 0.012(-0.031,0.055) | 0.587 |  | -0.097(-0.249,0.055) | 0.163 |
| Cognitive scores | | | | | |  |  |  |
| MMSE | -0.001(-0.036,0.034) | 0.953 |  | -0.008(-0.070,0.055) | 0.807 |  | -0.112(-0.270,0.046) | 0.142 |
| MEM | -0.029(-1.071,1.013) | 0.955 |  | -0.133(-1.220,0.953) | 0.806 |  | -1.053(-2.711,0.606) | 0.181 |
| LAN | -0.017(-1.153,1.117) | 0.975 |  | -0.468(-1.301,0.365) | 0.263 |  | -0.185(-3.087,2.717) | 0.887 |
| EF | 0.512(-0.339,1.364) | 0.230 |  | 0.076(-0.918,1.071) | 0.878 |  | -1.012(-3.877,1.853) | 0.439 |
| VP | 0.923(-0.202,2.048) | 0.096 |  | 0.305(-0.578,1.188) | 0.484 |  | 0.201(-2.145,2.548) | 0.845 |
| FAQ | -1.066(-3.956,1.463) | 0.397 |  | -8.576(-20.948,3.796) | 0.169 |  | 33.042(2.223,63.862) | **0.039** |
| ADAS13 | 0.151(-0.420,0.722) | 0.594 |  | 0.056(-0.290,0.402) | 0.745 |  | 0.253(-0.322,0.827) | 0.340 |

P values were obtained by multiple linear regressions models adjusted for age, sex, years of education, *APOE ɛ4* status and intracranial volume (when appropriate).

Significance at the level of *P* < 0.05 were shown in bold.

* Significant after false discovery rate correction.

Abbreviations: CSF, cerebrospinal fluid; CN: cognitively normal; MCI: mild cognitive impairment; AD: Alzheimer’s disease; N: number of sample; *APOE ε4*, apolipoprotein *E4*; CI, confidence interval; Aβ, amyloid-β; p-tau, phosphorylated tau; t-tau, total tau; sTNFR, soluble tumor necrosis factor receptor; TGF, transforming growth factor; IL, interleukin; ICAM1, intercellular cell adhesion molecule-1; VCAM1, vascular cell adhesion molecule-1; MRI, Magnetic resonance imaging; MMSE, Mini-Mental State Examination; MEM, memory function; LAN, language; EF, executive function; VP, visuospatial function; FAQ, Functional Assessment Questionnaire; ADAS13, Alzheimer’s disease Assessment Scale 13.

**Table S2** Relationship of CSF YKL-40 with AD-related pathologies and neuroinflammatory biomarkers stratified by T status.

|  | T negative  (N=100) | |  | T positive  (N=188) | |
| --- | --- | --- | --- | --- | --- |
|  | β (95% CI) | *P* value |  | β (95% CI) | *P* value |
| CSF AD biomarkers | | | | | |
| Aβ_42_ | 0.155(-0.368,0.678) | 0.550 |  | -0.035(-0.462,0.393) | 0.872 |
| p-tau | 0.177(-0.044,0.398) | 0.113 |  | 0.423(0.212,0.634) | **1.76e-4*** |
| t-tau | 0.239(0.034,0.444) | **0.024** |  | 0.395(0.211,0.580) | **6.67e-5*** |
| CSF neuroinflammatory biomarkers | | | | | |
| sTNFR1 | 0.273(0.054,0.491) | **0.016** |  | 0.324(0.163,0.485) | **1.67e-4*** |
| sTNFR2 | 0.323(-0.072,0.718) | 0.106 |  | 0.366(0.210,0.522) | **1.69e-5*** |
| TGF-β1 | 0.151(0.032,0.546) | 0.328 |  | 0.162(-0.122,0.445) | 0.258 |
| IL-10 | 0.197(-0.411,0.804) | 0.514 |  | 0.053(-0.234,0.339) | 0.715 |
| ICAM1 | 0.395(-0.063,0.853) | 0.089 |  | 0.276(-0.118,0.670) | 0.166 |
| VCAM1 | 0.418(0.032,0.805) | **0.035** |  | 0.459(0.134,0.784) | **0.006*** |
| IL-6 | -0.140(-0.867,0.588) | 0.699 |  | -0.023(-0.428,0.383) | 0.912 |
| IL-7 | -0.467(-0.146,0.526) | 0.346 |  | 0.332(-0.354,1.017) | 0.337 |
| MRI brain structures | | | | | |
| Hippocampus | -0.031(-0.228,0.290) | 0.807 |  | -0.092(-0.225,0.040) | 0.169 |
| Entorhinal cortex | -0.022(-0.387,0.343) | 0.902 |  | -0.257(-0.478,-0.036) | **0.023** |
| Mid temporal lobe | 0.097(-0.061,0.255) | 0.217 |  | -0.061(-0.159,0.037) | 0.218 |
| Whole brain | 0.049(-5.719e-5,0.098) | 0.050 |  | -0.023(-0.067,0.026) | 0.356 |
| Cognitive scores | | | | | |
| MMSE | 0.045(-0.039,0.129) | 0.284 |  | -0.081(-0.151,-0.010) | **0.024** |
| MEM | 0.464(-1.315,2.243) | 0.599 |  | -0.877(-2.089,0.334) | 0.153 |
| LAN | 0.644(-0.900,2.189) | 0.402 |  | -0.800(-1.702,0.101) | 0.081 |
| EF | 0.796(-0.455,2.046) | 0.204 |  | -0.719(-1.697,0.260) | 0.147 |
| VP | 0.783(-0.182,1.749) | 0.101 |  | 0.426(-0.368,1.221) | 0.284 |
| FAQ | -5.510(-17.518,6.578) | 0.360 |  | 9.266(-2.906,21.435) | 0.133 |
| ADAS13 | -0.064(-0.742,0.614) | 0.849 |  | 0.383(-0.075,0.842) | 0.100 |

P values were obtained by multiple linear regressions models adjusted for age, sex, years of education, *APOE ɛ4* status and intracranial volume (when appropriate).

Significance at the level of *P* < 0.05 were shown in bold.

* Significant after false discovery rate correction.

Abbreviations: CSF, cerebrospinal fluid; N, number of sample, *APOE ε4*, apolipoprotein *E4*; CI, confidence interval; Aβ, amyloid-β; p-tau, phosphorylated tau; t-tau, total tau; sTNFR, soluble tumor necrosis factor receptor; TGF, transforming growth factor; IL, interleukin; ICAM1, intercellular cell adhesion molecule-1; VCAM1, vascular cell adhesion molecule-1; MRI, Magnetic resonance imaging; MMSE, Mini-Mental State Examination; MEM, memory function; LAN, language; EF, executive function; VP, visuospatial function; FAQ, Functional Assessment Questionnaire; ADAS13, Alzheimer’s disease Assessment Scale 13.

**Table S3** Relationship of CSF YKL-40 with AD-related pathologies and neuroinflammatory biomarkers stratified by T status and hypertensive status.

|  | T-HTN-  (N=59) | |  | T-HTN+  (N=41) | |  | T+HTN-  (N=98) | |  | T+HTN+  (N=90) | |
| --- | --- | --- | --- | --- | --- | --- | --- | --- | --- | --- | --- |
|  | β (95% CI) | *P* value |  | β (95% CI) | *P* value |  | β (95% CI) | *P* value |  | β (95% CI) | *P* value |
| CSF AD biomarkers | | | | | | | | |  |  |  |
| Aβ_42_ | 0.636(0.042,1.231) | **0.038** |  | -0.271(-1.339,0.797) | 0.585 |  | 0.033(-0.526,0.592) | 0.904 |  | -0.179(-0.912,0.554) | 0.622 |
| p-tau | 0.198（-0.135,0.531） | 0.225 |  | 0.193(-0.238,0.624) | 0.341 |  | 0.148(-0.090,0.387) | 0.211 |  | 0.594(0.275,0.915) | **6.69e-4*** |
| t-tau | 0.295(0.003,0.587) | **0.048** |  | 0.229(-0.176,0.634) | 0.237 |  | 0.170(-0.043,0.384) | 0.112 |  | 0.530(0.243,0.818) | **7.23e-4*** |
| CSF neuroinflammatory biomarkers | | | | | | | | |  |  |  |
| sTNFR1 | 0.445(0.163,0.728) | **0.004*** |  | 0.013(-0.470,0.496) | 0.953 |  | 0.222(0.003,0.441) | **0.047** |  | 0.389(0.129,0.650) | **0.005*** |
| sTNFR2 | 0.374(0.100,0.647) | **0.011*** |  | 0.506(-0.731,1.742) | 0.384 |  | 0.250(0.037,0.463) | **0.023** |  | 0.429(0.174,0.683) | **0.002*** |
| TGF-β1 | 0.244(-0.243,0.730) | 0.304 |  | 0.101(-0.552,0.754) | 0.738 |  | -0.144(-0.633,0.346) | 0.549 |  | 0.342(-0.038,0.722) | 0.076 |
| IL-10 | -0.044(-0.833,0.745) | 0.907 |  | 0.464(-0.940,1.868) | 0.479 |  | -0.015(-0.381,0.352) | 0.935 |  | 0.174(-0.279,0.627) | 0.438 |
| ICAM1 | 0.431(-0.340,1.202) | 0.253 |  | 0.025(-0.656,0.707) | 0.935 |  | 0.334(-0.178,0.846) | 0.190 |  | 0.221(-0.421,0.863) | 0.488 |
| VCAM1 | 0.447(-0.074,0.969) | 0.088 |  | 0.432(-0.541,1.404) | 0.346 |  | 0.456(-0.055,0.966) | 0.078 |  | 0.477(-0.031,0.985) | 0.065 |
| IL-6 | -0.590(-1.432,0.252) | 0.157 |  | 0.773(-0.909,2.454) | 0.330 |  | -0.359(-0.967,0.248) | 0.233 |  | 0.138(-0.447,0.723) | 0.633 |
| IL-7 | -1.067(-2.342,0.207) | 0.095 |  | 0.328(-1.621,2.277) | 0.716 |  | 0.241(-0.834,1.316) | 0.647 |  | 0.578(-0.480,1.636) | 0.273 |
| MRI brain structures | | | | | | | | |  |  |  |
| Hippocampus | 0.184(-0.172,0.540) | 0.284 |  | 1.119(-0.382,0.620) | 0.591 |  | -0.081(-0.272,0.111) | 0.388 |  | -0.135(-0.368,0.097) | 0.241 |
| Entorhinal cortex | 0.107(0.404,0.619) | 0.658 |  | 5.965e-3(-0.725,0.737) | 0.985 |  | -0.201(-0.491,0.009) | 0.162 |  | -0.335(-0.733,0.064) | 0.096 |
| Mid temporal lobe | 0.181(-0.041,0.404) | 0.102 |  | 0.127(-0.258,0.511) | 0.462 |  | -0.099(-0.243,0.045) | 0.166 |  | -0.022(-0.147,0.190) | 0.790 |
| Whole brain | 0.059(-0.002,0.119) | 0.056 |  | 0.088(-0.008,0.184) | 0.068 |  | -0.064(-0.129,-0.002) | **0.045** |  | 0.008(-0.074,0.090) | 0.844 |
| Cognitive scores | | | | | | | | |  |  |  |
| MMSE | 0.110(-0.020,0.240) | 0.093 |  | -0.014(-0.170,0.142) | 0.844 |  | -0.054(-0.156,0.048) | 0.296 |  | -0.100(-0.200,-0.001) | **0.049** |
| MEM | 1.728(-0.632,4.089) | 0.140 |  | -0.624(-4.152,2.903) | 0.702 |  | -0.706(-2.598,1.187) | 0.461 |  | -1.522(-3.248,0.204) | 0.082 |
| LAN | 1.027(-0.769,2.823) | 0.243 |  | 0.668(-3.458,4.795) | 0.726 |  | -1.186(-2.632,0.260) | 0.107 |  | -0.699(-1.980,0.583) | 0.274 |
| EF | 1.266(-0.400,2.932) | 0.127 |  | 0.767(-2.079,3.612) | 0.562 |  | -1.765(-3.200,-0.330) | **0.017** |  | -0.453(-1.810,0.905) | 0.501 |
| VP | - | - |  | 0.997(-0.318,2.313) | 0.103 |  | 0.058(-1.136,1.251) | 0.923 |  | 0.393(-1.001,1.786) | 0.561 |
| FAQ | -5.014(-16.322,6.294) | 0.361 |  | -12.928(-39.020,13.164) | 0.295 |  | 9.314(-7.561,26.189) | 0.276 |  | 14.160(-3.857,32.177) | 0.119 |
| ADAS13 | -0.444(-1.327,0.438) | 0.302 |  | 0.149(-1.355,1.652) | 0.830 |  | 0.048(-0.670,0.766) | 0.895 |  | 0.641(-0.004,1.286) | 0.052 |

P values were obtained by multiple linear regressions models adjusted for age, sex, years of education, *APOE ɛ4* status and intracranial volume (when appropriate).

Significance at the level of *P* < 0.05 were shown in bold.

* Significant after false discovery rate correction.

Abbreviations: CSF, cerebrospinal fluid; T-HTN-: T negative and normotension; T-HTN+: T positive and hypertension; T+HTN-: T positive and normotension; T+HTN+: T positive and hypertension; N, number of sample, *APOE ε4*, apolipoprotein *E4*; CI, confidence interval; Aβ, amyloid-β; p-tau, phosphorylated tau; t-tau, total tau; sTNFR, soluble tumor necrosis factor receptor; TGF, transforming growth factor; IL, interleukin; ICAM1, intercellular cell adhesion molecule-1; VCAM1, vascular cell adhesion molecule-1; MRI, Magnetic resonance imaging; MMSE, Mini-Mental State Examination; MEM, memory function; LAN, language; EF, executive function; VP, visuospatial function; FAQ, Functional Assessment Questionnaire; ADAS13, Alzheimer’s disease Assessment Scale 13.

**Table S4** Relationship of CSF YKL-40 with AD-related pathologies and neuroinflammatory biomarkers stratified by A status.

|  | A negative  (N=106) | |  | A positive  (N=182) | |
| --- | --- | --- | --- | --- | --- |
|  | β (95% CI) | *P* value |  | β (95% CI) | *P* value |
| CSF AD biomarkers | | | | | |
| Aβ_42_ | -0.094(-0.401,0.213) | 0.538 |  | -0.052(-0.329,0.224) | 0.705 |
| p-tau | 0.476(0.155,0.796) | **0.005*** |  | 0.565(0.302,0.829) | **7.01e-5*** |
| t-tau | 0.448(0.145,0.752) | **0.005*** |  | 0.533(0.313,0.754) | **1.01e-5*** |
| CSF neuroinflammatory biomarkers | | | | | |
| sTNFR1 | 0.277(0.063,0.490) | **0.013** |  | 0.364(0.217,0.512) | **7.63e-6*** |
| sTNFR2 | 0.194(-0.121,0.510) | 0.220 |  | 0.389(0.247,0.531) | **1.08e-6*** |
| TGF-β1 | 0.182(-0.166,0.531) | 0.296 |  | 0.156(-0.083,0.395) | 0.196 |
| IL-10 | -0.211(-0.692,0.270) | 0.379 |  | 0.027(-0.265,0.318) | 0.855 |
| ICAM1 | 0.504(0.034,0.973) | **0.036** |  | 0.209(-0.136,0.554) | 0.230 |
| VCAM1 | 0.562(0.117,1.008) | **0.015*** |  | 0.454(0.166,0.742) | **0.003*** |
| IL-6 | -0.345(-1.007,0.317) | 0.297 |  | 0.056(-0.330,0.442) | 0.773 |
| IL-7 | -0.363(-1.256,0.531) | 0.416 |  | 0.253(-0.387,0.894) | 0.431 |
| MRI brain structures | | | | | |
| Hippocampus | -0.095(-0.268,0.078) | 0.273 |  | -0.019(-0.173,0.135) | 0.807 |
| Entorhinal cortex | -0.273(-0.470,-0.075) | **0.008*** |  | -0.097(-0.348,0.153) | 0.437 |
| Mid temporal lobe | -0.012(-0.158,0.134) | 0.872 |  | -0.034(-0.125,0.056) | 0.451 |
| Whole brain | 0.016(-0.038,0.070) | 0.560 |  | -0.009(-0.034,0.052) | 0.660 |
| Cognitive scores | | | | | |
| MMSE | -0.037(-0.111,0.036) | 0.310 |  | -0.060(-0.130,0.011) | 0.095 |
| MEM | 0.032(-1.314,1.378) | 0.962 |  | -0.970(-2.189,0.249) | 0.117 |
| LAN | 0.111(-1.104,1.327) | 0.854 |  | -0.938(-1.799,-0.077) | **0.033** |
| EF | 0.390(-0.741,1.520) | 0.489 |  | -0.678(-1.647,0.292) | 0.167 |
| VP | 0.649(-1.273,2.572) | 0.473 |  | 0.414(-0.302,1.130) | 0.249 |
| FAQ | 3.681(-9.986,17.348) | 0.588 |  | 7.496(-4.197,19.189) | 0.204 |
| ADAS13 | 0.115(-0.483,0.713) | 0.699 |  | 0.363(-0.055,0.780) | 0.087 |

P values were obtained by multiple linear regressions models adjusted for age, sex, years of education, *APOE ɛ4* status and intracranial volume (when appropriate).

Significance at the level of *P* < 0.05 were shown in bold.

* Significant after false discovery rate correction.

Abbreviations: CSF, cerebrospinal fluid; N, number of sample, *APOE ε4*, apolipoprotein *E4*; CI, confidence interval; Aβ, amyloid-β; p-tau, phosphorylated tau; t-tau, total tau; sTNFR, soluble tumor necrosis factor receptor; TGF, transforming growth factor; IL, interleukin; ICAM1, intercellular cell adhesion molecule-1; VCAM1, vascular cell adhesion molecule-1; MRI, Magnetic resonance imaging; MMSE, Mini-Mental State Examination; MEM, memory function; LAN, language; EF, executive function; VP, visuospatial function; FAQ, Functional Assessment Questionnaire; ADAS13, Alzheimer’s disease Assessment Scale 13.

**Table S5** Relationship of CSF YKL-40 with AD-related pathologies and neuroinflammatory biomarkers stratified by A status and hypertensive status.

|  | A-HTN-  (N=54) | |  | A-HTN+  (N=52) | |  | A+HTN-  (N=103) | |  | A+HTN+  (N=79) | |
| --- | --- | --- | --- | --- | --- | --- | --- | --- | --- | --- | --- |
|  | β (95% CI) | *P* value |  | β (95% CI) | *P* value |  | β (95% CI) | *P* value |  | β (95% CI) | *P* value |
| CSF AD biomarkers | | | | | | | | |  |  |  |
| Aβ_42_ | 0.108(-0.284,0.500) | 0.562 |  | -0.105(-0.684,0.474) | 0.707 |  | 0.007(-0.453,0.467) | 0.974 |  | -0.083(-0.561,0.395) | 0.723 |
| p-tau | 0.397（-0.121,0.914） | 0.122 |  | 0.656(0.135,1.177) | **0.017** |  | 0.559(0.161,0.958) | **0.008*** |  | 0.583(0.134,1.031) | **0.013*** |
| t-tau | 0.384(-0.115,0.883) | 0.120 |  | 0.564(0.045,1.083) | **0.035** |  | 0.525(0.176,0.875) | **0.005*** |  | 0.552(0.192,0.912) | **0.004*** |
| CSF neuroinflammatory biomarkers | | | | | | | | |  |  |  |
| sTNFR1 | 0.280(-0.057,0.617) | 0.096 |  | 0.287(-0.082,0.656) | 0.119 |  | 0.359(0.113,0.605) | **0.006*** |  | 0.360(0.116,0.603) | **0.006*** |
| sTNFR2 | 0.215(-0.155,0.586) | 0.231 |  | 0.238(-0.372,0.849) | 0.422 |  | 0.330(0.129,0.531) | **0.002*** |  | 0.418(0.149,0.686) | **0.004*** |
| TGF-β1 | 0.341(-0.426,1.109) | 0.354 |  | 0.304(-0.214,0.822) | 0.232 |  | 0.015(-0.385,0.414) | 0.941 |  | 0.260(-0.115,0.635) | 0.165 |
| IL-10 | -0.301(-1.157,0.555) | 0.461 |  | 0.127(-0.599,0.844) | 0.724 |  | -0.006(-0.459,0.447) | 0.978 |  | 0.258(-0.240,0.756) | 0.295 |
| ICAM1 | -0.059(-1.052,0.933) | 0.899 |  | 0.566(-0.097,1.230) | 0.089 |  | 0.228(-0.185,0.642) | 0.266 |  | 0.221(-0.475,0.916) | 0.518 |
| VCAM1 | 0.805(-0.250,1.861) | 0.123 |  | 0.560(-0.082,1.201) | 0.082 |  | 0.523(0.084,0.962) | **0.021** |  | 0.490(-0.026,1.006) | 0.062 |
| IL-6 | -0.488(-1.614,0.639) | 0.367 |  | 0.421(-0.526,1.368) | 0.361 |  | -0.215(-0.860,0.429) | 0.498 |  | 0.305(-0.314,0.924) | 0.319 |
| IL-7 | -1.771(-3.566,0.023) | 0.053 |  | 0.494(-0.737,1.725) | 0.409 |  | 0.510(-0.364,1.384) | 0.241 |  | 0.185(-1.037,1.406) | 0.758 |
| MRI brain structures | | | | | | | | |  |  |  |
| Hippocampus | 0.030(-0.377,0.437) | 0.873 |  | 0.131(-0.384,0.121) | 0.283 |  | -0.026(-0.213,0.265) | 0.822 |  | -0.214(-0.507,0.079) | 0.141 |
| Entorhinal cortex | -0.253(0.632,0.126) | 0.170 |  | -0.335(-0.629,0.041) | **0.028** |  | -0.016(-0.410,0.378) | 0.932 |  | -0.381(-0.820,0.058) | 0.085 |
| Mid temporal lobe | 0.008(-0.313,0.297) | 0.955 |  | 9.288e-4(-0.238,0.240) | 0.993 |  | -0.033(-0.183,0.117) | 0.653 |  | -0.007(-0.157,0.172) | 0.926 |
| Whole brain | 0.014(-0.084,0.112) | 0.761 |  | 0.024(-0.074,0.122) | 0.610 |  | -0.026(-0.092,-0.041) | 0.431 |  | 0.028(-0.045,0.102) | 0.431 |
| Cognitive scores | | | | | | | | |  |  |  |
| MMSE | -0.009(-0.142,0.124) | 0.882 |  | -0.052(-0.168,0.064) | 0.355 |  | -0.059(-0.173,0.056) | 0.304 |  | -0.090(-0.200,0.021) | 0.107 |
| MEM | 0.532(-1.927,2.992) | 0.648 |  | -0.044(-2.338,2.250) | 0.968 |  | -0.338(-2.233,1.556) | 0.716 |  | -2.055(-3.910,-0.200) | **0.031** |
| LAN | -0.130(-2.172,1.912) | 0.893 |  | 0.631(-1.516,2.777) | 0.544 |  | -1.181(-2.669,0.301) | 0.115 |  | -0.935(-2.250,0.379) | 0.155 |
| EF | -0.181(-1.885,1.523) | 0.822 |  | 0.615(-1.117,2.347) | 0.464 |  | -0.849(-2.561,0.865) | 0.318 |  | -0.432(-1.941,1.078) | 0.560 |
| VP | 3.534(1.185,5.886) | **0.033** |  | 1.116(-2.251,4.483) | 0.433 |  | 1.094(-0.130,2.317) | 0.076 |  | 0.358(-0.828,1.544) | 0.533 |
| FAQ | 1.229(-2.124,4.581) | 0.443 |  | 0.836(-25.597,27.269) | 0.948 |  | 11.399(-8.185,30.984) | 0.242 |  | 10.063(-9.446,29.572) | 0.297 |
| ADAS13 | -0.445(-1.078,0.988) | 0.926 |  | 0.183(-0.865,1.232) | 0.717 |  | 0.293(-0.422,1.008) | 0.407 |  | 0.644(0.061,1.228) | **0.032** |

P values were obtained by multiple linear regressions models adjusted for age, sex, years of education, *APOE ɛ4* status and intracranial volume (when appropriate).

Significance at the level of *P* < 0.05 were shown in bold.

* Significant after false discovery rate correction.

Abbreviations: CSF, cerebrospinal fluid; A-HTN-: A negative and normotension; A-HTN+: A positive and hypertension; A+HTN-: A positive and normotension; A+HTN+: A positive and hypertension; N, number of sample, *APOE ε4*, apolipoprotein *E4*; CI, confidence interval; Aβ, amyloid-β; p-tau, phosphorylated tau; t-tau, total tau; sTNFR, soluble tumor necrosis factor receptor; TGF, transforming growth factor; IL, interleukin; ICAM1, intercellular cell adhesion molecule-1; VCAM1, vascular cell adhesion molecule-1; MRI, Magnetic resonance imaging; MMSE, Mini-Mental State Examination; MEM, memory function; LAN, language; EF, executive function; VP, visuospatial function; FAQ, Functional Assessment Questionnaire; ADAS13, Alzheimer’s disease Assessment Scale 13.

**Table S6** Relationship of CSF YKL-40 with AD-related pathologies and neuroinflammatory biomarkers stratified by A status and clinical diagnosis.

|  | A-CN  (N=56) | |  | A+CN  (N=28) | |  | A-MCI  (N=40) | |  | A+MCI  (N=87) | |  | AD  (N=77) | |
| --- | --- | --- | --- | --- | --- | --- | --- | --- | --- | --- | --- | --- | --- | --- |
|  | β (95% CI) | *P* value |  | β (95% CI) | *P* value |  | β (95% CI) | *P* value |  | β (95% CI) | *P* value |  | β (95% CI) | *P* value |
| CSF AD biomarkers | | | | | | | | |  |  |  |  |  |  |
| Aβ_42_ | -0.054(-0.539,0.431) | 0.818 |  | -0.404(-1.028,0.219) | 0.169 |  | 0.316(-0.532,1.164) | 0.421 |  | 0.076(-0.295,0.447) | 0.676 |  | 0.446(-0.414,1.306) | 0.266 |
| p-tau | 0.095(-0.311,0.501） | 0.630 |  | 0.663(-0.224,1.551) | 0.120 |  | 0.466(-0.489,1.420) | 0.298 |  | 0.511(0.174,0.848) | **0.004*** |  | 0.740(-0.134,1.615) | 0.087 |
| t-tau | 0.089(-0.309,0.488) | 0.645 |  | 0.593(-0.107,1.293) | 0.085 |  | 0.523(-0.368,1.414) | 0.217 |  | 0.492(0.211,0.773) | **0.001*** |  | 0.731(-0.018,1.480) | 0.055 |
| CSF neuroinflammatory biomarkers | | | | | | | | |  |  |  |  |  |  |
| sTNFR1 | 0.146(-0.171,0.463) | 0.348 |  | 0.397(0.096,0.699) | **0.017** |  | 0.319(-0.291,0.929) | 0.267 |  | 0.417(0.217,0.616) | **1.97e-4*** |  | 0.342(-0.197,0.880) | 0.182 |
| sTNFR2 | -0.082(-0.583,0.419) | 0.737 |  | 0.316(0.070,0.562) | **0.019** |  | 0.292(-0.353,0.936) | 0.333 |  | 0.452(0.279,0.626) | **1.13e-5*** |  | 0.420(-0.095,0.936) | 0.098 |
| TGF-β1 | -0.022(-0.525,0.481) | 0.929 |  | -0.042(-0.435,0.350) | 0.807 |  | 0.440(-0.596,1.475) | 0.362 |  | 0.193(-0.150,0.536) | 0.259 |  | 0.428(-0.542,1.398) | 0.339 |
| IL-10 | -0.535(-1.246,0.176) | 0.132 |  | -0.181(-0.938,0.576) | 0.590 |  | 0.146(-1.149,1.441) | 0.804 |  | 0.012(-0.385,0.409) | 0.951 |  | 0.072(-0.857,1.001) | 0.863 |
| ICAM1 | 0.426(-0.276,1.127) | 0.220 |  | 0.075(-0.485,0.635) | 0.761 |  | 0.079(-1.249,1.407) | 0.896 |  | 0.342(-0.186,0.870) | 0.159 |  | 0.046(-0.532,0.625) | 0.858 |
| VCAM1 | 0.443(-0.111,0.997) | 0.111 |  | 0.730(-0.107,1.566) | 0.078 |  | 0.859(-0.852,2.570) | 0.285 |  | 0.451(0.112,0.790) | **0.011*** |  | 0.213(-0.732,1.159) | 0.617 |
| IL-6 | -0.568(-1.472,0.337) | 0.205 |  | -0.205(-1.195,0.784) | 0.639 |  | -0.097(-2.271,2.077) | 0.922 |  | -0.030(-0.543,0.483) | 0.906 |  | 0.217(-0.842,1.276) | 0.649 |
| IL-7 | -1.133(-2.591,0.324) | 0.121 |  | -0.476(-2.208,1.255) | 0.536 |  | 0.482(-0.780,1.744) | 0.410 |  | 0.392(-0.443,1.227) | 0.345 |  | 0.355(-1.298,2.007) | 0.634 |
| MRI brain structures | | | | | | | | |  |  |  |  |  |  |
| Hippocampus | 0.016(-0.139,0.171) | 0.832 |  | 0.114(-0.243,0.472) | 0.448 |  | -0.173(-0.911,0.564) | 0.595 |  | -9.76e-3(-0.216,0.196) | 0.992 |  | -0.185(-0.392,0.022) | 0.068 |
| Entorhinal cortex | -0.371(-0.647,-0.094) | **0.012*** |  | -0.126(-0.337,0.650) | 0.089 |  | 0.077(-0.768,0.921) | 0.836 |  | -0.128(-0.508,0.253) | 0.493 |  | -0.232(-1.456,0.992) | 0.626 |
| Mid temporal lobe | -0.031(-0.213,0.151) | 0.721 |  | 0.002(-0.311,0.314) | 0.990 |  | -0.321(-0.753,0.111) | 0.122 |  | -2.51e-4(-0.113,0.112) | 0.996 |  | -0.112(-0.494,0.269) | 0.460 |
| Whole brain | 0.012(-0.049,0.072) | 0.685 |  | 0.115(-0.066,0.296) | 0.163 |  | -0.081(-0.212,0.050) | 0.187 |  | 0.012(-0.037,0.060) | 0.622 |  | -0.097(-0.249,0.055) | 0.163 |
| Cognitive scores | | | | | | | | |  |  |  |  |  |  |
| MMSE | 0.009(-0.040,0.059) | 0.704 |  | -0.010(-0.104,0.084) | 0.805 |  | 0.075(-0.125,0.276) | 0.419 |  | -0.011(-0.078,0.055) | 0.734 |  | -0.112(-0.270,0.046) | 0.142 |
| MEM | 0.957(-0.332,2.246) | 0.137 |  | -1.049(-3.328,1.231) | 0.313 |  | 0.741(-1.553,3.035) | 0.484 |  | -0.074(-1.343,1.194) | 0.906 |  | -1.053(-2.711,0.606) | 0.181 |
| LAN | 0.060(-1.518,1.637) | 0.938 |  | -1.081(-3.276,1.114) | 0.282 |  | 1.513(-0.679,3.704) | 0.153 |  | -0.671(-1.571,0.229) | 0.138 |  | -0.185(-3.087,2.717) | 0.887 |
| EF | 0.984(-0.227,2.195) | 0.106 |  | 0.503(-1.573,2.578) | 0.585 |  | 4.075(1.138,7.011) | **0.012** |  | -0.429(-1.383,0.524) | 0.364 |  | -1.012(-3.877,1.853) | 0.439 |
| VP | 3.720(-0.848,8.287) | 0.073 |  | 0.741(-4.338,5.820) | 0.315 |  | 2.632(-3.549,8.812) | 0.268 |  | 0.269(-0.658,1.196) | 0.551 |  | 0.201(-2.145,2.548) | 0.845 |
| FAQ | -3.158(-7.536,1.219) | 0.148 |  | - | - |  | -35.870(-70.377,-1.362) | **0.043** |  | -2.712(-15.009,9.585) | 0.655 |  | 33.042(2.223,63.862) | **0.039** |
| ADAS13 | -0.092(-0.855,0.671) | 0.804 |  | 0.315(-0.951,1.580) | 0.575 |  | -0.401(-1.061,0.260) | 0.203 |  | 0.081(-0.297,0.459) | 0.665 |  | 0.253(-0.322,0.827) | 0.340 |

P values were obtained by multiple linear regressions models adjusted for age, sex, years of education, *APOE ɛ4* status and intracranial volume (when appropriate).

Significance at the level of *P* < 0.05 were shown in bold.

* Significant after false discovery rate correction.

Abbreviations: CSF, cerebrospinal fluid; A-CN: A negative and cognitively normal; A+CN: A positive and cognitively normal; A-MCI: A negative and mild cognitive impairment; A+MCI: A positive and mild cognitive impairment; AD: Alzheimer’s disease; N, number of sample, *APOE ε4*, apolipoprotein *E4*; CI, confidence interval; Aβ, amyloid-β; p-tau, phosphorylated tau; t-tau, total tau; sTNFR, soluble tumor necrosis factor receptor; TGF, transforming growth factor; IL, interleukin; ICAM1, intercellular cell adhesion molecule-1; VCAM1, vascular cell adhesion molecule-1; MRI, Magnetic resonance imaging; MMSE, Mini-Mental State Examination; MEM, memory function; LAN, language; EF, executive function; VP, visuospatial function; FAQ, Functional Assessment Questionnaire; ADAS13, Alzheimer’s disease Assessment Scale 13.

**Table S7** Mediation effects of CSF neuroinflammatory biomarkers stratified by clinical diagnosis.

|  | | | p-tau | | | |  | t-tau | | | |
| --- | --- | --- | --- | --- | --- | --- | --- | --- | --- | --- | --- |
|  |  |  | Indirect effect | Direct effect | Total effect | Proportion Mediated |  | Indirect effect | Direct effect | Total effect | Proportion Mediated |
| CN | ICAM | Coefficient (95%CI) | 0.039(-0.040,0.160) | 0.251(-0.206,0.610) | 0.290(-0.160,0.610) | 0.133(-1.268,1.680) |  | 0.049(-0.022,0.170) | 0.221(-0.134,0.510) | 0.271(-0.087,0.540) | 0.182(-0.773,2.100) |
|  |  | *P* value | 0.400 | 0.320 | 0.230 | 0.530 |  | 0.240 | 0.230 | 0.130 | 0.290 |
|  | VCAM | Coefficient (95%CI) | 0.203(0.009,0.390) | 0.087(-0.238,0.450) | 0.290(-0.107,0.610) | 0.700(-4.106,5.450) |  | 0.238(0.063,0.430) | 0.032(-0.219,0.280) | 0.271(-0.069,0.550) | 0.881(-2.118,4.940) |
|  |  | *P* value | 0.046 | 0.626 | 0.168 | 0.210 |  | 0.008 | 0.814 | 0.114 | 0.110 |
|  | sTNFR1 | Coefficient (95%CI) | 0.226(0.024,0.450) | 0.064(-0.295,0.390) | 0.290(-0.145,0.620) | 0.779(-3.651,4.260) |  | 0.229(0.008,0.440) | 0.042(-0.245,0.300) | 0.271(-0.066,0.540) | 0.846(-2.145,5.290) |
|  |  | *P* value | 0.028 | 0.710 | 0.190 | 0.190 |  | 0.044 | 0.702 | 0.122 | 0.122 |
|  | sTNFR2 | Coefficient (95%CI) | 0.011(-0.035,0.370) | 0.279(-0.204,0.590) | 0.290(-0.111,0.610) | 0.038(-0.713,2.270) |  | 0.023(-0.011,0.380) | 0.248(-0.199,0.520) | 0.271(-0.070,0.550) | 0.084(-0.700,2.530) |
|  |  | *P* value | 0.570 | 0.370 | 0.160 | 0.610 |  | 0.360 | 0.390 | 0.130 | 0.420 |
| MCI | ICAM | Coefficient (95%CI) | 0.079(-0.016,0.230) | 0.444(0.164,0.700) | 0.524(0.243,0.800) | 0.151(-0.033,0.480) |  | 0.080(-0.006,0.220) | 0.437(0.194,0.640) | 0.517(0.275,0.710) | 0.155(-0.015,0.440) |
|  |  | *P* value | 0.108 | 0.002 | <2e-16 | 0.108 |  | 0.088 | 0.004 | <2e-16 | 0.088 |
|  | VCAM | Coefficient (95%CI) | 0.116(0.012,0.300) | 0.407(0.059,0.700) | 0.524(0.235,0.810) | 0.222(0.023,0.790) |  | 0.122(0.024,0.290) | 0.395(0.103,0.630) | 0.517(0.266,0.730) | 0.236(0.052,0.700) |
|  |  | *P* value | 0.028 | 0.024 | <2e-16 | **0.028** |  | 0.002 | 0.006 | <2e-16 | **0.002** |
|  | sTNFR1 | Coefficient (95%CI) | 0.260(0.084,0.480) | 0.264(-0.053,0.570) | 0.524(0.202,0.790) | 0.456(0.174,1.210) |  | 0.266(0.100,0.480) | 0.250(-0.011,0.490) | 0.517(0.279,0.740) | 0.516(0.220,1.030) |
|  |  | *P* value | <2e-16 | 0.11 | <2e-16 | **<2e-16** |  | <2e-16 | 0.066 | <2e-16 | **<2e-16** |
|  | sTNFR2 | Coefficient (95%CI) | 0.400(0.178,0.640) | 0.124(-0.208,0.440) | 0.524(0.219,0.790) | 0.764(0.368,1.620) |  | 0.390(0.189,0.620) | 0.127(-0.124,0.370) | 0.517(0.274,0.720 | 0.755(0.391,1.390) |
|  |  | *P* value | <2e-16 | 0.468 | 0.002 | **0.002** |  | <2e-16 | 0.370 | <2e-16 | **<2e-16** |
| AD | ICAM | Coefficient (95%CI) | 0.024(-1.810,1.160) | 0.717(-0.881,2.460) | 0.740(-0.435,1.520) | 0.032(-7.763,8.260) |  | 0.020(-3.051,1.190) | 0.711(-0.776,3.450) | 0.731(-0.295,1.500) | 0.027(-6.220,7.960) |
|  |  | *P* value | 0.960 | 0.310 | 0.230 | 0.970 |  | 0.990 | 0.250 | 0.140 | 0.950 |
|  | VCAM | Coefficient (95%CI) | 0.023(-0.968,1.130) | 0.717(-0.884,1.830) | 0.740(-0.502,1.600) | 0.031(-4.482,4.870) |  | 0.017(-0.975,0.760) | 0.714(-0.583,1.850) | 0.731(-0.280,1.550) | 0.023(-3.599,3.350) |
|  |  | *P* value | 0.980 | 0.390 | 0.250 | 0.970 |  | 0.990 | 0.220 | 0.130 | 0.990 |
|  | sTNFR1 | Coefficient (95%CI) | 0.401(-0.921,1.470) | 0.339(-0.797,1.880) | 0.740(-0.502,1.640) | 0.541(-3.642,3.420) |  | 0.341(-1.012,1.120) | 0.390(-0.685,2.050) | 0.731(-0.255,1.410) | 0.466(-2.213,3.670) |
|  |  | *P* value | 0.410 | 0.450 | 0.230 | 0.390 |  | 0.440 | 0.270 | 0.120 | 0.400 |
|  | sTNFR2 | Coefficient (95%CI) | 0.506(-1.122,1.820) | 0.235(-1.104,1.900) | 0.740(-0.591,1.750) | 0.683(-6.841,5.350) |  | 0.445(-0.862,1.400) | 0.286(-0.827,1.630) | 0.731(-0.319,1.530) | 0.609(-2.321,3.800) |
|  |  | *P* value | 0.320 | 0.910 | 0.240 | 0.370 |  | 0.370 | 0.610 | 0.160 | 0.330 |

The mediation analyses were adjusted to age, sex, years of education and apolipoprotein *E* (*APOE*) *ɛ4* status.

Significance at the level of *P* < 0.05 were shown in bold.

Abbreviations: CSF, cerebrospinal fluid; CN: cognitively normal; MCI: mild cognitive impairment; AD: Alzheimer’s disease; CI, confidence interval; p-tau, phosphorylated tau; t-tau, total tau; ICAM1, intercellular cell adhesion molecule-1; VCAM1, vascular cell adhesion molecule-1; sTNFR, soluble tumor necrosis factor receptor.

**Table S8** Mediation effects of CSF neuroinflammatory biomarkers stratified by T status.

|  | | | p-tau | | | |  | t-tau | | | |
| --- | --- | --- | --- | --- | --- | --- | --- | --- | --- | --- | --- |
|  |  |  | Indirect effect | Direct effect | Total effect | Proportion Mediated |  | Indirect effect | Direct effect | Total effect | Proportion Mediated |
| T- | ICAM | Coefficient (95%CI) | 0.023(-0.039,0.140) | 0.154(-0.110,0.410) | 0.177(-0.078,0.440) | 0.128(-0.478,1.490) |  | 0.027(-0.042,0.130) | 0.212(-0.029,0.470) | 0.239(-0.002,0.490) | 0.114(-0.277,0.850) |
|  |  | *P* value | 0.530 | 0.230 | 0.170 | 0.530 |  | 0.478 | 0.084 | 0.054 | 0.496 |
|  | VCAM | Coefficient (95%CI) | 0.083(-0.001,0.190) | 0.094(-0.185,0.400) | 0.177(-0.114,0.470) | 0.468(-1.145,3.270) |  | 0.111(0.011,0.230) | 0.128(-0.087,0.370) | 0.239(-0.008,0.510) | 0.464(-0.236,2.620) |
|  |  | *P* value | 0.052 | 0.358 | 0.162 | 0.178 |  | 0.034 | 0.184 | 0.060 | 0.070 |
|  | sTNFR1 | Coefficient (95%CI) | 0.100(-0.012,0.290) | 0.077(-0.147,0.320) | 0.177(-0.083,0.440) | 0.566(-1.512,3.340) |  | 0.127(0.016,0.290) | 0.111(-0.071,0.330) | 0.239(0.005,0.500) | 0.534(-0.004,2.190) |
|  |  | *P* value | 0.096 | 0.450 | 0.166 | 0.210 |  | 0.030 | 0.218 | 0.046 | 0.052 |
|  | sTNFR2 | Coefficient (95%CI) | 0.049(-0.002,0.380) | 0.127(-0.205,0.370) | 0.177(-0.093,0.480) | 0.279(-0.689,3.090) |  | 0.058(-0.002,0.370) | 0.181(-0.117,0.440) | 0.239(0.014,0.530) | 0.243(-0.040,1.840) |
|  |  | *P* value | 0.084 | 0.538 | 0.148 | 0.148 |  | 0.070 | 0.294 | 0.038 | 0.092 |
| T+ | ICAM | Coefficient (95%CI) | 0.049(-0.021,0.120) | 0.374(0.141,0.580) | 0.423(0.171,0.640) | 0.116(-0.080,0.340) |  | 0.055(-0.017,0.130) | 0.340(0.128,0.520) | 0.395(0.177,0.590) | 0.139(-0.058,0.370) |
|  |  | *P* value | 0.156 | 0.004 | 0.002 | 0.158 |  | 0.120 | <2e-16 | <2e-16 | 0.120 |
|  | VCAM | Coefficient (95%CI) | 0.067(-0.014,0.160) | 0.356(0.121,0.570) | 0.423(0.172,0.630) | 0.157(-0.032,0.490) |  | 0.087(0.018,0.170) | 0.309(0.079,0.490) | 0.395(0.167,0.580) | 0.220(0.045,0.580) |
|  |  | *P* value | 0.118 | 0.006 | <2e-16 | 0.118 |  | 0.001 | 0.004 | <2e-16 | **0.010** |
|  | sTNFR1 | Coefficient (95%CI) | 0.224(0.089,0.380) | 0.199(0.023,0.390) | 0.423(0.188,0.640) | 0.528(0.269,0.910) |  | 0.226(0.087,0.350) | 0.169(0.021,0.330) | 0.395(0.172,0.580) | 0.572(0.337,0.920) |
|  |  | *P* value | <2e-16 | 0.034 | <2e-16 | **<2e-16** |  | <2e-16 | 0.030 | <2e-16 | **<2e-16** |
|  | sTNFR2 | Coefficient (95%CI) | 0.249(0.120,0.400) | 0.174(-0.039,0.400) | 0.423(0.180,0.630) | 0.589(0.320,1.130) |  | 0.262(0.132,0.390) | 0.134(-0.045,0.290) | 0.395(0.182,0.580) | 0.662(0.413,1.230) |
|  |  | *P* value | <2e-16 | 0.088 | <2e-16 | **<2e-16** |  | <2e-16 | 0.130 | <2e-16 | **<2e-16** |

The mediation analyses were adjusted to age, sex, years of education and apolipoprotein *E* (*APOE*) *ɛ4* status.

Significance at the level of *P* < 0.05 were shown in bold.

Abbreviations: CSF, cerebrospinal fluid; T-: T negative; T+: T positive; CI, confidence interval; p-tau, phosphorylated tau; t-tau, total tau; ICAM1, intercellular cell adhesion molecule-1; VCAM1, vascular cell adhesion molecule-1; sTNFR, soluble tumor necrosis factor receptor.

**Table S9** Mediation effects of CSF neuroinflammatory biomarkers stratified by T status and hypertensive status.

|  | | | p-tau | | | |  | t-tau | | | |
| --- | --- | --- | --- | --- | --- | --- | --- | --- | --- | --- | --- |
|  |  |  | Indirect effect | Direct effect | Total effect | Proportion Mediated |  | Indirect effect | Direct effect | Total effect | Proportion Mediated |
| T-HTN- | ICAM | Coefficient (95%CI) | 0.044(-0.091,0.320) | 0.154(-0.201,0.680) | 0.198(-0.150,0.750) | 0.221(-2.232,2.830) |  | 0.040(-0.055,0.300) | 0.254(-0.069,0.770) | 0.295(0.013,0.800) | 0.137(-0.274,1.020) |
|  |  | *P* value | 0.520 | 0.410 | 0.290 | 0.540 |  | 0.442 | 0.086 | 0.038 | 0.452 |
|  | VCAM | Coefficient (95%CI) | 0.035(-0.220,0.150) | 0.163(-0.250,0.790) | 0.198(-0.163,0.730) | 0.174(-2.509,2.920) |  | 0.086(-0.073,0.270) | 0.209(-0.112,0.760) | 0.295(-0.035,0.780) | 0.290(-0.635,1.810) |
|  |  | *P* value | 0.690 | 0.330 | 0.270 | 0.860 |  | 0.288 | 0.218 | 0.086 | 0.358 |
|  | sTNFR1 | Coefficient (95%CI) | 0.073(-0.202,0.540) | 0.125(-0.374,0.540) | 0.198(-0.164,0.730) | 0.370(-3.878,5.360) |  | 0.168(-0.043,0.540) | 0.126(-0.236,0.480) | 0.295(-0.001,0.780) | 0.572(-0.632,2.940) |
|  |  | *P* value | 0.590 | 0.580 | 0.290 | 0.640 |  | 0.102 | 0.432 | 0.052 | 0.142 |
|  | sTNFR2 | Coefficient (95%CI) | 0.249(-0.014,0.760) | -0.052(-0.537,0.370) | 0.198(-0.163,0.730) | 1.260(-9.386,7.660) |  | 0.287(0.037,0.670) | 0.008(-0.276,0.340) | 0.295(0.008,0.730) | 0.972(-0.199,3.280) |
|  |  | *P* value | 0.078 | 0.848 | 0.340 | 0.338 |  | 0.024 | 0.878 | 0.046 | 0.058 |
| T-HTN+ | ICAM | Coefficient (95%CI) | -0.004(-0.303,0.190) | 0.197(-0.657,0.750) | 0.193(-0.663,0.650) | -0.020(-1.421,1.300) |  | -0.002(-0.270,0.200) | 0.231(-0.623,0.760) | 0.229(-0.606,0.650) | -0.007(-1.133,1.490) |
|  |  | *P* value | 0.840 | 0.450 | 0.450 | 0.820 |  | 0.860 | 0.440 | 0.430 | 0.850 |
|  | VCAM | Coefficient (95%CI) | 0.117(-0.267,0.490) | 0.076(-0.674,0.690) | 0.193(-0.655,0.670) | 0.604(-1.172,3.040) |  | 0.134(-0.310,0.460) | 0.095(-0.532,0.540) | 0.229(-0.605,0.630) | 0.585(-1.711,2.530) |
|  |  | *P* value | 0.470 | 0.670 | 0.470 | 0.360 |  | 0.450 | 0.580 | 0.460 | 0.230 |
|  | sTNFR1 | Coefficient (95%CI) | 0.009(-0.597,0.380) | 0.184(-0.542,0.700) | 0.193(-0.658,0.670) | 0.046(-2.651,4.140) |  | 0.010(-0.563,0.340) | 0.220(-0.341,0.560) | 0.229(-0.626,0.650) | 0.042(-1.70,4.780) |
|  |  | *P* value | 0.840 | 0.260 | 0.460 | 0.490 |  | 0.800 | 0.170 | 0.450 | 0.380 |
|  | sTNFR2 | Coefficient (95%CI) | 0.040(-0.226,0.630) | 0.153(-0.727,0.750) | 0.193(-0.604,0.700) | 0.207(-0.830,2.820) |  | 0.049(-0.212,0.590) | 0.181(-0.599,0.670) | 0.229(-0.576,0.650) | 0.212(-0.848,2.380) |
|  |  | *P* value | 0.460 | 0.740 | 0.490 | 0.550 |  | 0.370 | 0.670 | 0.470 | 0.490 |
| T+HTN- | ICAM | Coefficient (95%CI) | 0.018(-0.135,0.110) | 0.130(-0.124,0.490) | 0.148(-0.094,0.470) | 0.123(-1.853,1.800) |  | 0.040(-0.088,0.150) | 0.130(-0.100,0.380) | 0.170(-0.044,0.410) | 0.237(-2.228,2.990) |
|  |  | *P* value | 0.860 | 0.300 | 0.250 | 0.870 |  | 0.520 | 0.270 | 0.130 | 0.600 |
|  | VCAM | Coefficient (95%CI) | 0.024(-0.060,0.140) | 0.124(-0.117,0.450) | 0.148(-0.093,0.470) | 0.162(-1.517,2.540) |  | 0.037(-0.040,0.140) | 0.133(-0.101,0.380) | 0.170(-0.045,0.420) | 0.220(-1.609,1.720) |
|  |  | *P* value | 0.560 | 0.370 | 0.250 | 0.600 |  | 0.310 | 0.230 | 0.130 | 0.380 |
|  | sTNFR1 | Coefficient (95%CI) | 0.028(-0.163,0.160) | 0.120(-0.155,0.520) | 0.148(-0.103,0.450) | 0.188(-2.549,4.270) |  | 0.063( -0.073,0.240) | 0.107(-0.142,0.400) | 0.170(-0.057,0.410) | 0.370(-1.072,3.350) |
|  |  | *P* value | 0.610 | 0.400 | 0.200 | 0.720 |  | 0.240 | 0.410 | 0.110 | 0.330 |
|  | sTNFR2 | Coefficient (95%CI) | 0.044(-0.134,0.180) | 0.105(-0.150,0.460) | 0.148(-0.092,0.450) | 0.295(-2.616,3.740) |  | 0.090(-0.028,0.230) | 0.081(-0.169,0.330) | 0.170(-0.038,0.410) | 0.527(-3.429,4.400) |
|  |  | *P* value | 0.480 | 0.440 | 0.250 | 0.590 |  | 0.130 | 0.480 | 0.140 | 0.270 |
| T+HTN+ | ICAM | Coefficient (95%CI) | 0.047(-0.089,0.190) | 0.547(0.168,0.810) | 0.595(0.168,0.890) | 0.080(-0.285,0.320) |  | 0.051(-0.085,0.170) | 0.480(0.119,0.700) | 0.530(0.110,0.780) | 0.095(-0.438,0.330) |
|  |  | *P* value | 0.478 | 0.012 | 0.012 | 0.466 |  | 0.500 | 0.004 | 0.010 | 0.490 |
|  | VCAM | Coefficient (95%CI) | 0.104(-0.019,0.270) | 0.491(0.068,0.790) | 0.595(0.144,0.900) | 0.174(-0.047,0.560) |  | 0.135(-0.001,0.310) | 0.395(0.036,0.660) | 0.530(0.097,0.800) | 0.254(-0.016,0.770) |
|  |  | *P* value | 0.114 | 0.030 | 0.014 | 0.124 |  | 0.056 | 0.036 | 0.014 | 0.058 |
|  | sTNFR1 | Coefficient (95%CI) | 0.281(0.006,0.510) | 0.314(0.030,0.590) | 0.595(0.147,0.870) | 0.472(0.082,0.900) |  | 0.285(0.021,0.500) | 0.245(0.003,0.480) | 0.530(0.124,0.810) | 0.538(0.129,1.010) |
|  |  | *P* value | 0.042 | 0.032 | 0.016 | **0.030** |  | 0.042 | 0.046 | 0.008 | **0.034** |
|  | sTNFR2 | Coefficient (95%CI) | 0.293(0.062,0.530) | 0.302(-0.086,0.610) | 0.595(0.139,0.880) | 0.492(0.147,1.200) |  | 0.309(0.111,0.530) | 0.221(-0.085,0.490) | 0.530(0.141,0.790) | 0.583(0.299,1.420) |
|  |  | *P* value | 0.014 | 0.106 | 0.012 | **0.018** |  | 0.004 | 0.164 | 0.006 | **0.010** |

The mediation analyses were adjusted to age, sex, years of education and apolipoprotein *E* (*APOE*) *ɛ4* status.

Significance at the level of *P* < 0.05 were shown in bold.

Abbreviations: CSF, cerebrospinal fluid; T-HTN-: T negative and normotension; T-HTN+: T positive and hypertension; T+HTN-: T positive and normotension; T+HTN+: T positive and hypertension; CI, confidence interval; p-tau, phosphorylated tau; t-tau, total tau; sTNFR, soluble tumor necrosis factor receptor; ICAM1, intercellular cell adhesion molecule-1; VCAM1, vascular cell adhesion molecule-1.

**Table S10** Mediation effects of CSF neuroinflammatory biomarkers stratified by A status.

|  | | | p-tau | | | |  | t-tau | | | |
| --- | --- | --- | --- | --- | --- | --- | --- | --- | --- | --- | --- |
|  |  |  | Indirect effect | Direct effect | Total effect | Proportion Mediated |  | Indirect effect | Direct effect | Total effect | Proportion Mediated |
| A- | ICAM | Coefficient (95%CI) | 0.165(-0.003,0.330) | 0.310(-0.111,0.650) | 0.476(-0.022,0.820) | 0.348(-0.356,1.550) |  | 0.164(-0.002,0.350) | 0.284(-0.141,0.600) | 0.448(-0.015,0.760) | 0.366(-0.724,1.460) |
|  |  | *P* value | 0.056 | 0.172 | 0.074 | 0.102 |  | 0.058 | 0.192 | 0.066 | 0.112 |
|  | VCAM | Coefficient (95%CI) | 0.277(0.005,0.510) | 0.199(-0.163,0.470) | 0.476(-0.080,0.820) | 0.582(-0.854,2.300) |  | 0.277(0.021,0.520) | 0.171(-0.168,0.410) | 0.448(-0.009,0.780) | 0.619(-0.132,2.410) |
|  |  | *P* value | 0.042 | 0.324 | 0.096 | 0.098 |  | 0.028 | 0.358 | 0.060 | 0.056 |
|  | sTNFR1 | Coefficient (95%CI) | 0.283(-0.015,0.540) | 0.193(-0.161,0.470) | 0.476(-0.047,0.850) | 0.595(-1.768,1.560) |  | 0.280(-0.020,0.530) | 0.168(-0.149,0.430) | 0.448(-0.043,0.770) | 0.625(-0.104,2.150) |
|  |  | *P* value | 0.062 | 0.312 | 0.094 | 0.112 |  | 0.070 | 0.264 | 0.066 | 0.072 |
|  | sTNFR2 | Coefficient (95%CI) | 0.059(-0.007,0.570) | 0.417(-0.166,0.760) | 0.476(-0.026,0.830) | 0.124(-0.098,1.620) |  | 0.061(-0.012,0.560) | 0.388(-0.125,0.700) | 0.448(-0.006,0.760) | 0.135(-0.117,1.620) |
|  |  | *P* value | 0.190 | 0.250 | 0.070 | 0.210 |  | 0.220 | 0.230 | 0.060 | 0.210 |
| A+ | ICAM | Coefficient (95%CI) | 0.041(-0.018,0.130) | 0.525(0.291,0.740) | 0.566(0.312,0.790) | 0.072(-0.053,0.220) |  | 0.039(-0.0164,0.130) | 0.494(0.311,0.670) | 0.533(0.337,0.720) | 0.073(-0.038,0.240) |
|  |  | *P* value | 0.250 | <2e-16 | <2e-16 | 0.250 |  | 0.190 | <2e-16 | <2e-16 | 0.190 |
|  | VCAM | Coefficient (95%CI) | 0.089(-0.007,0.220) | 0.477(0.226,0.730) | 0.565(0.334,0.780) | 0.157(-0.012,0.400) |  | 0.087(-0.002,0.200) | 0.447(0.229,0.640) | 0.533(0.331,0.710) | 0.163(-0.005,0.390) |
|  |  | *P* value | 0.082 | <2e-16 | <2e-16 | 0.082 |  | 0.058 | <2e-16 | <2e-16 | 0.058 |
|  | sTNFR1 | Coefficient (95%CI) | 0.316(0.148,0.510) | 0.249(0.030,0.460) | 0.566(0.307,0.780) | 0.559(0.308,0.940) |  | 0.280(0.134,0.440) | 0.253(0.053,0.430) | 0.533(0.329,0.710) | 0.525(0.272,0.870) |
|  |  | *P* value | <2e-16 | 0.028 | <2e-16 | **<2e-16** |  | <2e-16 | 0.010 | <2e-16 | **<2e-16** |
|  | sTNFR2 | Coefficient (95%CI) | 0.338(0.172,0.570) | 0.228(-0.036,0.450) | 0.566(0.349,0.800) | 0.598(0.333,1.080) |  | 0.304(0.164,0.480) | 0.229(0.025,0.410) | 0.533(0.350,0.720) | 0.571(0.351,0.950) |
|  |  | *P* value | <2e-16 | 0.088 | <2e-16 | **<2e-16** |  | <2e-16 | 0.036 | <2e-16 | **<2e-16** |

The mediation analyses were adjusted to age, sex, years of education and apolipoprotein *E* (*APOE*) *ɛ4* status.

Significance at the level of *P* < 0.05 were shown in bold.

Abbreviations: CSF, cerebrospinal fluid; A-: A negative; A+: A positive; CI, confidence interval; p-tau, phosphorylated tau; t-tau, total tau; sTNFR, soluble tumor necrosis factor receptor; ICAM1, intercellular cell adhesion molecule-1; VCAM1, vascular cell adhesion molecule-1.

**Table S11** Mediation effects of CSF neuroinflammatory biomarkers stratified by A status and hypertensive status.

|  | | | p-tau | | | |  | t-tau | | | |
| --- | --- | --- | --- | --- | --- | --- | --- | --- | --- | --- | --- |
|  |  |  | Indirect effect | Direct effect | Total effect | Proportion Mediated |  | Indirect effect | Direct effect | Total effect | Proportion Mediated |
| A-HTN- | ICAM | Coefficient (95%CI) | -0.024(-0.373,0.470) | 0.421(-0.070,0.830) | 0.397(-0.142,0.950) | -0.061(-6.191,5.090) |  | -0.021(-0.396,0.390) | 0.405(-0.156,0.910) | 0.384(-0.052,0.860) | -0.055(-3.803,1.420) |
|  |  | *P* value | 0.792 | 0.072 | 0.166 | 0.942 |  | 0.804 | 0.100 | 0.094 | 0.822 |
|  | VCAM | Coefficient (95%CI) | 0.309(-0.009,0.920) | 0.088(-0.313,0.530) | 0.397(-0.119,0.930) | 0.779(-1.985,4.440) |  | 0.311(0.024,0.910) | 0.073(-0.296,0.360) | 0.384(-0.058,0.870) | 0.810(-0.208,2.600) |
|  |  | *P* value | 0.058 | 0.802 | 0.146 | 0.136 |  | 0.034 | 0.650 | 0.076 | 0.066 |
|  | sTNFR1 | Coefficient (95%CI) | 0.281(-0.061,0.910) | 0.116(-0.575,0.630) | 0.397(-0.118,0.930) | 0.708(-4.112,10.060) |  | 0.297(-0.053,0.780) | 0.087(-0.468,0.570) | 0.384(-0.091,0.870) | 0.774(-1.790,4.880) |
|  |  | *P* value | 0.094 | 0.712 | 0.150 | 0.224 |  | 0.084 | 0.682 | 0.088 | 0.128 |
|  | sTNFR2 | Coefficient (95%CI) | 0.237(-0.089,0.760) | 0.160(-0.282,0.640) | 0.397(-0.112,0.910) | 0.598(-4.489,5.860) |  | 0.234(-0.047,0.880) | 0.150(-0.273,0.550) | 0.384(-0.017,0.920) | 0.610(-0.536,3.570) |
|  |  | *P* value | 0.140 | 0.660 | 0.170 | 0.220 |  | 0.094 | 0.582 | 0.070 | 0.108 |
| A-HTN+ | ICAM | Coefficient (95%CI) | 0.266(-0.049,0.600) | 0.390(-0.281,0.890) | 0.656(-0.121,1.150) | 0.406(-0.712,2.310) |  | 0.273(-0.054,0.630) | 0.290(-0.360,0.780) | 0.564(-0.209,1.060) | 0.485(-1.691,2.500) |
|  |  | *P* value | 0.120 | 0.220 | 0.110 | 0.150 |  | 0.110 | 0.370 | 0.160 | 0.160 |
|  | VCAM | Coefficient (95%CI) | 0.320(-0.105,0.770) | 0.336(-0.276,0.760) | 0.656(-0.156,1.160) | 0.487(-0.562,1.960) |  | 0.334(-0.158,0.790) | 0.230(-0.320,0.740) | 0.564(-0.248,1.120) | 0.593(-1.124,2.510) |
|  |  | *P* value | 0.170 | 0.280 | 0.100 | 0.140 |  | 0.170 | 0.460 | 0.160 | 0.150 |
|  | sTNFR1 | Coefficient (95%CI) | 0.281(-0.275,0.650) | 0.375(-0.146,0.870) | 0.656(-0.186,1.190) | 0.428(-1.162,2.050) |  | 0.281(-0.249,0.650) | 0.283(-0.222,0.770) | 0.564(-0.223,1.080) | 0.499(-1.351,2.210) |
|  |  | *P* value | 0.280 | 0.130 | 0.120 | 0.200 |  | 0.270 | 0.200 | 0.160 | 0.230 |
|  | sTNFR2 | Coefficient (95%CI) | 0.042(-0.031,0.720) | 0.614(-0.264,1.050) | 0.656(-0.258,1.130) | 0.065(-0.344,1.660) |  | 0.044(-0.026,0.760) | 0.520(-0.307,1.010) | 0.564(-0.165,1.060) | 0.078(-0.401,2.180) |
|  |  | *P* value | 0.310 | 0.270 | 0.140 | 0.350 |  | 0.280 | 0.430 | 0.160 | 0.320 |
| A+HTN- | ICAM | Coefficient (95%CI) | 0.032(-0.129,0.220) | 0.528(0.127,1.020) | 0.559(0.158,1.020) | 0.057(-0.253,0.390) |  | 0.025(-0.125,0.210) | 0.500(0.137,0.920) | 0.526(0.154,0.950) | 0.048(-0.251,0.410) |
|  |  | *P* value | 0.692 | 0.008 | 0.002 | 0.694 |  | 0.720 | 0.008 | 0.004 | 0.720 |
|  | VCAM | Coefficient (95%CI) | 0.093(-0.085,0.390) | 0.466(0.003,0.990) | 0.559(0.120,1.050) | 0.167(-0.207,0.970) |  | 0.093(-0.044,0.330) | 0.433(0.039,0.840) | 0.526(0.196,0.930) | 0.176(-0.096,0.800) |
|  |  | *P* value | 0.336 | 0.046 | 0.004 | 0.340 |  | 0.208 | 0.028 | 0.006 | 0.210 |
|  | sTNFR1 | Coefficient (95%CI) | 0.206(-0.019,0.510) | 0.353(-0.037,0.850) | 0.559(0.140,1.020) | 0.369(-0.044,1.170) |  | 0.212(0.007,0.480) | 0.314(-0.017,0.720) | 0.525(0.194,0.950) | 0.403(0.022,1.060) |
|  |  | *P* value | 0.066 | 0.082 | 0.004 | 0.070 |  | 0.038 | 0.058 | <2e-16 | 0.038 |
|  | sTNFR2 | Coefficient (95%CI) | 0.291(0.029,0.630) | 0.268(-0.086,0.760) | 0.559(0.154,1.040) | 0.521(0.070,1.310) |  | 0.286(0.022,0.560) | 0.239(-0.051,0.630) | 0.526(0.188,0.900) | 0.545(0.049,1.180) |
|  |  | *P* value | 0.024 | 0.134 | 0.004 | **0.028** |  | 0.030 | 0.108 | 0.004 | **0.034** |
| A+HTN+ | ICAM | Coefficient (95%CI) | 0.046(-0.110,0.180) | 0.537(0.103,0.890) | 0.583(0.099,0.920) | 0.078(-0.444,0.380) |  | 0.045(-0.121,0.160) | 0.507(0.200,0.800) | 0.552(0.176,0.830) | 0.082(-0.342,0.320) |
|  |  | *P* value | 0.544 | 0.026 | 0.026 | 0.534 |  | 0.454 | 0.010 | 0.014 | 0.448 |
|  | VCAM | Coefficient (95%CI) | 0.117(-0.051,0.380) | 0.466(-0.094,0.850) | 0.583(0.111,0.910) | 0.201(-0.141,1.270) |  | 0.117(-0.050,0.380) | 0.436(-0.0003,0.700) | 0.552(0.151,0.790) | 0.211(-0.141,0.960) |
|  |  | *P* value | 0.140 | 0.098 | 0.026 | 0.166 |  | 0.162 | 0.052 | 0.010 | 0.168 |
|  | sTNFR1 | Coefficient (95%CI) | 0.375(0.087,0.840) | 0.208(-0.282,0.550) | 0.583(0.135,0.920) | 0.644(0.181,1.890) |  | 0.306(0.051,0.640) | 0.246(-0.156,0.500) | 0.552(0.176,0.830) | 0.555(0.152,1.520) |
|  |  | *P* value | 0.010 | 0.302 | 0.020 | **0.018** |  | 0.024 | 0.176 | 0.012 | **0.024** |
|  | sTNFR2 | Coefficient (95%CI) | 0.303(0.082,0.690) | 0.280(-0.249,0.680) | 0.583(0.110,0.950) | 0.520(0.056,1.670) |  | 0.259(0.063,0.550) | 0.293(-0.123,0.590) | 0.552(0.195,0.820) | 0.469(0.109,1.410) |
|  |  | *P* value | 0.022 | 0.306 | 0.030 | **0.044** |  | 0.012 | 0.168 | 0.012 | **0.024** |

The mediation analyses were adjusted to age, sex, years of education and apolipoprotein *E* (*APOE*) *ɛ4* status.

Significance at the level of *P* < 0.05 were shown in bold.

Abbreviations: CSF, cerebrospinal fluid; A-HTN-: A negative and normotension; A-HTN+: A positive and hypertension; A+HTN-: A positive and normotension; A+HTN+: A positive and hypertension; CI, confidence interval; p-tau, phosphorylated tau; t-tau, total tau; sTNFR, soluble tumor necrosis factor receptor; ICAM1, intercellular cell adhesion molecule-1; VCAM1, vascular cell adhesion molecule-1.

**Table S12** Mediation effects of CSF neuroinflammatory biomarkers stratified by A status and clinical diagnosis.

|  | | | p-tau | | | |  | t-tau | | | |
| --- | --- | --- | --- | --- | --- | --- | --- | --- | --- | --- | --- |
|  |  |  | Indirect effect | Direct effect | Total effect | Proportion Mediated |  | Indirect effect | Direct effect | Total effect | Proportion Mediated |
| A-CN | ICAM | Coefficient (95%CI) | 0.104(-0.058,0.370) | -0.009(-0.429,0.550) | 0.095(-0.336,0.630) | 1.097(-8.179,6.330) |  | 0.107(-0.081,0.400) | -0.018(-0.457,0.460) | 0.090(-0.306,0.560) | 1.197(-9.151,6.240) |
|  |  | *P* value | 0.250 | 0.970 | 0.630 | 0.660 |  | 0.240 | 0.950 | 0.600 | 0.620 |
|  | VCAM | Coefficient (95%CI) | 0.260(-0.021,0.700) | -0.164(-0.458,0.180) | 0.095(-0.337,0.620) | 2.726(-10.984,18.850) |  | 0.270(-0.044,0.680) | -0.181(-0.425,0.090) | 0.090(-0.309,0.580) | 3.020(-15.613,16.030) |
|  |  | *P* value | 0.074 | 0.296 | 0.612 | 0.538 |  | 0.090 | 0.270 | 0.630 | 0.540 |
|  | sTNFR1 | Coefficient (95%CI) | 0.124(-0.192,0.500) | -0.029(-0.401,0.410) | 0.095(-0.335,0.610) | 1.306(-6.249,6.390) |  | 0.131(-0.215,0.530) | -0.041(-0.359,0.390) | 0.090(-0.318,0.570) | 1.460(-5.018,7.650) |
|  |  | *P* value | 0.380 | 0.980 | 0.620 | 0.500 |  | 0.420 | 0.980 | 0.630 | 0.470 |
|  | sTNFR2 | Coefficient (95%CI) | -0.002(-0.079,0.340) | 0.097(-0.390,0.580) | 0.095(-0.374,0.590) | -0.018(-2.942,4.160) |  | -0.005(-0.061,0.370) | 0.095(-0.329,0.530) | 0.089(-0.285,0.560) | -0.058(-2.446,4.210) |
|  |  | *P* value | 0.470 | 0.870 | 0.640 | 0.780 |  | 0.650 | 0.790 | 0.610 | 0.810 |
| A+CN | ICAM | Coefficient (95%CI) | 0.018(-2.450,3.360) | 0.646(-3.824,2.870) | 0.663(-2.234,1.720) | 0.027(-6.476,7.170) |  | 0.018(-2.343,2.440) | 0.575(-2.899,2.560) | 0.593(-1.451,1.400) | 0.031(-6.537,8.890) |
|  |  | *P* value | 0.840 | 0.620 | 0.420 | 0.990 |  | 0.770 | 0.550 | 0.360 | 0.880 |
|  | VCAM | Coefficient (95%CI) | 0.017(-4.656,3.050) | 0.646(-4.055,5.090) | 0.663(-2.024,1.870) | 0.026(-14.022,7.350) |  | 0.081(-4.185,2.110) | 0.512(-2.328,5.240) | 0.593(-1.483,1.620) | 0.136(-6.896,8.090) |
|  |  | *P* value | 0.960 | 0.550 | 0.410 | 0.970 |  | 0.970 | 0.510 | 0.400 | 0.520 |
|  | sTNFR1 | Coefficient (95%CI) | 0.421(-3.234,3.950) | 0.242(-4.584,4.480) | 0.663(-2.187,2.030) | 0.635(-6.674,12.550) |  | 0.329(-3.333,2.660) | 0.264(-2.448,3.760) | 0.593(-1.813,1.530) | 0.555(-8.501,7.610) |
|  |  | *P* value | 0.550 | 0.960 | 0.400 | 0.650 |  | 0.570 | 0.840 | 0.370 | 0.610 |
|  | sTNFR2 | Coefficient (95%CI) | 0.015(-4.351,4.700) | 0.649(-4.54,4.670) | 0.663(-2.077,2.090) | 0.022(-13.834,9.520) |  | 0.050(-4.125,2.630) | 0.543(-2.843,4.680) | 0.593(-1.781,1.510) | 0.084(-9.833,5.980) |
|  |  | *P* value | 0.860 | 0.490 | 0.400 | 0.920 |  | 0.950 | 0.530 | 0.400 | 0.990 |
| A-MCI | ICAM | Coefficient (95%CI) | 0.035(-1.273,0.850) | 0.431(-1.195,1.230) | 0.466(-1.701,1.280) | 0.074(-1.726,3.920) |  | 0.033(-0.967,1.020) | 0.490(-1.178,1.110) | 0.523(-1.477,1.270) | 0.064(-5.980,7.780) |
|  |  | *P* value | 0.950 | 0.620 | 0.670 | 0.530 |  | 0.940 | 0.450 | 0.550 | 0.580 |
|  | VCAM | Coefficient (95%CI) | 0.351(-0.955,1.690) | 0.115(-1.435,0.780) | 0.466(-1.483,1.290) | 0.753(-2.922,3.470) |  | 0.350(-0.971,1.390) | 0.173(-1.236,0.810) | 0.523(-1.680,1.330) | 0.669(-2.250,4.220) |
|  |  | *P* value | 0.480 | 0.960 | 0.660 | 0.350 |  | 0.490 | 0.810 | 0.560 | 0.270 |
|  | sTNFR1 | Coefficient (95%CI) | 0.313(-0.904,1.130) | 0.153(-1.152,1.100) | 0.466(-1.397,1.400) | 0.672(-1.171,2.760) |  | 0.327(-0.936,0.950) | 0.196(-1.132,0.890) | 0.523(-1.794,1.300) | 0.625(-1.472,2.760) |
|  |  | *P* value | 0.610 | 0.800 | 0.640 | 0.260 |  | 0.620 | 0.690 | 0.580 | 0.220 |
|  | sTNFR2 | Coefficient (95%CI) | 0.327(-1.288,1.190) | 0.139(-0.953,0.810) | 0.466(-1.212,1.460) | 0.701(-2.063,4.130) |  | 0.329(-1.074,1.220) | 0.194(-0.867,0.650) | 0.523(-1.337,1.290) | 0.630(-1.894,6.120) |
|  |  | *P* value | 0.510 | 0.970 | 0.700 | 0.320 |  | 0.430 | 0.790 | 0.550 | 0.220 |
| A+MCI | ICAM | Coefficient (95%CI) | 0.063(-0.027,0.230) | 0.448(0.131,0.770) | 0.511(0.184,0.830) | 0.123(-0.088,0.490) |  | 0.055(-0.033,0.180) | 0.437(0.199,0.680) | 0.492(0.240,0.720) | 0.111(-0.081,0.390) |
|  |  | *P* value | 0.220 | 0.004 | 0.002 | 0.222 |  | 0.270 | <2e-16 | <2e-16 | 0.270 |
|  | VCAM | Coefficient (95%CI) | 0.130(-0.030,0.350) | 0.382(0.010,0.730) | 0.511(0.196,0.800) | 0.254(-0.071,0.960) |  | 0.117(-0.025,0.300) | 0.375(0.055,0.660) | 0.492(0.219,0.750) | 0.238(-0.055,0.810) |
|  |  | *P* value | 0.140 | 0.044 | 0.002 | 0.142 |  | 0.112 | <2e-16 | <2e-16 | 0.112 |
|  | sTNFR1 | Coefficient (95%CI) | 0.341(0.091,0.680) | 0.170(-0.239,0.530) | 0.511(0.177,0.810) | 0.667(0.227,1.800) |  | 0.304(0.096,0.600) | 0.188(-0.171,0.460) | 0.492(0.240,0.730) | 0.617(0.206,1.470) |
|  |  | *P* value | 0.010 | 0.334 | 0.002 | **0.012** |  | 0.006 | 0.214 | <2e-16 | **0.006** |
|  | sTNFR2 | Coefficient (95%CI) | 0.487(0.182,0.860) | 0.024(-0.381,0.400) | 0.511(0.171,0.800) | 0.953(0.388,2.430) |  | 0.415(0.177,0.690) | 0.077(-0.234,0.360) | 0.492(0.245,0.740) | 0.843(0.390,1.690) |
|  |  | *P* value | 0.002 | 0.980 | 0.006 | **0.004** |  | <2e-16 | 0.690 | <2e-16 | **<2e-16** |

The mediation analyses were adjusted to age, sex, years of education and apolipoprotein *E* (*APOE*) *ɛ4* status.

Significance at the level of *P* < 0.05 were shown in bold.

Abbreviations: CSF, cerebrospinal fluid; A-CN: A negative and cognitively normal; A+CN: A positive and cognitively normal; A-MCI: A negative and mild cognitive impairment; A+MCI: A positive and mild cognitive impairment; CI, confidence interval; p-tau, phosphorylated tau; t-tau, total tau; sTNFR, soluble tumor necrosis factor receptor; ICAM1, intercellular cell adhesion molecule-1; VCAM1, vascular cell adhesion molecule-1.

**Table S13** Baseline CSF YKL-40 and longitudinal changes in cognition and MRI brain structures stratified by clinical diagnosis.

| CN |  |  | MMSE | | | | |  | FAQ | | | | |  | ADAS13 | | | | |  | \ | | | | |
| --- | --- | --- | --- | --- | --- | --- | --- | --- | --- | --- | --- | --- | --- | --- | --- | --- | --- | --- | --- | --- | --- | --- | --- | --- | --- |
|  | Predictors |  | Coefficients |  | SE |  | *P* value |  | Coefficients |  | SE |  | *P* value |  | Coefficients |  | SE |  | *P* value |  | Coefficients |  | SE |  | *P* value |
|  | Age × time |  | -0.017 |  | 0.006 |  | 0.004 |  | 0.026 |  | 0.011 |  | 0.022 |  | 0.029 |  | 0.015 |  | 0.047 |  | \ |  | \ |  | \ |
|  | Female × time |  | 0.128 |  | 0.066 |  | 0.053 |  | -0.184 |  | 0.123 |  | 0.136 |  | -0.449 |  | 0.155 |  | 0.004 |  | \ |  | \ |  | \ |
|  | Education × time |  | -0.022 |  | 0.009 |  | 0.010 |  | 0.014 |  | 0.016 |  | 0.376 |  | 0.026 |  | 0.020 |  | 0.199 |  | \ |  | \ |  | \ |
|  | *APOE ɛ4* × time |  | -0.013 |  | 0.078 |  | 0.867 |  | 0.310 |  | 0.144 |  | 0.032 |  | 0.478 |  | 0.178 |  | 0.008 |  | \ |  | \ |  | \ |
|  | Group high × time |  | 0.160 |  | 0.069 |  | **0.021** |  | -0.206 |  | 0.129 |  | 0.111 |  | -0.356 |  | 0.166 |  | **0.033** |  | \ |  | \ |  | \ |
|  |  |  | MEM | | | | |  | LAN | | | | |  | EF | | | | |  | VP | | | | |
|  | Predictors |  | Coefficients |  | SE |  | *P* value |  | Coefficients |  | SE |  | *P* value |  | Coefficients |  | SE |  | *P* value |  | Coefficients |  | SE |  | *P* value |
|  | Age × time |  | -0.001 |  | 0.001 |  | 0.432 |  | 0.002 |  | 0.001 |  | 0.076 |  | 0.000 |  | 0.001 |  | 0.679 |  | 0.000 |  | 0.001 |  | 0.991 |
|  | Female × time |  | 0.035 |  | 0.012 |  | 0.005 |  | 0.029 |  | 0.012 |  | 0.020 |  | 0.038 |  | 0.012 |  | 0.002 |  | -0.017 |  | 0.017 |  | 0.315 |
|  | Education × time |  | -0.001 |  | 0.002 |  | 0.563 |  | -0.002 |  | 0.002 |  | 0.254 |  | -0.005 |  | 0.002 |  | 0.001 |  | -0.004 |  | 0.002 |  | 0.043 |
|  | *APOE ɛ4* × time |  | -0.022 |  | 0.013 |  | 0.097 |  | -0.006 |  | 0.013 |  | 0.637 |  | 0.004 |  | 0.013 |  | 0.751 |  | -0.016 |  | 0.017 |  | 0.347 |
|  | Group high × time |  | -0.021 |  | 0.013 |  | 0.104 |  | -0.015 |  | 0.013 |  | 0.262 |  | 0.042 |  | 0.013 |  | **0.002** |  | 0.019 |  | 0.022 |  | 0.393 |
|  |  |  | Hippocampus | | | | |  | Entorhinal cortex | | | | |  | Mid temporal lobe | | | | |  | Whole brain | | | | |
|  | Predictors |  | Coefficients |  | SE |  | *P* value |  | Coefficients |  | SE |  | *P* value |  | Coefficients |  | SE |  | *P* value |  | Coefficients |  | SE |  | *P* value |
|  | Age × time |  | -1.319e-04 |  | 9.549e-05 |  | 0.170 |  | -9.639eE-05 |  | 1.119e-04 |  | 0.391 |  | -7.233e-06 |  | 7.100e-05 |  | 0.919 |  | 1.989e-05 |  | 4.102e-05 |  | 0.629 |
|  | Female × time |  | 4.642e-03 |  | 1.142e-03 |  | 8.380e-05 |  | 5.738e-03 |  | 1.339e-03 |  | 3.560e-05 |  | 3.795e-04 |  | 8.494e-04 |  | 0.656 |  | 1.922e-04 |  | 4.907e-04 |  | 0.696 |
|  | Education × time |  | -7.314e-04 |  | 1.729e-04 |  | 4.400e-05 |  | -7.329e-04 |  | 2.026e-04 |  | 4.280e-04 |  | -3.354e-04 |  | 1.285e-04 |  | 0.010 |  | -1.561e-04 |  | 7.425e-05 |  | 0.038 |
|  | *APOE ɛ4* × time |  | -5.116e-03 |  | 1.100e-03 |  | 8.070e-06 |  | -6.170e-03 |  | 1.289e-03 |  | 4.590e-06 |  | -1.015e-03 |  | 8.176e-04 |  | 0.217 |  | -9.269e-05 |  | 4.723e-04 |  | 0.845 |
|  | ICV × time |  | -4.542e-09 |  | 4.194e-09 |  | 0.281 |  | -2.271e-09 |  | 4.916e-09 |  | 0.645 |  | 2.032e-09 |  | 3.118e-09 |  | 0.516 |  | -1.342e-09 |  | 1.801e-09 |  | 0.458 |
|  | Group high × time |  | -7.316e-04 |  | 9.561e-04 |  | 0.446 |  | -3.777e-04 |  | 1.121e-03 |  | 0.737 |  | -1.526e-03 |  | 7.109e-04 |  | **0.034** |  | 1.699e-04 |  | 4.107e-04 |  | 0.680 |
| MCI |  |  | MMSE | | | | |  | FAQ | | | | |  | ADAS13 | | | | |  | \ | | | | |
|  | Predictors |  | Coefficients |  | SE |  | *P* value |  | Coefficients |  | SE |  | *P* value |  | Coefficients |  | SE |  | *P* value |  | Coefficients |  | SE |  | *P* value |
|  | Age × time |  | -5.306e-03 |  | 8.133e-03 |  | 0.515 |  | 0.035 |  | 0.013 |  | 0.010 |  | -0.022 |  | 0.019 |  | 0.242 |  | \ |  | \ |  | \ |
|  | Female × time |  | 0.082 |  | 0.139 |  | 0.556 |  | 0.876 |  | 0.230 |  | 1.660e-4 |  | -0.296 |  | 0.306 |  | 0.334 |  | \ |  | \ |  | \ |
|  | Education × time |  | -0.014 |  | 0.028 |  | 0.608 |  | -0.097 |  | 0.046 |  | 0.035 |  | 0.008 |  | 0.059 |  | 0.888 |  | \ |  | \ |  | \ |
|  | *APOE ɛ4* × time |  | -1.023 |  | 0.110 |  | < 2e-16 |  | 2.233 |  | 0.182 |  | < 2e-16 |  | 2.254 |  | 0.253 |  | <2e-16 |  | \ |  | \ |  | \ |
|  | Group high × time |  | -0.348 |  | 0.121 |  | **4.318e-03** |  | -0.034 |  | 0.200 |  | 0.865 |  | 0.482 |  | 0.273 |  | 0.079 |  | \ |  | \ |  | \ |
|  |  |  | MEM | | | | |  | LAN | | | | |  | EF | | | | |  | VP | | | | |
|  | Predictors |  | Coefficients |  | SE |  | *P* value |  | Coefficients |  | SE |  | *P* value |  | Coefficients |  | SE |  | *P* value |  | Coefficients |  | SE |  | *P* value |
|  | Age × time |  | -2.341e-03 |  | 7.738e-04 |  | 2.660e-03 |  | -0.002 |  | 0.001 |  | 0.135 |  | -3.240e-04 |  | 1.210e-03 |  | 0.789 |  | 5.352e-03 |  | 2.265e-03 |  | 0.019 |
|  | Female × time |  | -9.437e-03 |  | 0.013 |  | 0.459 |  | 0.041 |  | 0.017 |  | 0.013 |  | 0.043 |  | 0.020 |  | 0.032 |  | 9.691e-03 |  | 0.036 |  | 0.787 |
|  | Education × time |  | -3.129e-04 |  | 2.518e-03 |  | 0.901 |  | -0.004 |  | 0.003 |  | 0.214 |  | -3.093e-03 |  | 3.936e-03 |  | 0.433 |  | -2.449e-03 |  | 7.028e-03 |  | 0.728 |
|  | *APOE ɛ4* × time |  | -0.123 |  | 0.011 |  | < 2e-16 |  | -0.125 |  | 0.014 |  | <2e-16 |  | -0.140 |  | 0.017 |  | 9.960e-16 |  | 5.098e-03 |  | 0.034 |  | 0.883 |
|  | Group high × time |  | -0.014 |  | 0.011 |  | 0.225 |  | -0.011 |  | 0.015 |  | 0.453 |  | -0.024 |  | 0.018 |  | 0.187 |  | -0.106 |  | 0.028 |  | **1.750e-04** |
|  |  |  | Hippocampus | | | | |  | Entorhinal cortex | | | | |  | Mid temporal lobe | | | | |  | Whole brain | | | | |
|  | Predictors |  | Coefficients |  | SE |  | *P* value |  | Coefficients |  | SE |  | *P* value |  | Coefficients |  | SE |  | *P* value |  | Coefficients |  | SE |  | *P* value |
|  | Age × time |  | -1.244e-04 |  | 1.097e-04 |  | 0.258 |  | 8.253e-05 |  | 1.103e-04 |  | 0.455 |  | -8.435e-06 |  | 9.177e-05 |  | 0.927 |  | 5.612e-06 |  | 3.918e-05 |  | 0.886 |
|  | Female × time |  | -1.511e-03 |  | 1.576e-03 |  | 0.339 |  | 2.090e-03 |  | 1.584e-03 |  | 0.188 |  | 3.413e-03 |  | 1.318e-03 |  | 0.010 |  | 1.717e-03 |  | 5.627e-04 |  | 2.590e-03 |
|  | Education × time |  | 4.906e-04 |  | 2.718e-04 |  | 0.073 |  | -1.210e-04 |  | 2.731e-04 |  | 0.658 |  | -2.610e-04 |  | 2.273e-04 |  | 0.252 |  | 4.041e-05 |  | 9.703e-05 |  | 0.678 |
|  | *APOE ɛ4* × time |  | -1.311e-03 |  | 1.415e-03 |  | 0.355 |  | -3.856e-03 |  | 1.422e-03 |  | 7.260e-03 |  | -4.332e-03 |  | 1.183e-03 |  | 3.200e-04 |  | -1.538e-03 |  | 5.051e-04 |  | 2.640e-03 |
|  | ICV × time |  | 8.200e-09 |  | 5.327e-09 |  | 0.125 |  | 1.556e-08 |  | 5.353e-09 |  | 4.050e-03 |  | 2.246e-09 |  | 4.454e-09 |  | 0.615 |  | -2.446e-09 |  | 1.902e-09 |  | 0.200 |
|  | Group high × time |  | -2.363e-03 |  | 1.276e-03 |  | 0.066 |  | -2.392e-03 |  | 1.282e-03 |  | 0.064 |  | -1.023e-03 |  | 1.067e-03 |  | 0.339 |  | -5.825e-04 |  | 4.556e-04 |  | 0.203 |
| AD |  |  | MMSE | | | | |  | FAQ | | | | |  | ADAS13 | | | | |  | \ | | | | |
|  | Predictors |  | Coefficients |  | SE |  | *P* value |  | Coefficients |  | SE |  | *P* value |  | Coefficients |  | SE |  | *P* value |  | Coefficients |  | SE |  | *P* value |
|  | Age × time |  | 0.207 |  | 0.067 |  | 0.003 |  | -0.104 |  | 0.088 |  | 0.241 |  | -0.244 |  | 0.314 |  | 0.441 |  | \ |  | \ |  | \ |
|  | Female × time |  | 0.336 |  | 0.737 |  | 0.650 |  | 2.479 |  | 0.975 |  | 0.014 |  | 8.892 |  | 3.430 |  | 0.012 |  | \ |  | \ |  | \ |
|  | Education × time |  | -0.038 |  | 0.155 |  | 0.806 |  | -0.057 |  | 0.206 |  | 0.781 |  | 0.335 |  | 0.734 |  | 0.649 |  | \ |  | \ |  | \ |
|  | *APOE ɛ4* × time |  | 3.984 |  | 0.928 |  | 0.000 |  | -2.986 |  | 1.228 |  | 0.018 |  | 3.084 |  | 4.354 |  | 0.481 |  | \ |  | \ |  | \ |
|  | Group high × time |  | -1.210 |  | 0.722 |  | 0.990 |  | -1.096 |  | 0.955 |  | 0.256 |  | -3.140 |  | 3.351 |  | 0.352 |  | \ |  | \ |  | \ |
|  |  |  | MEM | | | | |  | LAN | | | | |  | EF | | | | |  | VP | | | | |
|  | Predictors |  | Coefficients |  | SE |  | *P* value |  | Coefficients |  | SE |  | *P* value |  | Coefficients |  | SE |  | *P* value |  | Coefficients |  | SE |  | *P* value |
|  | Age × time |  | 0.019 |  | 5.464e-03 |  | 8.100e-04 |  | 0.024 |  | 9.207E-03 |  | 0.013 |  | 0.026 |  | 7.288E-03 |  | 7.340e-04 |  | 0.034 |  | 9.226e-03 |  | 5.560e-04 |
|  | Female × time |  | -0.036 |  | 0.060 |  | 0.559 |  | 4.220e-03 |  | 0.102 |  | 0.967 |  | 0.023 |  | 0.081 |  | 0.776 |  | 0.315 |  | 0.116 |  | 9.009e-03 |
|  | Education × time |  | -0.001 |  | 0.013 |  | 0.939 |  | -0.030 |  | 0.021 |  | 0.163 |  | -0.060 |  | 0.017 |  | 8.740e-04 |  | -0.039 |  | 0.021 |  | 0.072 |
|  | *APOE ɛ4* × time |  | 0.280 |  | 0.076 |  | 5.550e-04 |  | 0.372 |  | 0.128 |  | 5.460e-03 |  | 0.053 |  | 0.102 |  | 0.605 |  | 0.575 |  | 0.127 |  | 4.260e-05 |
|  | Group high × time |  | -0.045 |  | 0.059 |  | 0.450 |  | -0.145 |  | 0.100 |  | 0.153 |  | -0.010 |  | 0.079 |  | 0.896 |  | -0.270 |  | 0.108 |  | **0.016** |
|  |  |  | Hippocampus | | | | |  | Entorhinal cortex | | | | |  | Mid temporal lobe | | | | |  | Whole brain | | | | |
|  | Predictors |  | Coefficients |  | SE |  | *P* value |  | Coefficients |  | SE |  | *P* value |  | Coefficients |  | SE |  | *P* value |  | Coefficients |  | SE |  | *P* value |
|  | Age × time |  | 2.959e-04 |  | 3.275e-04 |  | 0.372 |  | -6.211e-06 |  | 2.495e-04 |  | 0.980 |  | 6.197e-04 |  | 2.884e-04 |  | 0.038 |  | 2.254e-04 |  | 8.657e-05 |  | 0.013 |
|  | Female × time |  | -9.896e-04 |  | 4.141e-03 |  | 0.812 |  | -5.773e-03 |  | 3.148e-03 |  | 0.075 |  | -1.317e-03 |  | 3.643e-03 |  | 0.720 |  | 1.041e-03 |  | 1.093e-03 |  | 0.347 |
|  | Education × time |  | 2.098e-04 |  | 6.932e-04 |  | 0.764 |  | -4.671e-05 |  | 5.274e-04 |  | 0.930 |  | -2.115e-04 |  | 6.100e-04 |  | 0.731 |  | 2.997e-04 |  | 1.830e-04 |  | 0.110 |
|  | *APOE ɛ4* × time |  | 4.357e-03 |  | 4.382e-03 |  | 0.326 |  | 6.316e-04 |  | 3.338e-03 |  | 0.851 |  | -5.113e-03 |  | 3.859e-03 |  | 0.193 |  | -6.044e-04 |  | 1.158e-03 |  | 0.605 |
|  | ICV × time |  | 2.656e-08 |  | 1.342e-08 |  | 0.055 |  | 3.694e-08 |  | 1.020e-08 |  | 8.520e-04 |  | 3.430e-08 |  | 1.180e-08 |  | 6.062e-03 |  | 8.937e-09 |  | 3.540e-09 |  | 0.016 |
|  | Group high × time |  | -1.105e-03 |  | 4.200e-03 |  | 0.794 |  | 1.840e-03 |  | 3.195e-03 |  | 0.568 |  | 1.702e-03 |  | 3.695e-03 |  | 0.648 |  | 1.126e-06 |  | 1.109e-03 |  | 0.999 |

The primary effects of the predictive factors (i.e., age, sex, years of education, *APOE ɛ4* status, years since baseline, and ICV when appropriate) were incorporated into all linear mixed-effects models, while coefficients are not shown for the sake of brevity.

Abbreviations: CSF, cerebrospinal fluid; CN: cognitively normal; MCI: mild cognitive impairment; AD: Alzheimer’s disease; MMSE, Mini-Mental State Examination; FAQ, Functional Assessment Questionnaire; ADAS13, Alzheimer’s disease Assessment Scale 13; MEM, memory function; LAN, language; EF, executive function; VP, visuospatial functioning; *APOE ε4*, apolipoprotein *E4*; ICV, Intracranial volume; SE, Standard Error; Group high, CSF YKL-40 ≥ 390ng/ml (determined by the median concentration).

**Table S14** Baseline CSF YKL-40 and longitudinal changes in cognition and MRI brain structures stratified by T status.

| T- |  |  | MMSE | | | | |  | FAQ | | | | |  | ADAS13 | | | | |  | \ | | | | |
| --- | --- | --- | --- | --- | --- | --- | --- | --- | --- | --- | --- | --- | --- | --- | --- | --- | --- | --- | --- | --- | --- | --- | --- | --- | --- |
|  | Predictors |  | Coefficients |  | SE |  | *P* value |  | Coefficients |  | SE |  | *P* value |  | Coefficients |  | SE |  | *P* value |  | Coefficients |  | SE |  | *P* value |
|  | Age × time |  | -0.012 |  | 4.498e-03 |  | 7.980e-03 |  | 0.022 |  | 0.011 |  | 0.055 |  | -6.951e-03 |  | 0.015 |  | 0.634 |  | \ |  | \ |  | \ |
|  | Female × time |  | 2.276e-03 |  | 0.072 |  | 0.975 |  | 0.660 |  | 0.179 |  | 2.590e-04 |  | 0.307 |  | 0.205 |  | 0.135 |  | \ |  | \ |  | \ |
|  | Education × time |  | -7.131e-03 |  | 7.496e-03 |  | 0.342 |  | -0.002 |  | 0.019 |  | 0.922 |  | -0.017 |  | 0.021 |  | 0.407 |  | \ |  | \ |  | \ |
|  | *APOE ɛ4* × time |  | -0.130 |  | 0.070 |  | 0.064 |  | 0.296 |  | 0.173 |  | 0.088 |  | 0.527 |  | 0.192 |  | 6.470e-03 |  | \ |  | \ |  | \ |
|  | Group high × time |  | -0.055 |  | 0.063 |  | 0.384 |  | -0.056 |  | 0.156 |  | 0.719 |  | 0.188 |  | 0.184 |  | 0.307 |  | \ |  | \ |  | \ |
|  |  |  | MEM | | | | |  | LAN | | | | |  | EF | | | | |  | VP | | | | |
|  | Predictors |  | Coefficients |  | SE |  | *P* value |  | Coefficients |  | SE |  | *P* value |  | Coefficients |  | SE |  | *P* value |  | Coefficients |  | SE |  | *P* value |
|  | Age × time |  | -1.388e-03 |  | 8.723e-04 |  | 0.113 |  | 6.119e-04 |  | 1.039e-03 |  | 0.556 |  | -2.028e-03 |  | 1.082e-03 |  | 0.062 |  | -3.855e-03 |  | 2.106e-03 |  | 0.069 |
|  | Female × time |  | -6.747e-03 |  | 0.013 |  | 0.600 |  | 0.018 |  | 0.015 |  | 0.231 |  | 0.031 |  | 0.016 |  | 0.052 |  | 0.015 |  | 0.027 |  | 0.594 |
|  | Education × time |  | 2.901e-03 |  | 1.394e-03 |  | 0.038 |  | -5.326e-04 |  | 1.661e-03 |  | 0.749 |  | -2.621e-03 |  | 1.729e-03 |  | 0.131 |  | -2.434e-03 |  | 2.450e-03 |  | 0.322 |
|  | *APOE ɛ4* × time |  | -0.028 |  | 0.012 |  | 0.021 |  | -0.035 |  | 0.014 |  | 0.013 |  | -7.164e-03 |  | 0.015 |  | 0.626 |  | -0.035 |  | 0.023 |  | 0.131 |
|  | Group high × time |  | -0.037 |  | 0.012 |  | **1.320e-03** |  | -0.040 |  | 0.014 |  | **3.640e-03** |  | 0.020 |  | 0.014 |  | 0.173 |  | -0.032 |  | 0.026 |  | 0.217 |
|  |  |  | Hippocampus | | | | |  | Entorhinal cortex | | | | |  | Mid temporal lobe | | | | |  | Whole brain | | | | |
|  | Predictors |  | Coefficients |  | SE |  | *P* value |  | Coefficients |  | SE |  | *P* value |  | Coefficients |  | SE |  | *P* value |  | Coefficients |  | SE |  | *P* value |
|  | Age × time |  | -4.097e-05 |  | 1.246e-04 |  | 0.743 |  | 1.089e-03 |  | 1.681e-03 |  | 0.518 |  | 3.177e-05 |  | 6.227e-05 |  | 0.611 |  | 4.181e-05 |  | 3.465e-05 |  | 0.230 |
|  | Female × time |  | -2.160e-04 |  | 1.913e-03 |  | 0.910 |  | -8.386e-04 |  | 3.045e-04 |  | 6.710E-03 |  | 9.483e-04 |  | 9.561e-04 |  | 0.323 |  | 9.670e-05 |  | 5.319e-04 |  | 0.856 |
|  | Education × time |  | -3.602e-04 |  | 3.464e-04 |  | 0.300 |  | 5.510e-04 |  | 2.143e-03 |  | 0.798 |  | -3.847e-04 |  | 1.732e-04 |  | 0.028 |  | -1.427e-04 |  | 9.634e-05 |  | 0.141 |
|  | *APOE ɛ4* × time |  | 3.223e-03 |  | 2.439e-03 |  | 0.189 |  | 5.547e-09 |  | 5.192e-09 |  | 0.287 |  | 1.555e-04 |  | 1.219e-03 |  | 0.899 |  | 1.704e-04 |  | 6.782e-04 |  | 0.802 |
|  | ICV × time |  | 1.387e-10 |  | 5.907e-09 |  | 0.981 |  | 8.739e-04 |  | 1.365e-03 |  | 0.523 |  | 1.804e-09 |  | 2.953e-09 |  | 0.542 |  | -5.418e-10 |  | 1.643e-09 |  | 0.742 |
|  | Group high × time |  | -2.112e-03 |  | 1.553e-03 |  | 0.176 |  | 1.089e-03 |  | 1.681e-03 |  | 0.518 |  | 3.789e-04 |  | 7.761e-04 |  | 0.626 |  | 7.562e-04 |  | 4.318e-04 |  | 0.082 |
| T+ |  |  | MMSE | | | | |  | FAQ | | | | |  | ADAS13 | | | | |  | \ | | | | |
|  | Predictors |  | Coefficients |  | SE |  | *P* value |  | Coefficients |  | SE |  | *P* value |  | Coefficients |  | SE |  | *P* value |  | Coefficients |  | SE |  | *P* value |
|  | Age × time |  | 0.013 |  | 9.663e-03 |  | 0.176 |  | 9.189e-03 |  | 0.014 |  | 0.521 |  | -0.054 |  | 0.025 |  | 0.029 |  | \ |  | \ |  | \ |
|  | Female × time |  | 0.147 |  | 0.120 |  | 0.219 |  | 0.173 |  | 0.176 |  | 0.326 |  | -0.767 |  | 0.325 |  | 0.019 |  | \ |  | \ |  | \ |
|  | Education × time |  | 0.039 |  | 0.021 |  | 0.067 |  | -0.123 |  | 0.031 |  | 7.300e-05 |  | -2.463e-03 |  | 0.057 |  | 0.965 |  | \ |  | \ |  | \ |
|  | *APOE ɛ4* × time |  | -0.676 |  | 0.108 |  | 8.000e-10 |  | 1.809 |  | 0.158 |  | < 2e-16 |  | 1.617 |  | 0.288 |  | 3.430e-08 |  | \ |  | \ |  | \ |
|  | Group high × time |  | -0.129 |  | 0.106 |  | 0.225 |  | 6.383e-03 |  | 0.156 |  | 0.968 |  | -0.280 |  | 0.276 |  | 0.310 |  | \ |  | \ |  | \ |
|  |  |  | MEM | | | | |  | LAN | | | | |  | EF | | | | |  | VP | | | | |
|  | Predictors |  | Coefficients |  | SE |  | *P* value |  | Coefficients |  | SE |  | *P* value |  | Coefficients |  | SE |  | *P* value |  | Coefficients |  | SE |  | *P* value |
|  | Age × time |  | 7.783e-04 |  | 9.873e-04 |  | 0.431 |  | 2.916e-03 |  | 1.172e-03 |  | 0.013 |  | 4.650e-03 |  | 1.304e-03 |  | 4.030e-04 |  | 8.509e-03 |  | 1.956e-03 |  | 1.840e-05 |
|  | Female × time |  | 0.024 |  | 0.013 |  | 0.068 |  | 0.036 |  | 0.016 |  | 0.023 |  | 0.062 |  | 0.017 |  | 4.070e-04 |  | 1.758e-02 |  | 0.032 |  | 0.585 |
|  | Education × time |  | 2.402e-03 |  | 2.306e-03 |  | 0.298 |  | 3.607e-05 |  | 2.740e-03 |  | 0.990 |  | -3.435e-03 |  | 3.004e-03 |  | 0.253 |  | 2.538e-03 |  | 5.754e-03 |  | 0.659 |
|  | *APOE ɛ4* × time |  | -0.079 |  | 0.012 |  | 5.370e-11 |  | -0.061 |  | 0.014 |  | 1.420e-05 |  | -0.093 |  | 0.015 |  | 2.880e-09 |  | -0.020 |  | 0.025 |  | 0.425 |
|  | Group high × time |  | -4.927e-03 |  | 0.011 |  | 0.655 |  | 8.218e-03 |  | 0.013 |  | 0.530 |  | 0.013 |  | 0.015 |  | 0.392 |  | -0.067 |  | 0.025 |  | **7.766e-03** |
|  |  |  | Hippocampus | | | | |  | Entorhinal cortex | | | | |  | Mid temporal lobe | | | | |  | Whole brain | | | | |
|  | Predictors |  | Coefficients |  | SE |  | *P* value |  | Coefficients |  | SE |  | *P* value |  | Coefficients |  | SE |  | *P* value |  | Coefficients |  | SE |  | *P* value |
|  | Age × time |  | -1.102e-04 |  | 9.106e-05 |  | 0.228 |  | 8.869e-05 |  | 9.634e-05 |  | 0.358 |  | 2.550e-04 |  | 8.760e-05 |  | 3.950e-03 |  | 1.252e-04 |  | 3.502e-05 |  | 4.270e-04 |
|  | Female × time |  | 1.086e-03 |  | 1.337e-03 |  | 0.418 |  | 3.317e-03 |  | 1.414e-03 |  | 0.020 |  | 2.437e-03 |  | 1.286e-03 |  | 5.936e-02 |  | 1.337e-03 |  | 5.142e-04 |  | 9.931e-03 |
|  | Education × time |  | 6.454e-04 |  | 2.194e-04 |  | 3.600e-03 |  | 3.889e-04 |  | 2.321e-04 |  | 0.095 |  | 2.685e-04 |  | 2.111e-04 |  | 0.205 |  | 1.919e-04 |  | 8.439e-05 |  | 0.024 |
|  | *APOE ɛ4* × time |  | -7.334e-03 |  | 1.186e-03 |  | 2.780e-09 |  | -8.350e-03 |  | 1.255e-03 |  | 2.000e-10 |  | -4.683e-03 |  | 1.141e-03 |  | 5.620e-05 |  | -1.137e-03 |  | 4.562e-04 |  | 0.013 |
|  | ICV × time |  | -3.821e-09 |  | 4.919e-09 |  | 0.438 |  | 4.780e-09 |  | 5.203e-09 |  | 0.359 |  | -1.050e-09 |  | 4.732e-09 |  | 0.825 |  | -2.676e-09 |  | 1.891e-09 |  | 0.158 |
|  | Group high × time |  | -1.607e-03 |  | 1.122e-03 |  | 0.154 |  | -3.262e-03 |  | 1.187e-03 |  | **6.480e-03** |  | -2.547e-03 |  | 1.080e-03 |  | **0.019** |  | -1.219e-03 |  | 4.316e-04 |  | **5.153e-03** |

The primary effects of the predictive factors (i.e., age, sex, years of education, *APOE ɛ4* status, years since baseline, and ICV when appropriate) were incorporated into all linear mixed-effects models, while coefficients are not shown for the sake of brevity.

Abbreviations: CSF, cerebrospinal fluid; T-: T negative; T+: T positive; MMSE, Mini-Mental State Examination; FAQ, Functional Assessment Questionnaire; ADAS13, Alzheimer’s disease Assessment Scale 13; MEM, memory function; LAN, language; EF, executive function; VP, visuospatial functioning; *APOE ε4*, apolipoprotein *E4*; ICV, Intracranial volume; SE, Standard Error; Group high, CSF YKL-40 ≥ 390ng/ml (determined by the median concentration).

**Table S15** Baseline CSF YKL-40 and longitudinal changes in cognition and MRI brain structures stratified by T status and hypertensive status.

| T-HTN- |  |  | MMSE | | | | |  | FAQ | | | | |  | ADAS13 | | | | |  | \ | | | | |
| --- | --- | --- | --- | --- | --- | --- | --- | --- | --- | --- | --- | --- | --- | --- | --- | --- | --- | --- | --- | --- | --- | --- | --- | --- | --- |
|  | Predictors |  | Coefficients |  | SE |  | *P* value |  | Coefficients |  | SE |  | *P* value |  | Coefficients |  | SE |  | *P* value |  | Coefficients |  | SE |  | *P* value |
|  | Age × time |  | 0.006 |  | 0.012 |  | 0.610 |  | -0.093 |  | 0.026 |  | 0.001 |  | -0.093 |  | 0.034 |  | 0.007 |  | \ |  | \ |  | \ |
|  | Female × time |  | -0.138 |  | 0.097 |  | 0.160 |  | 1.371 |  | 0.216 |  | 2.140e-09 |  | 0.622 |  | 0.255 |  | 0.016 |  | \ |  | \ |  | \ |
|  | Education × time |  | -0.043 |  | 0.015 |  | 0.005 |  | 0.273 |  | 0.033 |  | 6.200e-14 |  | 0.013 |  | 0.039 |  | 0.734 |  | \ |  | \ |  | \ |
|  | *APOE ɛ4* × time |  | -0.445 |  | 0.164 |  | 0.008 |  | 0.458 |  | 0.364 |  | 0.209 |  | 0.597 |  | 0.421 |  | 0.159 |  | \ |  | \ |  | \ |
|  | Group high × time |  | 0.133 |  | 0.122 |  | 0.276 |  | -1.419 |  | 0.270 |  | **4.700e-07** |  | 0.637 |  | 0.321 |  | **0.049** |  | \ |  | \ |  | \ |
|  |  |  | MEM | | | | |  | LAN | | | | |  | EF | | | | |  | VP | | | | |
|  | Predictors |  | Coefficients |  | SE |  | *P* value |  | Coefficients |  | SE |  | *P* value |  | Coefficients |  | SE |  | *P* value |  | Coefficients |  | SE |  | *P* value |
|  | Age × time |  | 0.003 |  | 0.001 |  | 0.082 |  | 3.021e-03 |  | 2.223e-03 |  | 0.176 |  | -0.002 |  | 0.002 |  | 0.303 |  | -0.021 |  | 0.005 |  | 4.720e-05 |
|  | Female × time |  | -0.042 |  | 0.012 |  | 0.001 |  | 0.027 |  | 0.019 |  | 0.144 |  | 0.027 |  | 0.020 |  | 0.178 |  | 0.086 |  | 0.046 |  | 0.065 |
|  | Education × time |  | -0.007 |  | 0.002 |  | 0.000 |  | 2.347e-04 |  | 2.939e-03 |  | 0.937 |  | -0.007 |  | 0.003 |  | 0.019 |  | -0.003 |  | 0.006 |  | 0.644 |
|  | *APOE ɛ4* × time |  | -0.063 |  | 0.020 |  | 0.002 |  | -0.017 |  | 0.030 |  | 0.585 |  | -0.045 |  | 0.032 |  | 0.155 |  | -0.114 |  | 0.054 |  | 0.040 |
|  | Group high × time |  | -0.010 |  | 0.015 |  | 0.499 |  | -0.045 |  | 0.023 |  | 0.054 |  | 0.043 |  | 0.024 |  | 0.078 |  | -0.014 |  | 0.058 |  | 0.809 |
|  |  |  | Hippocampus | | | | |  | Entorhinal cortex | | | | |  | Mid temporal lobe | | | | |  | Whole brain | | | | |
|  | Predictors |  | Coefficients |  | SE |  | *P* value |  | Coefficients |  | SE |  | *P* value |  | Coefficients |  | SE |  | *P* value |  | Coefficients |  | SE |  | *P* value |
|  | Age × time |  | 9.828e-04 |  | 1.490e-04 |  | 5.740e-09 |  | 7.775e-04 |  | 1.508e-04 |  | 2.070e-06 |  | 2.713e-04 |  | 1.013e-04 |  | 0.009 |  | 2.431e-04 |  | 5.269e-05 |  | 1.650e-05 |
|  | Female × time |  | -4.560e-03 |  | 1.715e-03 |  | 0.010 |  | -2.185e-03 |  | 1.735e-03 |  | 0.212 |  | 1.423e-05 |  | 1.166e-03 |  | 0.990 |  | -6.651e-04 |  | 6.065e-04 |  | 0.276 |
|  | Education × time |  | -2.394e-03 |  | 3.944e-04 |  | 5.210e-08 |  | -2.326e-03 |  | 3.992e-04 |  | 1.420e-07 |  | -5.791e-04 |  | 2.683e-04 |  | 0.034 |  | -3.315e-04 |  | 1.395e-04 |  | 0.020 |
|  | *APOE ɛ4* × time |  | 5.449e-03 |  | 2.288e-03 |  | 0.020 |  | 1.921e-03 |  | 2.315e-03 |  | 0.409 |  | -3.981e-04 |  | 1.556e-03 |  | 0.799 |  | 2.166e-04 |  | 8.092e-04 |  | 0.790 |
|  | ICV × time |  | 1.504e-08 |  | 5.910e-09 |  | 0.013 |  | 1.687e-08 |  | 5.981e-09 |  | 0.006 |  | 4.915e-09 |  | 4.019e-09 |  | 0.225 |  | 1.634e-09 |  | 2.090e-09 |  | 0.437 |
|  | Group high × time |  | 1.515e-03 |  | 1.752e-03 |  | 0.390 |  | 1.544e-03 |  | 1.774e-03 |  | 0.387 |  | 8.149e-04 |  | 1.192e-03 |  | 0.496 |  | 9.664e-04 |  | 6.198e-04 |  | 0.123 |
| T-HTN+ |  |  | MMSE | | | | |  | FAQ | | | | |  | ADAS13 | | | | |  | \ | | | | |
|  | Predictors |  | Coefficients |  | SE |  | *P* value |  | Coefficients |  | SE |  | *P* value |  | Coefficients |  | SE |  | *P* value |  | Coefficients |  | SE |  | *P* value |
|  | Age × time |  | -0.019 |  | 5.932e-03 |  | 1.960e-03 |  | 0.026 |  | 9.819e-03 |  | 8.511e-03 |  | 0.035 |  | 0.021 |  | 0.100 |  | \ |  | \ |  | \ |
|  | Female × time |  | 0.309 |  | 0.158 |  | 0.052 |  | 0.263 |  | 0.262 |  | 0.316 |  | -0.967 |  | 0.534 |  | 0.072 |  | \ |  | \ |  | \ |
|  | Education × time |  | -0.039 |  | 0.018 |  | 0.032 |  | 0.023 |  | 0.030 |  | 0.431 |  | 0.119 |  | 0.061 |  | 0.051 |  | \ |  | \ |  | \ |
|  | *APOE ɛ4* × time |  | -0.310 |  | 0.126 |  | 0.015 |  | 0.571 |  | 0.208 |  | 6.622e-03 |  | 1.200 |  | 0.363 |  | 1.190e-03 |  | \ |  | \ |  | \ |
|  | Group high × time |  | -0.167 |  | 0.091 |  | 0.068 |  | 0.017 |  | 0.151 |  | 0.909 |  | -0.043 |  | 0.304 |  | 0.886 |  | \ |  | \ |  | \ |
|  |  |  | MEM | | | | |  | LAN | | | | |  | EF | | | | |  | VP | | | | |
|  | Predictors |  | Coefficients |  | SE |  | *P* value |  | Coefficients |  | SE |  | *P* value |  | Coefficients |  | SE |  | *P* value |  | Coefficients |  | SE |  | *P* value |
|  | Age × time |  | -2.605e-03 |  | 1.272e-03 |  | 0.042 |  | 9.853e-04 |  | 1.570e-03 |  | 0.531 |  | -1.609e-03 |  | 1.613e-03 |  | 0.320 |  | 2.441e-03 |  | 2.326e-03 |  | 0.297 |
|  | Female × time |  | 0.037 |  | 0.030 |  | 0.221 |  | 0.048 |  | 0.037 |  | 0.196 |  | 0.021 |  | 0.038 |  | 0.581 |  | -0.139 |  | 0.049 |  | 5.740e-03 |
|  | Education × time |  | 8.653e-04 |  | 3.347e-03 |  | 0.796 |  | -7.901e-03 |  | 4.136e-03 |  | 0.058 |  | -1.211e-03 |  | 4.249e-03 |  | 0.776 |  | 8.922e-03 |  | 5.662e-03 |  | 0.119 |
|  | *APOE ɛ4* × time |  | -0.047 |  | 0.023 |  | 0.047 |  | -0.079 |  | 0.029 |  | 6.580e-03 |  | 2.371e-03 |  | 0.030 |  | 0.936 |  | 0.065 |  | 0.041 |  | 0.119 |
|  | Group high × time |  | -0.017 |  | 0.020 |  | 0.404 |  | -0.067 |  | 0.025 |  | **7.520e-03** |  | 0.036 |  | 0.025 |  | 0.160 |  | 0.068 |  | 0.035 |  | 0.058 |
|  |  |  | Hippocampus | | | | |  | Entorhinal cortex | | | | |  | Mid temporal lobe | | | | |  | Whole brain | | | | |
|  | Predictors |  | Coefficients |  | SE |  | *P* value |  | Coefficients |  | SE |  | *P* value |  | Coefficients |  | SE |  | *P* value |  | Coefficients |  | SE |  | *P* value |
|  | Age × time |  | -7.178e-04 |  | 1.032e-04 |  | 4.450e-09 |  | -2.655e-04 |  | 1.134e-04 |  | 0.023 |  | -1.866e-04 |  | 7.496e-05 |  | 0.016 |  | -1.201e-04 |  | 3.804e-05 |  | 2.595e-03 |
|  | Female × time |  | 7.390e-03 |  | 2.640e-03 |  | 7.060e-03 |  | 3.017e-03 |  | 2.901e-03 |  | 0.303 |  | 5.158e-03 |  | 1.918e-03 |  | 9.470e-03 |  | 2.893e-03 |  | 9.735e-04 |  | 4.387e-03 |
|  | Education × time |  | 5.016e-05 |  | 4.144e-04 |  | 0.904 |  | -2.334e-04 |  | 4.553e-04 |  | 0.610 |  | -7.498e-04 |  | 3.010e-04 |  | 0.016 |  | -3.345e-04 |  | 1.528e-04 |  | 0.033 |
|  | *APOE ɛ4* × time |  | 8.180e-04 |  | 2.639e-03 |  | 0.758 |  | 1.213e-03 |  | 2.900e-03 |  | 0.677 |  | -6.097e-04 |  | 1.917e-03 |  | 0.752 |  | -5.590e-04 |  | 9.732e-04 |  | 0.568 |
|  | ICV × time |  | -2.385e-08 |  | 6.798e-09 |  | 9.070e-04 |  | -4.465e-09 |  | 7.469e-09 |  | 0.552 |  | -4.965e-09 |  | 4.939e-09 |  | 0.319 |  | -4.295e-09 |  | 2.507e-09 |  | 0.092 |
|  | Group high × time |  | -9.569e-03 |  | 1.701e-03 |  | **6.430e-07** |  | -1.548e-03 |  | 1.869e-03 |  | 0.411 |  | -1.759e-03 |  | 1.236e-03 |  | 0.160 |  | -4.761e-04 |  | 6.272e-04 |  | 0.451 |
| T+HTN- |  |  | MMSE | | | | |  | FAQ | | | | |  | ADAS13 | | | | |  | \ | | | | |
|  | Predictors |  | Coefficients |  | SE |  | *P* value |  | Coefficients |  | SE |  | *P* value |  | Coefficients |  | SE |  | *P* value |  | Coefficients |  | SE |  | *P* value |
|  | Age × time |  | 0.067 |  | 0.014 |  | 5.770e-06 |  | -0.066 |  | 0.022 |  | 2.430E-03 |  | -0.087 |  | 0.039 |  | 0.029 |  | \ |  | \ |  | \ |
|  | Female × time |  | -0.024 |  | 0.275 |  | 0.932 |  | -0.478 |  | 0.415 |  | 0.250 |  | -0.433 |  | 0.743 |  | 0.560 |  | \ |  | \ |  | \ |
|  | Education × time |  | 0.096 |  | 0.053 |  | 0.073 |  | 0.031 |  | 0.080 |  | 0.699 |  | -0.048 |  | 0.145 |  | 0.741 |  | \ |  | \ |  | \ |
|  | *APOE ɛ4* × time |  | -0.236 |  | 0.160 |  | 0.140 |  | 1.236 |  | 0.243 |  | 8.290e-07 |  | 1.244 |  | 0.461 |  | 7.500e-03 |  | \ |  | \ |  | \ |
|  | Group high × time |  | -0.398 |  | 0.170 |  | **0.020** |  | 0.112 |  | 0.256 |  | 0.663 |  | -0.629 |  | 0.478 |  | 0.190 |  | \ |  | \ |  | \ |
|  |  |  | MEM | | | | |  | LAN | | | | |  | EF | | | | |  | VP | | | | |
|  | Predictors |  | Coefficients |  | SE |  | *P* value |  | Coefficients |  | SE |  | *P* value |  | Coefficients |  | SE |  | *P* value |  | Coefficients |  | SE |  | *P* value |
|  | Age × time |  | 2.694e-03 |  | 1.429e-03 |  | 0.061 |  | 9.284e-03 |  | 1.681e-03 |  | 1.050e-07 |  | 9.968e-03 |  | 2.002e-03 |  | 1.390e-06 |  | 0.011 |  | 2.756e-03 |  | 1.820e-04 |
|  | Female × time |  | 0.032 |  | 0.028 |  | 0.244 |  | 0.030 |  | 0.033 |  | 0.351 |  | 0.035 |  | 0.038 |  | 0.355 |  | -5.220e-03 |  | 0.051 |  | 0.919 |
|  | Education × time |  | -4.998e-03 |  | 5.470e-03 |  | 0.362 |  | 9.971e-03 |  | 6.425e-03 |  | 0.122 |  | 0.012 |  | 7.415e-03 |  | 0.102 |  | 0.012 |  | 9.814e-03 |  | 0.217 |
|  | *APOE ɛ4* × time |  | -0.067 |  | 0.017 |  | 1.580e-04 |  | -5.976e-03 |  | 0.020 |  | 0.769 |  | -0.030 |  | 0.023 |  | 0.206 |  | 9.431e-03 |  | 0.034 |  | 0.781 |
|  | Group high × time |  | -0.025 |  | 0.017 |  | 0.144 |  | -0.036 |  | 0.020 |  | 0.074 |  | -0.025 |  | 0.024 |  | 0.305 |  | -0.099 |  | 0.037 |  | **8.637e-03** |
|  |  |  | Hippocampus | | | | |  | Entorhinal cortex | | | | |  | Mid temporal lobe | | | | |  | Whole brain | | | | |
|  | Predictors |  | Coefficients |  | SE |  | *P* value |  | Coefficients |  | SE |  | *P* value |  | Coefficients |  | SE |  | *P* value |  | Coefficients |  | SE |  | *P* value |
|  | Age × time |  | 1.683e-04 |  | 1.334e-04 |  | 0.210 |  | 1.178e-04 |  | 1.514e-04 |  | 0.438 |  | 2.985e-04 |  | 1.507e-04 |  | 0.050 |  | 1.717e-04 |  | 6.379-05 |  | 8.260e-03 |
|  | Female × time |  | -1.743e-03 |  | 1.825e-03 |  | 0.342 |  | -1.444e-03 |  | 2.073e-03 |  | 0.488 |  | 2.322e-03 |  | 2.063e-03 |  | 0.263 |  | 1.221e-03 |  | 8.731e-04 |  | 0.165 |
|  | Education × time |  | 1.480e-03 |  | 3.635e-04 |  | 9.070e-05 |  | 1.776e-03 |  | 4.129e-04 |  | 3.770e-05 |  | 9.760e-04 |  | 4.108e-04 |  | 0.019 |  | 4.667e-04 |  | 1.739e-04 |  | 8.440e-03 |
|  | *APOE ɛ4* × time |  | -7.580e-03 |  | 1.776e-03 |  | 4.300e-05 |  | -7.226e-03 |  | 2.017e-03 |  | 5.150e-04 |  | -4.328e-03 |  | 2.007e-03 |  | 0.033 |  | -8.119e-04 |  | 8.494-04 |  | 0.341 |
|  | ICV × time |  | 1.006e-08 |  | 6.435e-09 |  | 0.121 |  | 5.568e-09 |  | 7.308e-09 |  | 0.448 |  | -4.177e-09 |  | 7.273e-09 |  | 0.567 |  | -5.542e-09 |  | 3.078e-09 |  | 0.075 |
|  | Group high × time |  | -2.083e-03 |  | 1.563e-03 |  | 0.185 |  | -1.246e-04 |  | 1.775e-03 |  | 0.944 |  | -1.541e-03 |  | 1.766e-03 |  | 0.385 |  | -1.180e-03 |  | 7.475e-04 |  | 0.118 |
| T+HTN+ |  |  | MMSE | | | | |  | FAQ | | | | |  | ADAS13 | | | | |  | \ | | | | |
|  | Predictors |  | Coefficients |  | SE |  | *P* value |  | Coefficients |  | SE |  | *P* value |  | Coefficients |  | SE |  | *P* value |  | Coefficients |  | SE |  | *P* value |
|  | Age × time |  | -0.036 |  | 0.012 |  | 4.370e-03 |  | 0.093 |  | 0.017 |  | 9.280E-08 |  | -0.018 |  | 0.032 |  | 0.577 |  | \ |  | \ |  | \ |
|  | Female × time |  | 0.055 |  | 0.141 |  | 0.698 |  | 0.202 |  | 0.188 |  | 0.283 |  | -1.127 |  | 0.387 |  | 3.970e-03 |  | \ |  | \ |  | \ |
|  | Education × time |  | 0.047 |  | 0.022 |  | 0.037 |  | -0.205 |  | 0.030 |  | 3.190e-11 |  | -0.029 |  | 0.061 |  | 0.636 |  | \ |  | \ |  | \ |
|  | *APOE ɛ4* × time |  | -1.192 |  | 0.160 |  | 1.460e-12 |  | 2.634 |  | 0.213 |  | < 2e-16 |  | 2.230 |  | 0.402 |  | 7.450e-08 |  | \ |  | \ |  | \ |
|  | Group high × time |  | 0.102 |  | 0.139 |  | 0.467 |  | -0.040 |  | 0.188 |  | 0.830 |  | -0.052 |  | 0.359 |  | 0.885 |  | \ |  | \ |  | \ |
|  |  |  | MEM | | | | |  | LAN | | | | |  | EF | | | | |  | VP | | | | |
|  | Predictors |  | Coefficients |  | SE |  | *P* value |  | Coefficients |  | SE |  | *P* value |  | Coefficients |  | SE |  | *P* value |  | Coefficients |  | SE |  | *P* value |
|  | Age × time |  | -2.269e-03 |  | 1.345e-03 |  | 0.093 |  | -2.469e-03 |  | 1.591e-03 |  | 0.122 |  | 1.243e-03 |  | 1.709e-03 |  | 0.468 |  | 8.775e-03 |  | 3.147e-03 |  | 5.950e-03 |
|  | Female × time |  | 0.049 |  | 0.017 |  | 3.680e-03 |  | 4.482e-03 |  | 0.020 |  | 0.821 |  | 4.352e-02 |  | 0.021 |  | 0.042 |  | -0.021 |  | 0.046 |  | 0.643 |
|  | Education × time |  | 7.857e-03 |  | 2.484e-03 |  | 1.770e-03 |  | -1.203e-03 |  | 2.942e-03 |  | 0.683 |  | -6.944e-03 |  | 3.151e-03 |  | 0.029 |  | -2.509e-03 |  | 7.018e-03 |  | 0.721 |
|  | *APOE ɛ4* × time |  | -0.128 |  | 0.017 |  | 1.060e-12 |  | -0.112 |  | 0.020 |  | 5.810e-08 |  | -0.148 |  | 0.022 |  | 6.810e-11 |  | -0.034 |  | 0.043 |  | 0.429 |
|  | Group high × time |  | 2.709e-03 |  | 0.015 |  | 0.860 |  | 0.053 |  | 0.018 |  | **3.620e-03** |  | 0.049 |  | 0.020 |  | **0.013** |  | -3.912e-03 |  | 0.043 |  | 0.927 |
|  |  |  | Hippocampus | | | | |  | Entorhinal cortex | | | | |  | Mid temporal lobe | | | | |  | Whole brain | | | | |
|  | Predictors |  | Coefficients |  | SE |  | *P* value |  | Coefficients |  | SE |  | *P* value |  | Coefficients |  | SE |  | *P* value |  | Coefficients |  | SE |  | *P* value |
|  | Age × time |  | -3.285e-04 |  | 1.167e-04 |  | 5.670e-03 |  | 8.306-05 |  | 1.204e-04 |  | 0.492 |  | 2.528e-04 |  | 1.054e-04 |  | 0.018 |  | 1.131e-04 |  | 3.952e-05 |  | 4.923e-03 |
|  | Female × time |  | 2.328e-03 |  | 1.965e-03 |  | 0.238 |  | 4.253e-03 |  | 2.027e-03 |  | 0.038 |  | 7.611-04 |  | 1.775e-03 |  | 0.669 |  | 7.498e-04 |  | 6.652e-04 |  | 0.262 |
|  | Education × time |  | 2.847e-04 |  | 2.744e-04 |  | 0.301 |  | -1.303e-04 |  | 2.831-04 |  | 0.646 |  | -2.306e-04 |  | 2.478e-04 |  | 0.354 |  | 1.459e-05 |  | 9.290e-05 |  | 0.875 |
|  | *APOE ɛ4* × time |  | -8.035e-03 |  | 1.622e-03 |  | 2.260e-06 |  | -7.253e-03 |  | 1.674e-03 |  | 2.930e-05 |  | -2.766e-03 |  | 1.465e-03 |  | 0.061 |  | -4.170e-04 |  | 5.493e-04 |  | 0.449 |
|  | ICV × time |  | -1.667e-08 |  | 7.376e-09 |  | 0.026 |  | 6.812e-09 |  | 7.610e-09 |  | 0.372 |  | 6.556e-09 |  | 6.663e-09 |  | 0.327 |  | 9.151e-10 |  | 2.498e-09 |  | 0.715 |
|  | Group high × time |  | -6.618e-04 |  | 1.636e-03 |  | 0.687 |  | -5.827e-03 |  | 1.688e-03 |  | **7.550e-04** |  | -4.435e-03 |  | 1.478e-03 |  | **3.240e-03** |  | -1.909e-03 |  | 5.541e-04 |  | **7.730e-04** |

The primary effects of the predictive factors (i.e., age, sex, years of education, *APOE ɛ4* status, years since baseline, and ICV when appropriate) were incorporated into all linear mixed-effects models, while coefficients are not shown for the sake of brevity.

Abbreviations: CSF, cerebrospinal fluid; T-HTN-: T negative and normotension; T-HTN+: T positive and hypertension; T+HTN-: T positive and normotension; T+HTN+: T positive and hypertension; MMSE, Mini-Mental State Examination; FAQ, Functional Assessment Questionnaire; ADAS13, Alzheimer’s disease Assessment Scale 13; MEM, memory function; LAN, language; EF, executive function; VP, visuospatial functioning; *APOE ε4*, apolipoprotein *E4*; ICV, Intracranial volume; SE, Standard Error; Group high, CSF YKL-40 ≥ 390ng/ml (determined by the median concentration).

**Table S16** Baseline CSF YKL-40 and longitudinal changes in cognition and MRI brain structures stratified by A status.

| A- |  |  | MMSE | | | | |  | FAQ | | | | |  | ADAS13 | | | | |  | \ | | | | |
| --- | --- | --- | --- | --- | --- | --- | --- | --- | --- | --- | --- | --- | --- | --- | --- | --- | --- | --- | --- | --- | --- | --- | --- | --- | --- |
|  | Predictors |  | Coefficients |  | SE |  | *P* value |  | Coefficients |  | SE |  | *P* value |  | Coefficients |  | SE |  | *P* value |  | Coefficients |  | SE |  | *P* value |
|  | Age × time |  | -0.015 |  | 3.781e-03 |  | 8.050e-05 |  | 0.038 |  | 7.853e-03 |  | 2.350e-06 |  | 0.032 |  | 0.012 |  | 7.760e-03 |  | \ |  | \ |  | \ |
|  | Female × time |  | -0.014 |  | 0.056 |  | 0.809 |  | 0.428 |  | 0.117 |  | 2.860e-04 |  | -0.159 |  | 0.172 |  | 0.355 |  | \ |  | \ |  | \ |
|  | Education × time |  | -0.024 |  | 8.070e-03 |  | 2.860e-03 |  | 0.023 |  | 0.017 |  | 0.170 |  | 0.036 |  | 0.024 |  | 0.138 |  | \ |  | \ |  | \ |
|  | *APOE ɛ4* × time |  | 0.114 |  | 0.084 |  | 0.176 |  | -0.303 |  | 0.171 |  | 0.077 |  | -0.164 |  | 0.262 |  | 0.532 |  | \ |  | \ |  | \ |
|  | Group high × time |  | 0.063 |  | 0.067 |  | 0.350 |  | -0.075 |  | 0.139 |  | 0.590 |  | -0.211 |  | 0.211 |  | 0.318 |  | \ |  | \ |  | \ |
|  |  |  | MEM | | | | |  | LAN | | | | |  | EF | | | | |  | VP | | | | |
|  | Predictors |  | Coefficients |  | SE |  | *P* value |  | Coefficients |  | SE |  | *P* value |  | Coefficients |  | SE |  | *P* value |  | Coefficients |  | SE |  | *P* value |
|  | Age × time |  | -2.585e-03 |  | 7.556e-04 |  | 6.980e-04 |  | 3.419e-04 |  | 8.746e-04 |  | 0.696 |  | -2.460e-03 |  | 8.451e-04 |  | 3.840e-03 |  | -1.988e-03 |  | 1.588e-03 |  | 0.212 |
|  | Female × time |  | -2.357e-03 |  | 0.012 |  | 0.844 |  | 1.806e-03 |  | 0.014 |  | 0.896 |  | 0.020 |  | 0.013 |  | 0.133 |  | -0.016 |  | 0.024 |  | 0.496 |
|  | Education × time |  | -3.947e-03 |  | 1.610e-03 |  | 0.015 |  | -3.157e-03 |  | 1.865e-03 |  | 0.091 |  | -3.539e-03 |  | 1.805e-03 |  | 0.051 |  | -3.481e-03 |  | 3.378e-03 |  | 0.304 |
|  | *APOE ɛ4* × time |  | 0.024 |  | 0.016 |  | 0.126 |  | 0.012 |  | 0.018 |  | 0.502 |  | 0.028 |  | 0.018 |  | 0.111 |  | -0.015 |  | 0.028 |  | 0.601 |
|  | Group high × time |  | -4.893e-03 |  | 0.014 |  | 0.731 |  | -5.059e-03 |  | 0.016 |  | 0.759 |  | 0.043 |  | 0.016 |  | **7.350e-03** |  | -8.629e-03 |  | 0.033 |  | 0.793 |
|  |  |  | Hippocampus | | | | |  | Entorhinal cortex | | | | |  | Mid temporal lobe | | | | |  | Whole brain | | | | |
|  | Predictors |  | Coefficients |  | SE |  | *P* value |  | Coefficients |  | SE |  | *P* value |  | Coefficients |  | SE |  | *P* value |  | Coefficients |  | SE |  | *P* value |
|  | Age × time |  | -1.369e-04 |  | 1.010e-04 |  | 0.177 |  | -9.843e-05 |  | 9.511e-05 |  | 0.302 |  | -1.482e-05 |  | 5.541e-05 |  | 0.790 |  | 2.220e-06 |  | 3.121e-05 |  | 0.943 |
|  | Female × time |  | 9.217e-04 |  | 1.667e-03 |  | 0.581 |  | 4.237e-03 |  | 1.571e-03 |  | 7.800e-03 |  | 6.252e-04 |  | 9.150e-04 |  | 0.496 |  | 2.974-04 |  | 5.153e-04 |  | 0.565 |
|  | Education × time |  | -2.275e-04 |  | 2.706e-04 |  | 0.402 |  | -5.276e-04 |  | 2.549e-04 |  | 0.040 |  | -2.644e-04 |  | 1.485e-04 |  | 0.077 |  | -9.838e-05 |  | 8.364e-05 |  | 0.241 |
|  | *APOE ɛ4* × time |  | 2.989e-04 |  | 2.682e-03 |  | 0.911 |  | -2.767e-03 |  | 2.526-03 |  | 0.275 |  | 1.378e-03 |  | 1.472e-03 |  | 0.351 |  | 7.774e-04 |  | 8.288e-04 |  | 0.350 |
|  | ICV × time |  | -2.244e-09 |  | 5.232e-09 |  | 0.669 |  | -4.755e-11 |  | 4.929e-09 |  | 0.992 |  | 1.163e-09 |  | 2.871e-09 |  | 0.686 |  | -1.987e-09 |  | 1.617e-09 |  | 0.221 |
|  | Group high × time |  | -5.165e-03 |  | 1.427e-03 |  | **0.000** |  | -3.062e-03 |  | 1.344e-03 |  | **0.024** |  | -1.495e-03 |  | 7.829e-04 |  | 0.058 |  | -4.742e-05 |  | 4.409e-04 |  | 0.915 |
| A+ |  |  | MMSE | | | | |  | FAQ | | | | |  | ADAS13 | | | | |  | \ | | | | |
|  | Predictors |  | Coefficients |  | SE |  | *P* value |  | Coefficients |  | SE |  | *P* value |  | Coefficients |  | SE |  | *P* value |  | Coefficients |  | SE |  | *P* value |
|  | Age × time |  | 0.030 |  | 9.964e-03 |  | 3.020e-03 |  | -0.039 |  | 0.015 |  | 0.010 |  | -0.091 |  | 0.026 |  | 4.390e-04 |  | \ |  | \ |  | \ |
|  | Female × time |  | 0.459 |  | 0.145 |  | 1.640e-03 |  | -0.437 |  | 0.219 |  | 0.047 |  | -1.026 |  | 0.365 |  | 5.174e-03 |  | \ |  | \ |  | \ |
|  | Education × time |  | 0.024 |  | 0.023 |  | 0.291 |  | -0.121 |  | 0.035 |  | 0.000 |  | 1.328e-03 |  | 0.060 |  | 0.982 |  | \ |  | \ |  | \ |
|  | *APOE ɛ4* × time |  | -0.263 |  | 0.115 |  | 0.023 |  | 0.937 |  | 0.175 |  | 1.520e-07 |  | 1.254 |  | 0.308 |  | 5.570e-05 |  | \ |  | \ |  | \ |
|  | Group high × time |  | -0.262 |  | 0.132 |  | **0.048** |  | -0.069 |  | 0.200 |  | 0.730 |  | -0.259 |  | 0.334 |  | 0.439 |  | \ |  | \ |  | \ |
|  |  |  | MEM | | | | |  | LAN | | | | |  | EF | | | | |  | VP | | | | |
|  | Predictors |  | Coefficients |  | SE |  | *P* value |  | Coefficients |  | SE |  | *P* value |  | Coefficients |  | SE |  | *P* value |  | Coefficients |  | SE |  | *P* value |
|  | Age × time |  | 2.579e-03 |  | 9.977e-04 |  | 0.010 |  | 4.027e-03 |  | 1.205e-03 |  | 9.120e-04 |  | 7.288e-03 |  | 1.350e-03 |  | 1.150e-07 |  | 7.116e-03 |  | 1.957e-03 |  | 3.250e-04 |
|  | Female × time |  | 0.057 |  | 0.014 |  | 6.380e-05 |  | 0.094 |  | 0.017 |  | 5.640e-08 |  | 0.104 |  | 0.019 |  | 7.790e-08 |  | 0.058 |  | 0.031 |  | 0.063 |
|  | Education × time |  | 7.096e-03 |  | 2.328e-03 |  | 2.450e-03 |  | -3.140e-03 |  | 2.812e-03 |  | 0.265 |  | -0.010 |  | 3.123e-03 |  | 9.530e-04 |  | -4.817e-03 |  | 5.183e-03 |  | 0.353 |
|  | *APOE ɛ4* × time |  | -0.056 |  | 0.012 |  | 4.540e-06 |  | -0.061 |  | 0.015 |  | 3.010e-05 |  | -0.060 |  | 0.016 |  | 2.150e-04 |  | -5.714e-03 |  | 0.025 |  | 0.821 |
|  | Group high × time |  | -1.316e-03 |  | 0.013 |  | 0.919 |  | 0.013 |  | 0.016 |  | 0.406 |  | -0.017 |  | 0.017 |  | 0.345 |  | -0.095 |  | 0.026 |  | **3.450e-04** |
|  |  |  | Hippocampus | | | | |  | Entorhinal cortex | | | | |  | Mid temporal lobe | | | | |  | Whole brain | | | | |
|  | Predictors |  | Coefficients |  | SE |  | *P* value |  | Coefficients |  | SE |  | *P* value |  | Coefficients |  | SE |  | *P* value |  | Coefficients |  | SE |  | *P* value |
|  | Age × time |  | -1.287e-04 |  | 1.058e-04 |  | 0.225 |  | 1.497-04 |  | 1.115e-04 |  | 0.181 |  | 2.597-04 |  | 9.851e-05 |  | 8.970e-03 |  | 1.265e-04 |  | 3.922e-05 |  | 1.450e-03 |
|  | Female × time |  | 2.411e-03 |  | 1.419e-03 |  | 0.091 |  | 2.675e-03 |  | 1.495e-03 |  | 0.075 |  | 2.722e-03 |  | 1.322e-03 |  | 0.041 |  | 1.254e-03 |  | 5.261e-04 |  | 0.018 |
|  | Education × time |  | 5.995e-04 |  | 2.324e-04 |  | 0.011 |  | 7.211e-05 |  | 2.448e-04 |  | 0.769 |  | 1.010e-04 |  | 2.163e-04 |  | 0.641 |  | 1.353e-04 |  | 8.612e-05 |  | 0.118 |
|  | *APOE ɛ4* × time |  | -3.874e-03 |  | 1.322e-03 |  | 3.750e-03 |  | -4.972e-03 |  | 1.393e-03 |  | 4.390e-04 |  | -2.790e-03 |  | 1.231e-03 |  | 0.024 |  | -7.682e-04 |  | 4.901e-04 |  | 0.118 |
|  | ICV × time |  | -2.558e-09 |  | 4.984e-09 |  | 0.608 |  | 7.160e-09 |  | 5.250e-09 |  | 0.174 |  | 8.793e-10 |  | 4.640e-09 |  | 0.850 |  | -2.066e-09 |  | 1.847e-09 |  | 0.264 |
|  | Group high × time |  | 8.650e-05 |  | 1.183e-03 |  | 0.942 |  | -1.765e-03 |  | 1.246e-03 |  | 0.158 |  | -1.452e-03 |  | 1.102e-03 |  | 0.189 |  | -6.616e-04 |  | 4.386e-04 |  | 0.133 |

The primary effects of the predictive factors (i.e., age, sex, years of education, *APOE ɛ4* status, years since baseline, and ICV when appropriate) were incorporated into all linear mixed-effects models, while coefficients are not shown for the sake of brevity.

Abbreviations: CSF, cerebrospinal fluid; A-: A negative; A+: A positive; MMSE, Mini-Mental State Examination; FAQ, Functional Assessment Questionnaire; ADAS13, Alzheimer’s disease Assessment Scale 13; MEM, memory function; LAN, language; EF, executive function; VP, visuospatial functioning; *APOE ε4*, apolipoprotein *E4*; ICV, Intracranial volume; SE, Standard Error; Group high, CSF YKL-40 ≥ 390ng/ml (determined by the median concentration).

**Table S17** Baseline CSF YKL-40 and longitudinal changes in cognition and MRI brain structures stratified by A status and hypertensive status.

| A-HTN- |  |  | MMSE | | | | |  | FAQ | | | | |  | ADAS13 | | | | |  | \ | | | | |
| --- | --- | --- | --- | --- | --- | --- | --- | --- | --- | --- | --- | --- | --- | --- | --- | --- | --- | --- | --- | --- | --- | --- | --- | --- | --- |
|  | Predictors |  | Coefficients |  | SE |  | *P* value |  | Coefficients |  | SE |  | *P* value |  | Coefficients |  | SE |  | *P* value |  | Coefficients |  | SE |  | *P* value |
|  | Age × time |  | 4.515e-04 |  | 9.796e-03 |  | 0.963 |  | -0.023 |  | 0.020 |  | 0.266 |  | 0.026 |  | 0.027 |  | 0.351 |  | \ |  | \ |  | \ |
|  | Female × time |  | -0.166 |  | 0.140 |  | 0.237 |  | 1.456 |  | 0.292 |  | 1.620e-06 |  | 0.590 |  | 0.359 |  | 0.102 |  | \ |  | \ |  | \ |
|  | Education × time |  | -0.056 |  | 0.024 |  | 0.023 |  | 0.292 |  | 0.050 |  | 3.640e-08 |  | 0.110 |  | 0.061 |  | 0.076 |  | \ |  | \ |  | \ |
|  | *APOE ɛ4* × time |  | 0.320 |  | 0.635 |  | 0.615 |  | -2.564 |  | 1.327 |  | 0.055 |  | -1.229 |  | 1.561 |  | 0.432 |  | \ |  | \ |  | \ |
|  | Group high × time |  | 0.269 |  | 0.229 |  | 0.242 |  | -1.950 |  | 0.476 |  | **6.740e-05** |  | -0.718 |  | 0.595 |  | 0.229 |  | \ |  | \ |  | \ |
|  |  |  | MEM | | | | |  | LAN | | | | |  | EF | | | | |  | VP | | | | |
|  | Predictors |  | Coefficients |  | SE |  | *P* value |  | Coefficients |  | SE |  | *P* value |  | Coefficients |  | SE |  | *P* value |  | Coefficients |  | SE |  | *P* value |
|  | Age × time |  | 6.080e-04 |  | 1.356e-03 |  | 0.655 |  | 3.980e-03 |  | 1.834e-03 |  | 0.032 |  | -2.023e-03 |  | 1.882e-03 |  | 0.284 |  | -8.697e-03 |  | 3.206e-03 |  | 8.220e-03 |
|  | Female × time |  | -0.059 |  | 0.020 |  | 3.750e-03 |  | 0.027 |  | 0.027 |  | 0.328 |  | -8.175e-03 |  | 0.028 |  | 0.769 |  | 0.028 |  | 0.053 |  | 0.595 |
|  | Education × time |  | -0.015 |  | 3.366e-03 |  | 2.320e-05 |  | 4.670e-04 |  | 4.569e-03 |  | 0.919 |  | -7.492e-03 |  | 4.688e-03 |  | 0.112 |  | 5.999e-03 |  | 9.861e-03 |  | 0.545 |
|  | *APOE ɛ4* × time |  | 0.051 |  | 0.085 |  | 0.545 |  | -0.066 |  | 0.116 |  | 0.567 |  | 0.039 |  | 0.119 |  | 0.745 |  | -0.114 |  | 0.054 |  | 0.395 |
|  | Group high × time |  | 0.061 |  | 0.032 |  | 0.063 |  | -0.054 |  | 0.044 |  | 0.223 |  | 0.059 |  | 0.045 |  | 0.193 |  | -0.119 |  | 0.097 |  | 0.225 |
|  |  |  | Hippocampus | | | | |  | Entorhinal cortex | | | | |  | Mid temporal lobe | | | | |  | Whole brain | | | | |
|  | Predictors |  | Coefficients |  | SE |  | *P* value |  | Coefficients |  | SE |  | *P* value |  | Coefficients |  | SE |  | *P* value |  | Coefficients |  | SE |  | *P* value |
|  | Age × time |  | 4.531e-04 |  | 1.613e-04 |  | 6.540e-03 |  | 1.042e-06 |  | 1.706e-04 |  | 0.995 |  | 5.778e-05 |  | 8.321e-05 |  | 0.490 |  | 1.475e-04 |  | 5.433e-05 |  | 8.440e-03 |
|  | Female × time |  | -6.306e-03 |  | 2.625e-03 |  | 0.019 |  | -3.110e-04 |  | 2.775e-03 |  | 0.911 |  | -3.210e-03 |  | 1.354e-03 |  | 0.021 |  | -2.074e-03 |  | 8.839e-04 |  | 0.022 |
|  | Education × time |  | -8.371e-04 |  | 4.426e-04 |  | 0.063 |  | -3.359e-04 |  | 4.680e-04 |  | 0.475 |  | -9.781e-06 |  | 2.283e-04 |  | 0.966 |  | -9.744e-05 |  | 1.490e-04 |  | 0.516 |
|  | *APOE ɛ4* × time |  | 9.559e-03 |  | 5.000e-03 |  | 0.060 |  | 1.896e-03 |  | 5.287e-03 |  | 0.721 |  | 1.856e-03 |  | 2.579e-03 |  | 0.474 |  | 1.187e-03 |  | 1.684e-03 |  | 0.483 |
|  | ICV × time |  | -1.162e-08 |  | 7.168e-09 |  | 0.110 |  | -6.497e-09 |  | 7.578e-09 |  | 0.394 |  | -4.103e-10 |  | 3.697e-09 |  | 0.912 |  | -4.622e-09 |  | 2.414e-09 |  | 0.060 |
|  | Group high × time |  | 4.560e-03 |  | 2.751e-03 |  | 0.102 |  | -7.722e-04 |  | 2.909e-03 |  | 0.791 |  | 9.726e-05 |  | 1.419e-03 |  | 0.946 |  | 2.037e-03 |  | 9.265e-04 |  | **0.031** |
| A-HTN+ |  |  | MMSE | | | | |  | FAQ | | | | |  | ADAS13 | | | | |  | \ | | | | |
|  | Predictors |  | Coefficients |  | SE |  | *P* value |  | Coefficients |  | SE |  | *P* value |  | Coefficients |  | SE |  | *P* value |  | Coefficients |  | SE |  | *P* value |
|  | Age × time |  | 4.515e-04 |  | 9.796e-03 |  | 0.963 |  | -0.023 |  | 0.020 |  | 0.266 |  | 0.026 |  | 0.027 |  | 0.351 |  | \ |  | \ |  | \ |
|  | Female × time |  | -0.166 |  | 0.140 |  | 0.237 |  | 1.456 |  | 0.292 |  | 1.620e-06 |  | 0.590 |  | 0.359 |  | 0.102 |  | \ |  | \ |  | \ |
|  | Education × time |  | -0.056 |  | 0.024 |  | 0.023 |  | 0.292 |  | 0.050 |  | 3.640e-08 |  | 0.110 |  | 0.061 |  | 0.076 |  | \ |  | \ |  | \ |
|  | *APOE ɛ4* × time |  | 0.320 |  | 0.635 |  | 0.615 |  | -2.564 |  | 1.327 |  | 0.055 |  | -1.229 |  | 1.561 |  | 0.432 |  | \ |  | \ |  | \ |
|  | Group high × time |  | 0.269 |  | 0.229 |  | 0.242 |  | -1.950 |  | 0.476 |  | **6.740e-05** |  | -0.718 |  | 0.595 |  | 0.229 |  | \ |  | \ |  | \ |
|  |  |  | MEM | | | | |  | LAN | | | | |  | EF | | | | |  | VP | | | | |
|  | Predictors |  | Coefficients |  | SE |  | *P* value |  | Coefficients |  | SE |  | *P* value |  | Coefficients |  | SE |  | *P* value |  | Coefficients |  | SE |  | *P* value |
|  | Age × time |  | 6.080e-04 |  | 1.356e-03 |  | 0.655 |  | 3.980e-03 |  | 1.834e-03 |  | 0.032 |  | -2.023e-03 |  | 1.882e-03 |  | 0.284 |  | -8.697e-03 |  | 3.206e-03 |  | 8.220e-03 |
|  | Female × time |  | -0.059 |  | 0.020 |  | 3.750e-03 |  | 0.027 |  | 0.027 |  | 0.328 |  | -8.175e-03 |  | 0.028 |  | 0.769 |  | 0.028 |  | 0.053 |  | 0.595 |
|  | Education × time |  | -0.015 |  | 3.366e-03 |  | 2.320e-05 |  | 4.670e-04 |  | 4.569e-03 |  | 0.919 |  | -7.492e-03 |  | 4.688e-03 |  | 0.112 |  | 5.999e-03 |  | 9.861E-03 |  | 0.545 |
|  | *APOE ɛ4* × time |  | 0.051 |  | 0.085 |  | 0.545 |  | -0.066 |  | 0.116 |  | 0.567 |  | 0.039 |  | 0.119 |  | 0.745 |  | \ |  | \ |  | \ |
|  | Group high × time |  | 0.061 |  | 0.032 |  | 0.063 |  | -0.054 |  | 0.044 |  | 0.223 |  | 0.059 |  | 0.045 |  | 0.193 |  | -0.119 |  | 0.097 |  | 0.225 |
|  |  |  | Hippocampus | | | | |  | Entorhinal cortex | | | | |  | Mid temporal lobe | | | | |  | Whole brain | | | | |
|  | Predictors |  | Coefficients |  | SE |  | *P* value |  | Coefficients |  | SE |  | *P* value |  | Coefficients |  | SE |  | *P* value |  | Coefficients |  | SE |  | *P* value |
|  | Age × time |  | 4.531e-04 |  | 1.613e-04 |  | 6.540e-03 |  | 1.042e-06 |  | 1.706e-04 |  | 0.995 |  | 5.778e-05 |  | 8.321e-05 |  | 0.490 |  | 1.475e-04 |  | 5.433e-05 |  | 8.440e-03 |
|  | Female × time |  | -6.306e-03 |  | 2.625e-03 |  | 0.019 |  | -3.110e-04 |  | 2.775e-03 |  | 0.911 |  | -3.210e-03 |  | 1.354e-03 |  | 0.021 |  | -2.074e-03 |  | 8.839e-04 |  | 0.022 |
|  | Education × time |  | -8.371e-04 |  | 4.426e-04 |  | 0.063 |  | -3.359e-04 |  | 4.680e-04 |  | 0.475 |  | -9.781e-06 |  | 2.283e-04 |  | 0.966 |  | -9.744e-05 |  | 1.490e-04 |  | 0.516 |
|  | *APOE ɛ4* × time |  | 9.559e-03 |  | 5.000e-03 |  | 0.060 |  | 1.896e-03 |  | 5.287e-03 |  | 0.721 |  | 1.856e-03 |  | 2.579e-03 |  | 0.474 |  | 1.187e-03 |  | 1.684e-03 |  | 0.483 |
|  | ICV × time |  | -1.162e-08 |  | 7.168e-09 |  | 0.110 |  | -6.497e-09 |  | 7.578e-09 |  | 0.394 |  | -4.103e-10 |  | 3.697e-09 |  | 0.912 |  | -4.622e-09 |  | 2.414e-09 |  | 0.060 |
|  | Group high × time |  | 4.560e-03 |  | 2.751e-03 |  | 0.102 |  | -7.722e-04 |  | 2.909e-03 |  | 0.791 |  | 9.726e-05 |  | 1.419e-03 |  | 0.946 |  | 2.037e-03 |  | 9.265e-04 |  | **0.031** |
| A+HTN- |  |  | MMSE | | | | |  | FAQ | | | | |  | ADAS13 | | | | |  | \ | | | | |
|  | Predictors |  | Coefficients |  | SE |  | *P* value |  | Coefficients |  | SE |  | *P* value |  | Coefficients |  | SE |  | *P* value |  | Coefficients |  | SE |  | *P* value |
|  | Age × time |  | 0.060 |  | 0.014 |  | 5.000e-05 |  | -0.041 |  | 0.023 |  | 0.080 |  | -0.100 |  | 0.040 |  | 0.013 |  | \ |  | \ |  | \ |
|  | Female × time |  | 0.156 |  | 0.200 |  | 0.437 |  | -0.461 |  | 0.319 |  | 0.150 |  | -0.702 |  | 0.531 |  | 0.187 |  | \ |  | \ |  | \ |
|  | Education × time |  | 0.068 |  | 0.040 |  | 0.091 |  | -0.087 |  | 0.064 |  | 0.177 |  | -0.037 |  | 0.113 |  | 0.741 |  | \ |  | \ |  | \ |
|  | *APOE ɛ4* × time |  | -0.151 |  | 0.160 |  | 0.347 |  | 0.975 |  | 0.258 |  | 2.040e-04 |  | 1.131 |  | 0.470 |  | 0.017 |  | \ |  | \ |  | \ |
|  | Group high × time |  | -0.353 |  | 0.205 |  | 0.086 |  | -0.941 |  | 0.325 |  | **0.004** |  | -0.713 |  | 0.566 |  | 0.210 |  | \ |  | \ |  | \ |
|  |  |  | MEM | | | | |  | LAN | | | | |  | EF | | | | |  | VP | | | | |
|  | Predictors |  | Coefficients |  | SE |  | *P* value |  | Coefficients |  | SE |  | *P* value |  | Coefficients |  | SE |  | *P* value |  | Coefficients |  | SE |  | *P* value |
|  | Age × time |  | 1.779e-03 |  | 1.389e-03 |  | 0.202 |  | 7.825e-03 |  | 1.704-03 |  | 7.490e-06 |  | 9.146e-03 |  | 2.036e-03 |  | 1.160e-05 |  | 9.497e-03 |  | 3.174e-03 |  | 3.210e-03 |
|  | Female × time |  | 0.026 |  | 0.019 |  | 0.180 |  | 0.095 |  | 0.023 |  | 6.900e-05 |  | 0.095 |  | 0.027 |  | 6.060e-04 |  | 0.056 |  | 0.040 |  | 0.162 |
|  | Education × time |  | 2.049e-03 |  | 4.070e-03 |  | 0.615 |  | 3.756e-03 |  | 4.983e-03 |  | 0.452 |  | 8.099e-04 |  | 5.756e-03 |  | 0.888 |  | 1.788e-03 |  | 8.020e-03 |  | 0.824 |
|  | *APOE ɛ4* × time |  | -0.067 |  | 0.017 |  | 9.710e-05 |  | -3.211e-03 |  | 0.021 |  | 0.877 |  | -0.013 |  | 0.024 |  | 0.591 |  | 0.034 |  | 0.037 |  | 0.363 |
|  | Group high × time |  | 0.024 |  | 0.019 |  | 0.221 |  | 6.362e-03 |  | 0.024 |  | 0.790 |  | -0.026 |  | 0.029 |  | 0.376 |  | -0.133 |  | 0.043 |  | **2.340e-03** |
|  |  |  | Hippocampus | | | | |  | Entorhinal cortex | | | | |  | Mid temporal lobe | | | | |  | Whole brain | | | | |
|  | Predictors |  | Coefficients |  | SE |  | *P* value |  | Coefficients |  | SE |  | *P* value |  | Coefficients |  | SE |  | *P* value |  | Coefficients |  | SE |  | *P* value |
|  | Age × time |  | -1.243e-04 |  | 1.568e-04 |  | 0.430 |  | -5.706e-06 |  | 1.805e-04 |  | 0.975 |  | 1.062e-04 |  | 1.574e-04 |  | 0.501 |  | 8.191e-05 |  | 6.525e-05 |  | 0.212 |
|  | Female × time |  | 4.487e-03 |  | 1.656e-03 |  | 7.769e-03 |  | 4.158e-03 |  | 1.906e-03 |  | 0.031 |  | 5.199e-03 |  | 1.663e-03 |  | 2.230-03 |  | 2.132e-03 |  | 6.892e-04 |  | 2.470e-03 |
|  | Education × time |  | 1.384e-03 |  | 3.877e-04 |  | 5.220e-04 |  | 1.125e-03 |  | 4.462e-04 |  | 0.013 |  | 7.893e-04 |  | 3.892e-04 |  | 0.045 |  | 4.042e-04 |  | 1.613e-04 |  | 0.014 |
|  | *APOE ɛ4* × time |  | -2.649e-03 |  | 1.758e-03 |  | 0.135 |  | -4.010e-03 |  | 2.023e-03 |  | 0.050 |  | -4.350e-03 |  | 1.764e-03 |  | 0.015 |  | -1.073e-03 |  | 7.314e-04 |  | 0.145 |
|  | ICV × time |  | 1.001e-08 |  | 6.252e-09 |  | 0.112 |  | 1.051e-08 |  | 7.195e-09 |  | 0.147 |  | 4.149e-09 |  | 6.276e-09 |  | 0.510 |  | -7.931e-10 |  | 2.601e-09 |  | 0.761 |
|  | Group high × time |  | 4.059e-03 |  | 1.812e-03 |  | **0.027** |  | 4.027e-03 |  | 2.085e-03 |  | 0.056 |  | 1.753e-03 |  | 1.818e-03 |  | 0.337 |  | 3.990e-04 |  | 7.537e-04 |  | 0.598 |
| A+HTN+ |  |  | MMSE | | | | |  | FAQ | | | | |  | ADAS13 | | | | |  | \ | | | | |
|  | Predictors |  | Coefficients |  | SE |  | *P* value |  | Coefficients |  | SE |  | *P* value |  | Coefficients |  | SE |  | *P* value |  | Coefficients |  | SE |  | *P* value |
|  | Age × time |  | -0.029 |  | 0.021 |  | 0.162 |  | 0.087 |  | 0.027 |  | 1.850e-03 |  | -0.058 |  | 0.049 |  | 0.237 |  | \ |  | \ |  | \ |
|  | Female × time |  | 0.780 |  | 0.246 |  | 1.760e-03 |  | -0.832 |  | 0.323 |  | 0.011 |  | -1.987 |  | 0.594 |  | 9.860e-04 |  | \ |  | \ |  | \ |
|  | Education × time |  | -0.013 |  | 0.029 |  | 0.663 |  | -0.095 |  | 0.038 |  | 0.013 |  | 0.050 |  | 0.070 |  | 0.475 |  | \ |  | \ |  | \ |
|  | *APOE ɛ4* × time |  | -0.522 |  | 0.189 |  | 6.240e-03 |  | 1.213 |  | 0.250 |  | 2.500e-06 |  | 1.654 |  | 0.446 |  | 2.690e-04 |  | \ |  | \ |  | \ |
|  | Group high × time |  | -0.748 |  | 0.239 |  | **2.040e-03** |  | 1.673 |  | 0.314 |  | **2.900e-07** |  | 0.259 |  | 0.558 |  | 0.643 |  | \ |  | \ |  | \ |
|  |  |  | MEM | | | | |  | LAN | | | | |  | EF | | | | |  | VP | | | | |
|  | Predictors |  | Coefficients |  | SE |  | *P* value |  | Coefficients |  | SE |  | *P* value |  | Coefficients |  | SE |  | *P* value |  | Coefficients |  | SE |  | *P* value |
|  | Age × time |  | -7.660e-04 |  | 1.905e-03 |  | 0.688 |  | -5.465e-04 |  | 2.438e-03 |  | 0.823 |  | 6.436e-03 |  | 2.656e-03 |  | 0.016 |  | 6.301e-03 |  | 3.947e-03 |  | 0.113 |
|  | Female × time |  | 0.155 |  | 0.023 |  | 1.570e-10 |  | 0.112 |  | 0.029 |  | 1.860e-04 |  | 0.113 |  | 0.032 |  | 5.400e-04 |  | 0.039 |  | 0.056 |  | 0.490 |
|  | Education × time |  | 5.125e-03 |  | 2.661e-03 |  | 0.056 |  | -6.201e-03 |  | 3.412e-03 |  | 0.071 |  | -0.015 |  | 3.716e-03 |  | 1.010e-04 |  | -5.782e-03 |  | 7.054e-03 |  | 0.414 |
|  | *APOE ɛ4* × time |  | -0.097 |  | 0.018 |  | 1.260e-07 |  | -0.119 |  | 0.022 |  | 3.330e-07 |  | -0.093 |  | 0.025 |  | 1.860e-04 |  | -0.028 |  | 0.039 |  | 0.468 |
|  | Group high × time |  | -0.054 |  | 0.022 |  | 0.015 |  | -3.207e-03 |  | 0.028 |  | 0.909 |  | -9.120e-03 |  | 0.031 |  | 0.766 |  | -0.069 |  | 0.046 |  | 0.134 |
|  |  |  | Hippocampus | | | | |  | Entorhinal cortex | | | | |  | Mid temporal lobe | | | | |  | Whole brain | | | | |
|  | Predictors |  | Coefficients |  | SE |  | *P* value |  | Coefficients |  | SE |  | *P* value |  | Coefficients |  | SE |  | *P* value |  | Coefficients |  | SE |  | *P* value |
|  | Age × time |  | -3.321e-04 |  | 1.414e-04 |  | 0.021 |  | 1.025e-04 |  | 1.411e-04 |  | 0.469 |  | 3.861e-04 |  | 1.277e-04 |  | 3.160e-03 |  | 1.535e-04 |  | 4.954e-05 |  | 2.500e-03 |
|  | Female × time |  | 5.045e-04 |  | 2.441e-03 |  | 0.837 |  | 2.320e-04 |  | 2.435e-03 |  | 0.924 |  | -2.318e-03 |  | 2.204e-03 |  | 0.295 |  | -4.069e-04 |  | 8.549e-04 |  | 0.635 |
|  | Education × time |  | 4.230e-04 |  | 2.798e-04 |  | 0.134 |  | -3.111e-04 |  | 2.791e-04 |  | 0.268 |  | -4.799e-04 |  | 2.527e-04 |  | 0.060 |  | -8.045e-05 |  | 9.800e-05 |  | 0.414 |
|  | *APOE ɛ4* × time |  | -5.598e-03 |  | 1.915e-03 |  | 4.248e-03 |  | -4.467e-03 |  | 1.910e-03 |  | 0.021 |  | 7.708e-04 |  | 1.729e-03 |  | 0.657 |  | 2.075e-04 |  | 6.706e-04 |  | 0.758 |
|  | ICV × time |  | -1.136e-08 |  | 7.978e-09 |  | 0.158 |  | 1.095e-08 |  | 7.959e-09 |  | 0.172 |  | 1.217e-08 |  | 7.205e-09 |  | 0.094 |  | 1.142e-09 |  | 2.794e-09 |  | 0.684 |
|  | Group high × time |  | -7.953e-04 |  | 1.739e-03 |  | 0.648 |  | -4.328e-03 |  | 1.735e-03 |  | **0.014** |  | -2.191e-03 |  | 1.570e-03 |  | 0.166 |  | -1.100e-03 |  | 6.090e-04 |  | 0.074 |

The primary effects of the predictive factors (i.e., age, sex, years of education, *APOE ɛ4* status, years since baseline, and ICV when appropriate) were incorporated into all linear mixed-effects models, while coefficients are not shown for the sake of brevity.

1. Abbreviations: CSF, cerebrospinal fluid; A-HTN-: A negative and normotension; A-HTN+: A positive and hypertension; A+HTN-: A positive and normotension; A+HTN+: A positive and hypertension; MMSE, Mini-Mental State Examination; FAQ, Functional Assessment Questionnaire; ADAS13, Alzheimer’s disease Assessment Scale 13; MEM, memory function; LAN, language; EF, executive function; VP, visuospatial functioning; *APOE ε4*, apolipoprotein *E4*; ICV, Intracranial volume; SE, Standard Error; Group high, CSF YKL-40 ≥ 390ng/ml (determined by the median concentration).

**Table S18** Baseline CSF YKL-40 and longitudinal changes in cognition and MRI brain structures stratified by A status and clinical diagnosis.

| A-CN |  |  | MMSE | | | | |  | FAQ | | | | |  | ADAS13 | | | | |  | \ | | | | |
| --- | --- | --- | --- | --- | --- | --- | --- | --- | --- | --- | --- | --- | --- | --- | --- | --- | --- | --- | --- | --- | --- | --- | --- | --- | --- |
|  | Predictors |  | Coefficients |  | SE |  | *P* value |  | Coefficients |  | SE |  | *P* value |  | Coefficients |  | SE |  | *P* value |  | Coefficients |  | SE |  | *P* value |
|  | Age × time |  | -0.012 |  | 4.27e-03 |  | 0.004 |  | 0.018 |  | 0.007 |  | 0.010 |  | 0.038 |  | 0.015 |  | 0.013 |  | \ |  | \ |  | \ |
|  | Female × time |  | 6.37e-04 |  | 0.050 |  | 0.990 |  | 0.015 |  | 0.082 |  | 0.855 |  | -0.329 |  | 0.175 |  | 0.062 |  | \ |  | \ |  | \ |
|  | Education × time |  | -0.021 |  | 6.75e-03 |  | 0.002 |  | 0.014 |  | 0.011 |  | 0.218 |  | 0.041 |  | 0.024 |  | 0.087 |  | \ |  | \ |  | \ |
|  | *APOE ɛ4* × time |  | 0.103 |  | 0.068 |  | 0.132 |  | -0.131 |  | 0.110 |  | 0.237 |  | -0.106 |  | 0.252 |  | 0.674 |  | \ |  | \ |  | \ |
|  | Group high × time |  | 0.077 |  | 0.059 |  | 0.191 |  | 0.060 |  | 0.097 |  | 0.535 |  | -0.344 |  | 0.223 |  | 0.125 |  | \ |  | \ |  | \ |
|  |  |  | MEM | | | | |  | LAN | | | | |  | EF | | | | |  | VP | | | | |
|  | Predictors |  | Coefficients |  | SE |  | *P* value |  | Coefficients |  | SE |  | *P* value |  | Coefficients |  | SE |  | *P* value |  | Coefficients |  | SE |  | *P* value |
|  | Age × time |  | -0.001 |  | 0.001 |  | 0.246 |  | 0.003 |  | 0.001 |  | 0.033 |  | -7.83e-04 |  | 1.19e-03 |  | 0.510 |  | 9.678e-04 |  | 1.470e-03 |  | 0.512 |
|  | Female × time |  | 0.012 |  | 0.013 |  | 0.368 |  | 0.018 |  | 0.016 |  | 0.284 |  | 0.040 |  | 0.015 |  | 0.009 |  | 0.016 |  | 0.023 |  | 0.485 |
|  | Education × time |  | -0.003 |  | 0.002 |  | 0.074 |  | -0.003 |  | 0.002 |  | 0.140 |  | -3.86e-03 |  | 1.99e-03 |  | 0.053 |  | 2.887e-03 |  | 3.371e-03 |  | 0.393 |
|  | *APOE ɛ4* × time |  | 0.020 |  | 0.017 |  | 0.227 |  | 0.012 |  | 0.020 |  | 0.562 |  | 0.016 |  | 0.019 |  | 0.392 |  | -0.044 |  | 0.027 |  | 0.101 |
|  | Group high × time |  | -0.013 |  | 0.016 |  | 0.434 |  | -0.015 |  | 0.020 |  | 0.450 |  | 0.040 |  | 0.019 |  | **0.033** |  | -0.037 |  | 0.036 |  | 0.309 |
|  |  |  | Hippocampus | | | | |  | Entorhinal cortex | | | | |  | Mid temporal lobe | | | | |  | Whole brain | | | | |
|  | Predictors |  | Coefficients |  | SE |  | *P* value |  | Coefficients |  | SE |  | *P* value |  | Coefficients |  | SE |  | *P* value |  | Coefficients |  | SE |  | *P* value |
|  | Age × time |  | -1.955e-04 |  | 9.278e-05 |  | 0.038 |  | -1.096e-04 |  | 1.001e-04 |  | 0.277 |  | -3.071e-05 |  | 7.530e-05 |  | 0.684 |  | 2.077e-05 |  | 4.536e-05 |  | 0.648 |
|  | Female × time |  | 6.997e-04 |  | 1.339e-03 |  | 0.603 |  | 3.428e-03 |  | 1.444e-03 |  | 0.020 |  | -1.208e-03 |  | 1.086e-03 |  | 0.269 |  | 3.275e-04 |  | 6.545e-04 |  | 0.618 |
|  | Education × time |  | -3.225e-04 |  | 1.891e-04 |  | 0.092 |  | -5.240e-04 |  | 2.041e-04 |  | 0.012 |  | -3.107e-04 |  | 1.535e-04 |  | 0.046 |  | -2.000e-04 |  | 9.246e-05 |  | 0.033 |
|  | *APOE ɛ4* × time |  | 3.087e-03 |  | 2.026e-03 |  | 0.131 |  | 1.259e-03 |  | 2.186e-03 |  | 0.566 |  | 8.996e-04 |  | 1.644e-03 |  | 0.586 |  | 4.231e-04 |  | 9.906e-04 |  | 0.670 |
|  | ICV × time |  | -1.776e-09 |  | 5.128e-09 |  | 0.730 |  | -1.085e-08 |  | 5.533e-09 |  | 0.053 |  | 9.201e-09 |  | 4.162e-09 |  | 0.030 |  | 4.960e-10 |  | 2.507e-09 |  | 0.844 |
|  | Group high × time |  | -1.654e-03 |  | 1.232e-03 |  | 0.183 |  | 3.345e-03 |  | 1.329e-03 |  | **0.014** |  | -4.736e-04 |  | 9.997e-04 |  | 0.637 |  | 5.791e-04 |  | 6.023e-04 |  | 0.339 |
| A-MCI |  |  | MMSE | | | | |  | FAQ | | | | |  | ADAS13 | | | | |  | \ | | | | |
|  | Predictors |  | Coefficients |  | SE |  | *P* value |  | Coefficients |  | SE |  | *P* value |  | Coefficients |  | SE |  | *P* value |  | Coefficients |  | SE |  | *P* value |
|  | Age × time |  | -2.826e-03 |  | 0.011 |  | 0.799 |  | -0.061 |  | 0.024 |  | 0.012 |  | -0.083 |  | 0.026 |  | 0.002 |  | \ |  | \ |  | \ |
|  | Female × time |  | -0.370 |  | 0.222 |  | 0.099 |  | 3.652 |  | 0.480 |  | 0.000 |  | 1.855 |  | 0.507 |  | 0.000 |  | \ |  | \ |  | \ |
|  | Education × time |  | -0.133 |  | 0.056 |  | 0.018 |  | 0.296 |  | 0.121 |  | 0.016 |  | 0.500 |  | 0.129 |  | 0.000 |  | \ |  | \ |  | \ |
|  | *APOE ɛ4* × time |  | -2.248 |  | 1.129 |  | 0.049 |  | -0.250 |  | 2.434 |  | 0.918 |  | 0.662 |  | 2.531 |  | 0.794 |  | \ |  | \ |  | \ |
|  | Group high × time |  | 0.186 |  | 0.201 |  | 0.358 |  | -0.992 |  | 0.436 |  | **0.024** |  | 0.058 |  | 0.456 |  | 0.899 |  | \ |  | \ |  | \ |
|  |  |  | MEM | | | | |  | LAN | | | | |  | EF | | | | |  | VP | | | | |
|  | Predictors |  | Coefficients |  | SE |  | *P* value |  | Coefficients |  | SE |  | *P* value |  | Coefficients |  | SE |  | *P* value |  | Coefficients |  | SE |  | *P* value |
|  | Age × time |  | -9.003e-04 |  | 1.536e-03 |  | 0.559 |  | -0.002 |  | 0.001 |  | 0.259 |  | -0.002 |  | 0.002 |  | 0.189 |  | -0.014 |  | 0.007 |  | 0.042 |
|  | Female × time |  | -0.087 |  | 0.030 |  | 0.004 |  | -0.020 |  | 0.029 |  | 0.500 |  | -0.049 |  | 0.034 |  | 0.149 |  | 0.301 |  | 0.274 |  | 0.278 |
|  | Education × time |  | -0.035 |  | 7.756e-03 |  | 1.510e-05 |  | -0.013 |  | 0.007 |  | 0.095 |  | 0.002 |  | 0.009 |  | 0.834 |  | -0.024 |  | 0.028 |  | 0.387 |
|  | *APOE ɛ4* × time |  | -0.151 |  | 0.150 |  | 0.315 |  | -0.083 |  | 0.149 |  | 0.578 |  | 0.027 |  | 0.169 |  | 0.873 |  | -0.218 |  | 0.293 |  | 0.460 |
|  | Group high × time |  | 0.037 |  | 0.027 |  | 0.174 |  | 0.031 |  | 0.026 |  | 0.251 |  | 0.076 |  | 0.031 |  | **0.014** |  | 0.083 |  | 0.070 |  | 0.245 |
|  |  |  | Hippocampus | | | | |  | Entorhinal cortex | | | | |  | Mid temporal lobe | | | | |  | Whole brain | | | | |
|  | Predictors |  | Coefficients |  | SE |  | *P* value |  | Coefficients |  | SE |  | *P* value |  | Coefficients |  | SE |  | *P* value |  | Coefficients |  | SE |  | *P* value |
|  | Age × time |  | 1.687e-04 |  | 1.911e-04 |  | 0.381 |  | 3.378e-04 |  | 1.595e-04 |  | 0.038 |  | -1.478e-05 |  | 1.056e-04 |  | 0.889 |  | 4.398e-05 |  | 5.881e-05 |  | 0.457 |
|  | Female × time |  | -7.764e-04 |  | 2.948e-03 |  | 0.793 |  | 4.790e-03 |  | 2.462e-03 |  | 0.056 |  | 3.159e-03 |  | 1.629e-03 |  | 0.057 |  | -7.916e-06 |  | 9.076e-04 |  | 0.993 |
|  | Education × time |  | -6.622e-05 |  | 6.800e-04 |  | 0.923 |  | -5.007e-04 |  | 5.677e-04 |  | 0.381 |  | -5.561e-05 |  | 3.756e-04 |  | 0.883 |  | 4.829e-05 |  | 2.093e-04 |  | 0.818 |
|  | *APOE ɛ4* × time |  | -8.554e-03 |  | 5.352e-03 |  | 0.115 |  | -4.001e-03 |  | 4.469e-03 |  | 0.374 |  | 2.808e-03 |  | 2.957e-03 |  | 0.346 |  | 1.292e-03 |  | 1.647e-03 |  | 0.436 |
|  | ICV × time |  | 2.736e-08 |  | 1.074e-08 |  | 0.013 |  | 2.260e-08 |  | 8.969e-09 |  | 0.014 |  | 4.914e-09 |  | 5.934e-09 |  | 0.411 |  | -1.379e-09 |  | 3.307e-09 |  | 0.678 |
|  | Group high × time |  | -8.251e-03 |  | 2.688e-03 |  | **3.210e-03** |  | -1.174e-02 |  | 2.244e-03 |  | **2.240e-06** |  | -3.557e-03 |  | 1.485e-03 |  | **0.020** |  | -1.062e-03 |  | 8.274e-04 |  | 0.204 |
| A+CN |  |  | MMSE | | | | |  | FAQ | | | | |  | ADAS13 | | | | |  | \ | | | | |
|  | Predictors |  | Coefficients |  | SE |  | *P* value |  | Coefficients |  | SE |  | *P* value |  | Coefficients |  | SE |  | *P* value |  | Coefficients |  | SE |  | *P* value |
|  | Age × time |  | 0.029 |  | 0.017 |  | 0.100 |  | -0.086 |  | 0.029 |  | 0.004 |  | -0.044 |  | 0.031 |  | 0.163 |  | \ |  | \ |  | \ |
|  | Female × time |  | 0.767 |  | 0.303 |  | 0.013 |  | -1.613 |  | 0.502 |  | 0.002 |  | -1.323 |  | 0.531 |  | 0.014 |  | \ |  | \ |  | \ |
|  | Education × time |  | 0.025 |  | 0.038 |  | 0.514 |  | -0.094 |  | 0.062 |  | 0.133 |  | -0.090 |  | 0.067 |  | 0.184 |  | \ |  | \ |  | \ |
|  | *APOE ɛ4* × time |  | -0.047 |  | 0.195 |  | 0.811 |  | 0.696 |  | 0.328 |  | 0.036 |  | 0.581 |  | 0.350 |  | 0.099 |  | \ |  | \ |  | \ |
|  | Group high × time |  | 0.469 |  | 0.260 |  | 0.074 |  | -1.462 |  | 0.431 |  | **0.001** |  | -0.988 |  | 0.457 |  | **0.033** |  | \ |  | \ |  | \ |
|  |  |  | MEM | | | | |  | LAN | | | | |  | EF | | | | |  | VP | | | | |
|  | Predictors |  | Coefficients |  | SE |  | *P* value |  | Coefficients |  | SE |  | *P* value |  | Coefficients |  | SE |  | *P* value |  | Coefficients |  | SE |  | *P* value |
|  | Age × time |  | 0.007 |  | 0.002 |  | 0.003 |  | 0.003 |  | 0.002 |  | 0.249 |  | 0.007 |  | 0.003 |  | 0.009 |  | -0.001 |  | 0.004 |  | 0.746 |
|  | Female × time |  | 0.153 |  | 0.039 |  | 0.000 |  | 0.095 |  | 0.037 |  | 0.013 |  | 0.046 |  | 0.043 |  | 0.287 |  | -0.015 |  | 0.076 |  | 0.840 |
|  | Education × time |  | 0.010 |  | 0.005 |  | 0.051 |  | 0.003 |  | 0.005 |  | 0.564 |  | -0.004 |  | 0.006 |  | 0.510 |  | -0.005 |  | 0.009 |  | 0.593 |
|  | *APOE ɛ4* × time |  | -0.048 |  | 0.026 |  | 0.063 |  | -0.016 |  | 0.025 |  | 0.525 |  | -0.009 |  | 0.029 |  | 0.760 |  | -0.016 |  | 0.041 |  | 0.706 |
|  | Group high × time |  | 0.055 |  | 0.033 |  | 0.100 |  | 0.056 |  | 0.032 |  | 0.083 |  | -0.018 |  | 0.037 |  | 0.639 |  | -0.067 |  | 0.059 |  | 0.262 |
|  |  |  | Hippocampus | | | | |  | Entorhinal cortex | | | | |  | Mid temporal lobe | | | | |  | Whole brain | | | | |
|  | Predictors |  | Coefficients |  | SE |  | *P* value |  | Coefficients |  | SE |  | *P* value |  | Coefficients |  | SE |  | *P* value |  | Coefficients |  | SE |  | *P* value |
|  | Age × time |  | 1.228e-05 |  | 2.191e-04 |  | 0.95557 |  | 8.980e-04 |  | 3.052e-04 |  | 0.005 |  | 5.928e-04 |  | 1.649e-04 |  | 0.001 |  | 2.152e-04 |  | 1.034e-04 |  | 0.043 |
|  | Female × time |  | 0.010 |  | 1.694e-03 |  | 4.500e-07 |  | 8.791e-03 |  | 2.360e-03 |  | 0.001 |  | 1.488e-03 |  | 1.275e-03 |  | 0.249 |  | -4.469e-04 |  | 7.997e-04 |  | 0.579 |
|  | Education × time |  | -8.894e-04 |  | 2.766e-04 |  | 0.00244 |  | -1.367e-03 |  | 3.852e-04 |  | 0.001 |  | -5.237e-04 |  | 2.081e-04 |  | 0.016 |  | -1.724e-04 |  | 1.305e-04 |  | 0.193 |
|  | *APOE ɛ4* × time |  | -8.765e-03 |  | 1.411e-03 |  | 1.630e-07 |  | -0.011 |  | 1.965e-03 |  | 6.480e-07 |  | -2.488e-03 |  | 1.061e-03 |  | 0.024 |  | -4.965e-04 |  | 6.658e-04 |  | 0.460 |
|  | ICV × time |  | -1.481e-08 |  | 6.762e-09 |  | 0.033842 |  | -2.345e-08 |  | 9.419e-09 |  | 0.017 |  | -1.541e-08 |  | 5.087e-09 |  | 0.004 |  | -6.059e-09 |  | 3.192e-09 |  | 0.064 |
|  | Group high × time |  | 3.427e-03 |  | 1.689e-03 |  | **0.04858** |  | -7.302e-03 |  | 2.353e-03 |  | **0.003** |  | -6.126e-03 |  | 1.271e-03 |  | **1.750e-05** |  | -1.842e-03 |  | 7.973e-04 |  | **0.026** |
| A+MCI |  |  | MMSE | | | | |  | FAQ | | | | |  | ADAS13 | | | | |  | \ | | | | |
|  | Predictors |  | Coefficients |  | SE |  | *P* value |  | Coefficients |  | SE |  | *P* value |  | Coefficients |  | SE |  | *P* value |  | Coefficients |  | SE |  | *P* value |
|  | Age × time |  | 0.005 |  | 0.015 |  | 0.744 |  | 0.011 |  | 0.020 |  | 0.594 |  | -0.103 |  | 0.031 |  | 0.001 |  | \ |  | \ |  | \ |
|  | Female × time |  | 0.359 |  | 0.192 |  | 0.063 |  | -0.224 |  | 0.259 |  | 0.389 |  | -1.363 |  | 0.411 |  | 0.001 |  | \ |  | \ |  | \ |
|  | Education × time |  | -0.040 |  | 0.035 |  | 0.242 |  | -0.014 |  | 0.046 |  | 0.761 |  | 0.066 |  | 0.071 |  | 0.356 |  | \ |  | \ |  | \ |
|  | *APOE ɛ4* × time |  | -0.160 |  | 0.250 |  | 0.522 |  | 0.095 |  | 0.339 |  | 0.779 |  | 1.181 |  | 0.522 |  | 0.025 |  | \ |  | \ |  | \ |
|  | Group high × time |  | -0.557 |  | 0.168 |  | **0.001** |  | 0.258 |  | 0.226 |  | 0.256 |  | -0.006 |  | 0.359 |  | 0.987 |  | \ |  | \ |  | \ |
|  |  |  | MEM | | | | |  | LAN | | | | |  | EF | | | | |  | VP | | | | |
|  | Predictors |  | Coefficients |  | SE |  | *P* value |  | Coefficients |  | SE |  | *P* value |  | Coefficients |  | SE |  | *P* value |  | Coefficients |  | SE |  | *P* value |
|  | Age × time |  | -5.588e-04 |  | 1.186e-03 |  | 0.638 |  | 2.168e-03 |  | 1.759e-03 |  | 0.219 |  | 0.005 |  | 0.002 |  | 0.020 |  | 0.010 |  | 0.003 |  | 0.001 |
|  | Female × time |  | 0.014 |  | 0.015 |  | 0.341 |  | 0.082 |  | 0.022 |  | 0.000 |  | 0.119 |  | 0.027 |  | 1.960e-05 |  | 0.040 |  | 0.040 |  | 0.323 |
|  | Education × time |  | -1.337e-04 |  | 2.692e-03 |  | 0.960 |  | -7.825e-03 |  | 3.998e-03 |  | 0.051 |  | -0.012 |  | 0.005 |  | 0.011 |  | -0.005 |  | 0.008 |  | 0.491 |
|  | *APOE ɛ4* × time |  | -0.067 |  | 0.020 |  | 0.001 |  | -0.079 |  | 0.029 |  | 0.008 |  | -0.039 |  | 0.035 |  | 0.267 |  | 0.016 |  | 0.058 |  | 0.781 |
|  | Group high × time |  | -0.013 |  | 0.013 |  | 0.345 |  | 7.667e-04 |  | 0.020 |  | 0.969 |  | -0.034 |  | 0.024 |  | 0.161 |  | -0.089 |  | 0.032 |  | **0.006** |
|  |  |  | Hippocampus | | | | |  | Entorhinal cortex | | | | |  | Mid temporal lobe | | | | |  | Whole brain | | | | |
|  | Predictors |  | Coefficients |  | SE |  | *P* value |  | Coefficients |  | SE |  | *P* value |  | Coefficients |  | SE |  | *P* value |  | Coefficients |  | SE |  | *P* value |
|  | Age × time |  | -1.942e-04 |  | 1.392e-04 |  | 0.165 |  | 1.136e-04 |  | 1.458e-04 |  | 0.437 |  | 4.590e-05 |  | 1.347e-04 |  | 0.734 |  | -7.979e-06 |  | 5.523e-05 |  | 0.885 |
|  | Female × time |  | 6.267e-04 |  | 1.889e-03 |  | 0.741 |  | 2.928e-03 |  | 1.979e-03 |  | 0.141 |  | 3.968e-03 |  | 1.827e-03 |  | 0.032 |  | 2.669e-03 |  | 7.493e-04 |  | 0.001 |
|  | Education × time |  | 8.206e-04 |  | 2.945e-04 |  | 0.006 |  | 9.171e-05 |  | 3.085e-04 |  | 0.767 |  | -2.862e-04 |  | 2.849e-04 |  | 0.317 |  | 4.754e-05 |  | 1.168e-04 |  | 0.685 |
|  | *APOE ɛ4* × time |  | -1.402e-03 |  | 1.870e-03 |  | 0.455 |  | -1.927e-03 |  | 1.959e-03 |  | 0.327 |  | -2.640e-03 |  | 1.809e-03 |  | 0.147 |  | -1.738e-03 |  | 7.420e-04 |  | 0.021 |
|  | ICV × time |  | -5.893e-09 |  | 6.477e-09 |  | 0.364 |  | 6.159e-09 |  | 6.785e-09 |  | 0.366 |  | -1.157e-09 |  | 6.265e-09 |  | 0.854 |  | -5.273e-09 |  | 2.569e-09 |  | 0.042 |
|  | Group high × time |  | -5.686e-07 |  | 1.378e-03 |  | 0.996 |  | 6.956e-04 |  | 1.444e-03 |  | 0.631 |  | -3.242e-05 |  | 1.333e-03 |  | 0.981 |  | -2.736e-04 |  | 5.468e-04 |  | 0.618 |

The primary effects of the predictive factors (i.e., age, sex, years of education, *APOE ɛ4* status, years since baseline, and ICV when appropriate) were incorporated into all linear mixed-effects models, while coefficients are not shown for the sake of brevity.

Abbreviations: CSF, cerebrospinal fluid; A-CN: A negative and cognitively normal; A+CN: A positive and cognitively normal; A-MCI: A negative and mild cognitive impairment; A+MCI: A positive and mild cognitive impairment; MMSE, Mini-Mental State Examination; FAQ, Functional Assessment Questionnaire; ADAS13, Alzheimer’s disease Assessment Scale 13; MEM, memory function; LAN, language; EF, executive function; VP, visuospatial functioning; *APOE ε4*, apolipoprotein *E4*; ICV, Intracranial volume; SE, Standard Error; Group high, CSF YKL-40 ≥ 390ng/ml (determined by the median concentration).
